# Imine-Oxazoline (ImOx): A *C*_1_‑Symmetric *N*,*N*‑Bidentate Ligand for Asymmetric Catalysis

**DOI:** 10.1021/acscatal.5c03134

**Published:** 2025-06-17

**Authors:** Elliot S. Silk, Haozhe Zhu, Alexander G. Shtukenberg, Tianning Diao

**Affiliations:** Department of Chemistry, 5894 New York University, 100 Washington Square East, New York, New York 10003, United States

**Keywords:** chiral ligand, ImOx, asymmetric catalysis, asymmetric catalysis, palladium, nickel, redox active

## Abstract

Asymmetric catalysis relies on the design of chiral ligands, but the variety of nitrogen-based ligands remains limited. To address this gap, we have developed a class of *C*_1_-symmetric *N*,*N*-bidentate ligands, imine-oxazoline (ImOx), derived from amino acids through a four-step synthesis. ImOx features an imine moiety conjugated with a chiral oxazoline ring as a hybrid of α-diimine (ADI) and pyridine oxazoline (PyOx) ligands. Its low symmetry allows for independent optimization at both coordination sites. ImOx improves the enantioselectivity of palladium-catalyzed conjugate addition reactions, demonstrating a strong correlation between ee and the steric effects on both the imine and oxazoline sites. Studies on well-defined organopalladium intermediates reveal that the steric bulk of ImOx necessitates a cationic pathway to promote alkene insertion. Structural characterization of ImOx suggests a stronger *trans*-influence compared to PyOx. Moreover, ImOx demonstrates versatile redox activity, promoting the reduction of nickel complexes and stabilizing nickel radical complexes. We anticipate that ImOx will expand the toolkit of chiral N-ligands for asymmetric catalysis.

## Graphical abstract

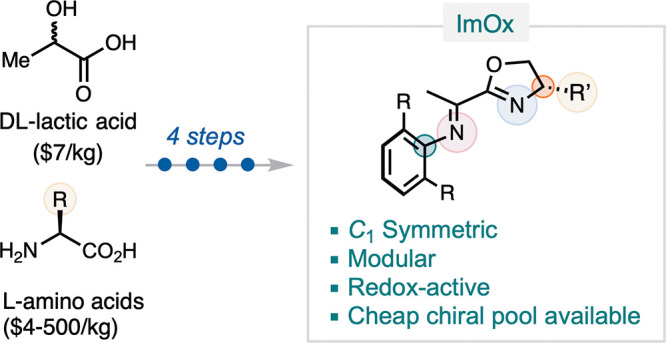

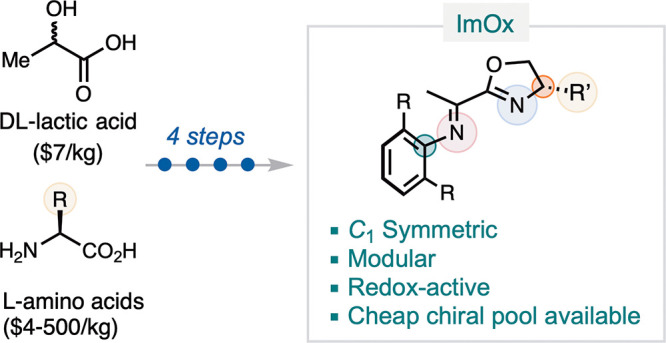

## Introduction

The stereochemical configuration of a drug can significantly affect its potency, specificity, and pharmacokinetics. Currently, over 90% of chiral molecules are synthesized using stoichiometric methods, including derivatization from chiral pools, resolution, and chiral transfer from auxiliaries and reagents.^1^ Asymmetric catalysis offers a sustainable, atom-economic, and efficient alternative for introducing chirality in pharmaceutical targets, though its scope and enantioselectivity are largely dependent on the availability of chiral ligands.^2,3^

While a large library of phosphorus ligands is commercially available, driven by the success of asymmetric hydrogenation,^4^ chiral nitrogen ligands remain underdeveloped.^5^ Among commercially available chiral ligands, there are more than four times as many chiral phosphorus ligands compared to nitrogen ligands (Scheme 1A). Recently, nitrogen ligands have significantly advanced base-metal catalysis.^6–9^ These ligands coordinate favorably to 3d metals according to the hard–soft acid–base (HSAB) principle and exhibit better stability under oxidative conditions. Moreover, pyridine and Schiff base derivatives can engage in redox activity, stabilizing metalloradical intermediates.^10^ The chirality of nitrogen ligands often leverages the naturally abundant chiral pool of amino acids,^11^ which is more straightforward compared to the methods required for synthesizing phosphorus ligands through resolution or asymmetric coupling.^12,13^

**Scheme 1:**
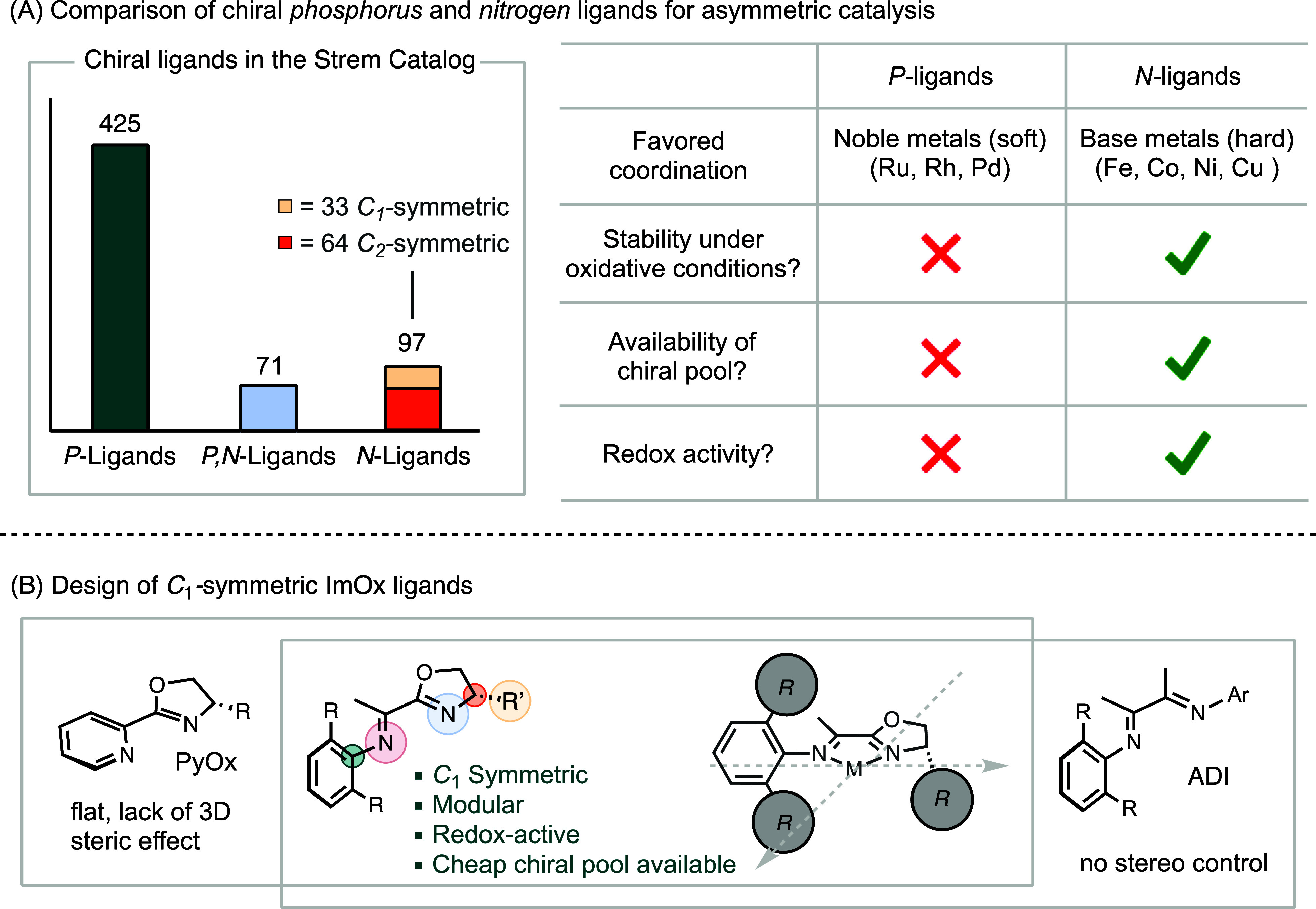
Motivation and Design Principles of Imine-Oxazoline (ImOx) Ligands for Asymmetric Catalysis

The majority of commercial *N*-based bidentate chiral ligands are *C*_2_-symmetric, such as bis-oxazoline (box) analogues.^14,15^ A lower symmetry bidentate ligand with two different coordinating sites allows for independent optimization of individual coordination sites.^16,17^ This consideration has led to the design of *C*_1_-symmetric pyridine-oxazoline (PyOx).^18,19^ Additionally, α-diimine (ADI) ligands offer versatile modularity for tuning steric and electronic effects.^20,21^ Here, we report the development of a *C*_1_-symmetric bidentate imine-oxazoline (ImOx) ligand by hybridizing the imine moiety of ADI and the chiral oxazoline component of PyOx (Scheme 1B). A related ImOx variant has been described before,^22–24^ featuring a phenyl group in the backbone. While the phenyl group is necessary for the previously developed synthetic routes, its steric hindrance limits potential applications in asymmetric catalysis.^25^ In this work, we have developed an entirely new synthetic route that enables access to the catalytically relevant ImOx variant with a methyl backbone. This highly modular ligand leverages the natural chiral pool of amino acids and is accessible in four steps. With compelling electronic and steric properties, ImOx has proven effective in facilitating enantioselective conjugate additions.^26^

## Methods

We initiated our study by developing the synthesis of ImOx scaffolds, focusing on promoting accessibility and minimizing cost. The availability of amino acids as a chiral pool and dl-lactic acid^27^ as abundant, naturally occurring building blocks prompted us to explore a route starting with the condensation of amino alcohols with dl-lactic acid to afford α-methyl oxazolyl alcohols **1** and **2** (Scheme 2).^28,29^ The oxidation of **1** and **2** proved challenging, which might account for the lack of precedents for ImOx ligands. Dess–Martin periodinate, pyridinium chlorochromate, and aerobic ABNO conditions^30,31^ appeared incompatible with the oxazoline functionality, leading to substrate decomposition. Swern oxidation of **1** or **2** with (COCl)_2_ and DMSO gave ketones **3** or **4**, respectively, in good yields. Initially, we tried to isolate and purify **3** or **4**, but observed a spontaneous rearrangement of **3** to the six-membered ring **5**.^32^ Thus, we condensed **3** or **4** with anilines without intermediate purification to afford ImOx ligands: (*S*)-^Mes^Im^*i*Pr^Ox (**6**), (*S*)-^Dipp^Im^*i*Pr^Ox (**7**), (*S*)-^Mes^Im^*t*Bu^Ox (**8**), and (*S*)-^Dipp^Im^*t*Bu^Ox (**9**) (*i*Pr = isopropyl, *t*Bu = *tert*-butyl, Mes = 2,4,6-trimethylphenyl, Dipp = 2,6-diisopropylphenyl).

**Scheme 2:**
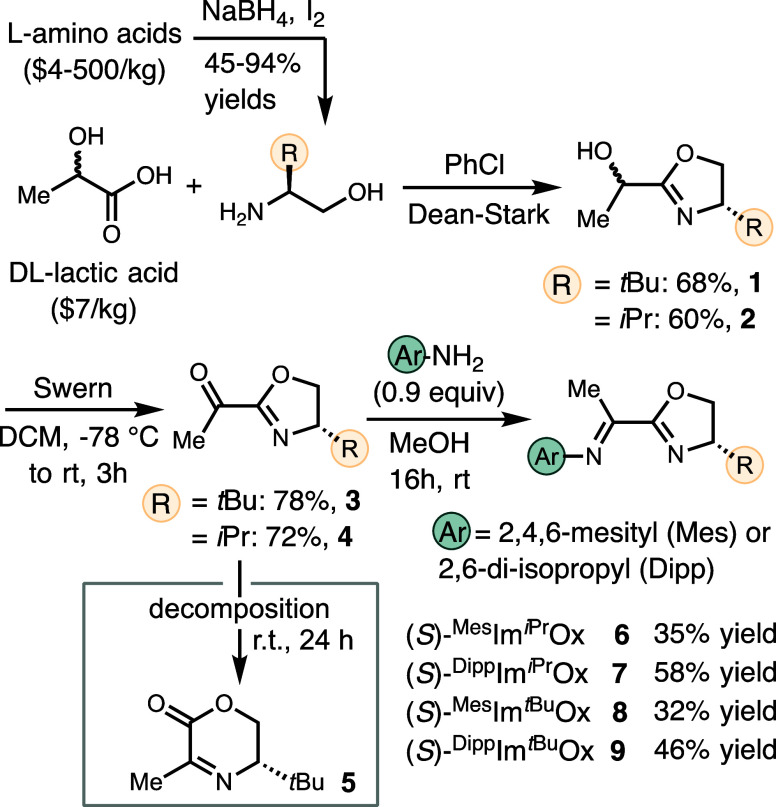
Synthesis of ImOx Ligands from Lactic Acid and Amino Acids

With a range of ImOx variants in hand, we explored their utility in asymmetric catalysis. Palladium-catalyzed conjugate addition to α,β-unsaturated enones has emerged as an effective strategy for constructing all-carbon quaternary centers. *t*BuPyOx has been reported to facilitate the synthesis of **10** with high yield and excellent ee,^33,34^ whereas its application to the synthesis of the diaryl derivative **11** resulted in modest yield and ee.^35^ We examined the performance of ImOx in these two reactions in comparison to PyOx.

Initially, we replaced *t*BuPyOx with MesIm*t*BuOx **8** in the original conditions for palladium-catalyzed asymmetric conjugate addition,^33^ but observed **10** in only 10% yield, along with the formation of biphenyl and significant catalyst decomposition into palladium black (Scheme 3A). We attributed the catalyst decomposition to the formation of a Pd-bis-aryl intermediate, followed by reductive elimination to form Pd(0). To address this competing pathway, we added AgOTf as a Lewis acid and counterion abstractor to facilitate enone coordination and subsequent insertion. AgOTf is also capable of reoxidizing Pd(0) to Pd­(II), potentially preventing Pd black formation.^36^ Furthermore, we improved the yield by introducing five equivalents of H_2_O to promote transmetalation by generating the active boronate species.^37^

**Scheme 3:**
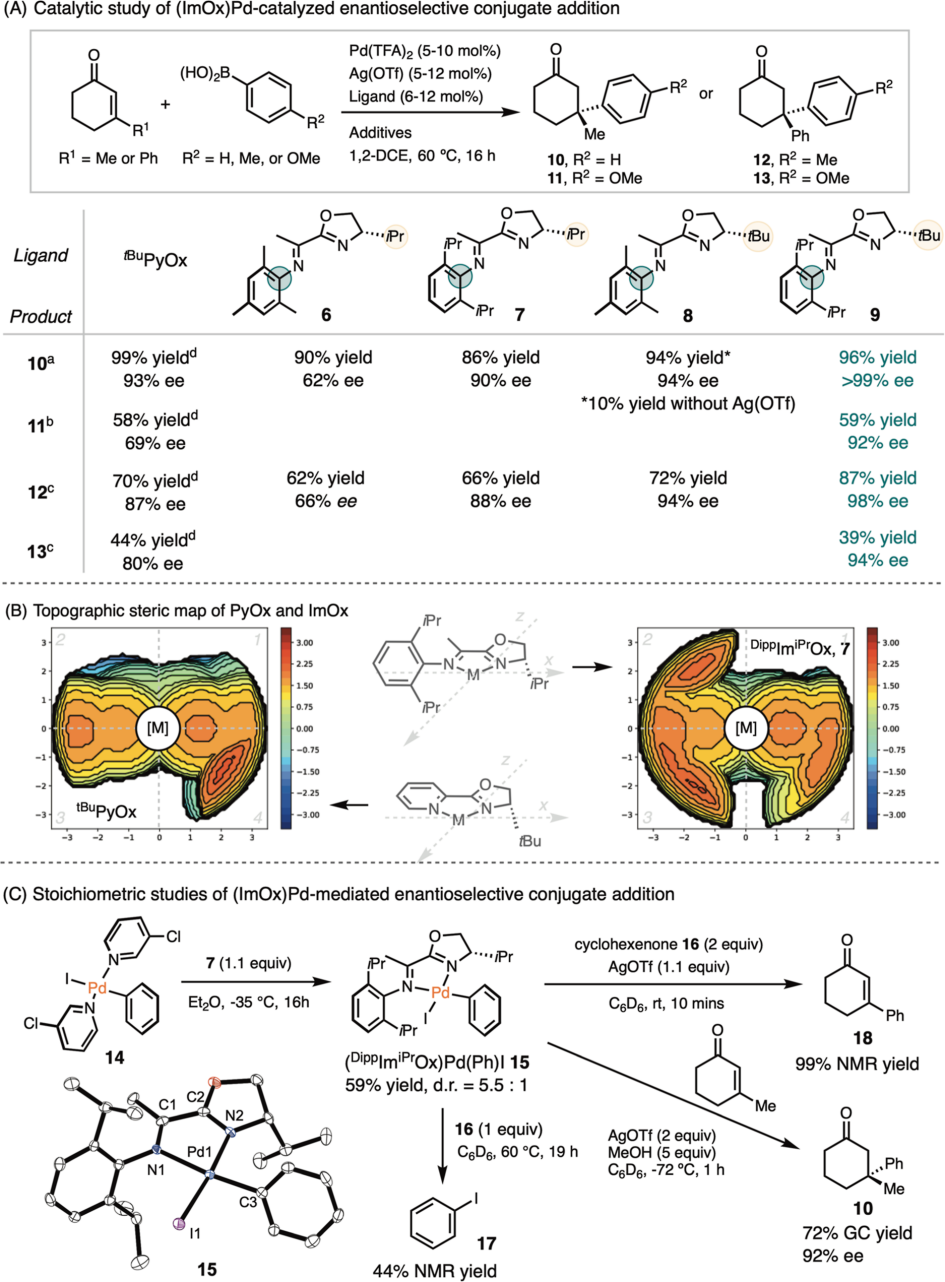
Application of ImOx to Palladium-Catalyzed Conjugate Addition to Form Quaternary Chiral Centers

a Conditions: [enone] = 0.25 M (0.250 mmol), Pd­(TFA)_2_ (5 mol %), ImOx (6 mol %), AgOTf (5 mol %), H_2_O (5 equiv).
b Conditions: [enone] = 0.25 M (0.250 mmol), Pd­(TFA)_2_ (5 mol %), ImOx (6 mol %), AgOTf (5 mol %), MeOH (5 equiv).
c Conditions: [enone] = 0.25 M (0.250 mmol), Pd­(TFA)_2_ (10 mol %), ImOx (12 mol %), AgOTf (10 mol %), Sc­(OTf)_3_ (10 mol %), 18-crown-6 (50 mol %), MeOH (5 equiv).
d Reported in refs 35 and 38 .

Comparisons among ImOx ligands **6–9** indicated that bulkier substituents on both the aryl and oxazoline moieties led to increased yield and ee (Scheme 3A). The effect of substituents on the oxazoline ring appeared to be more significant than those on the imine. Notably, (*S*)-^Dipp^Im^*t*Bu^Ox **9** delivered a quantitative yield and perfect ee, with no minor enantiomer detected by HPLC (Figure S5). Applying (*S*)-^Dipp^Im^*t*Bu^Ox **9** to a more challenging substrate, such as *para*-methoxyphenyl boronic acid nucleophile, improved the ee of **11** from 69% as previously reported^38^ to 92%.

Subsequently, we evaluated the application of ImOx to the more challenging conjugate addition of *p*-tolylboronic acid to 3-phenylcyclohexenone to afford **12** (Scheme 3A).^35^ Compared to the initial conditions reported with PyOx,^35^ the use of ImOx required the use of AgOTf as an additive to prevent catalyst decomposition and achieve high yields. Additionally, the reaction with ImOx benefited from the addition of 5 equiv of MeOH or water to facilitate transmetalation, along with Sc­(OTf)_3_ as a Lewis acid to activate the enone, and 18-crown-6 as a phase transfer catalyst. Under these optimized conditions, we evaluated the effect of ImOx variants. The trend of ligand effect was similar to that observed in the formation of **10**. Bulkier substituents on both the imine and oxazoline moieties improved the yield and ee. A comparison between (*S*)-^Mes^Im^*i*Pr^Ox **6** and (*S*)-^Dipp^Im^*i*Pr^Ox **7** revealed that replacing Mes with Dipp on the imine improved the ee from 66% to 88%. The substituent on oxazoline had an even more profound effect, increasing the ee from 66% to 94% when the *i*Pr group of (*S*)-^Mes^Im^*i*Pr^Ox **6** was switched to a *t*Bu group in (*S*)-^Mes^Im^*t*Bu^Ox **8**. A high yield of 87% and an ee of 98% were obtained with (*S*)-^Dipp^Im^*t*Bu^Ox **9**. Using (*S*)-^Dipp^Im^*t*Bu^Ox **9** with the more challenging *para*-methoxyphenyl boronic acid enhanced the ee of **13** from the previously reported 80% to 94%.

Control experiments using ^*t*Bu^PyOx under our optimized conditions afforded **10** in an 88% yield and 90% ee, and **12** in a 29% yield and 86% ee (cf. Supporting Information). These results suggest that the observed improvement in enantioselectivity arises from the ImOx ligand itself, rather than from changes in reaction conditions. Steric heat maps^39^ shed light on the differences between ImOx and ^*t*Bu^PyOx (Scheme 3B). In the case of ^*t*Bu^PyOx, the *t*Bu group on the oxazoline occupies quadrant four, leaving quadrants one, two, and three relatively open. In contrast, ImOx spans quadrants two, three, and four, thereby restricting the approach vectors of the substrate toward the metal catalyst.

While the mechanism of (PyOx)­Pd-catalyzed conjugate addition has been well-understood,^37^ we carried out organometallic studies of (ImOx)Pd for comparison. Ligand substitution of bis­(3-chloropyridine)­Pd^II^(Ph)I **14** ^40^ with (*S*)-^Dipp^Im^*i*Pr^Ox **7** resulted in the formation of (^Dipp^Im^*i*Pr^Ox)­Pd­(Ph)I **15** as a mixture of diastereomers in a ratio of 5.5:1 (Scheme 3C). NOESY experiments established that the major diastereomer has the phenyl group *trans* to imine (Figure S45). Single-crystal X-ray analysis of **15** confirmed a square planar geometry.

When we subjected **15** to one equivalent of cyclohexenone **16** and heated the mixture to 60 °C overnight, we observed formation of iodobenzene **17** in 44% yield. In the presence of 1.1 equiv of AgOTf, (^Dipp^Im^*i*Pr^Ox)­Pd­(Ph)I **15** fully converted into 3-phenylcyclohexenone **18** via insertion followed by β-H elimination. When we replaced cyclohexenone **16** with 3-methylcyclohexenone in the presence of five equivalents MeOH as a proton source, (^Dipp^Im^*i*Pr^Ox)­Pd­(Ph)I **15** converted into **10** in 72% GC yield and 92% ee.

These results highlight the effect of the steric bulk of ImOx on its reactivity. First, the reductive elimination from a (ImOx)­Pd­(Ph)­I complex is more favorable than the reverse process of oxidative addition. Second, the steric bulk prevents the direct insertion of the square-planar complex **15** into cyclohexenone, necessitating the abstraction of iodide by AgOTf to facilitate insertion by forming a cationic intermediate. A major difference of the catalytic conditions between ImOx and PyOx is the requirement for stoichiometric AgOTf to promote insertion, which proved to be the enantio-determining step.

We synthesized a series of ImOx-ligated palladium and nickel complexes to further characterize its structural and electronic properties in comparison to PyOx. Ligand substitution of (cod)­PdCl_2_ (cod = 1,5-cyclooctadiene) with ImOx proceeded smoothly to deliver (^Dipp^Im^*i*Pr^Ox)­PdCl_2_ **19** and (^Mes^Im^*t*Bu^Ox)­PdCl_2_ **20** in good yields (Scheme 4A). Single-crystal X-ray diffraction reveals a square-planar geometry for **19**, whereas the chloride adjacent to the *tert*-butyl group of ^Mes^Im^*t*Bu^Ox in **20** is slightly elevated, leading to a distortion from a square-planar geometry, possibly due to the steric hindrance. The bond lengths of C1–N1­(imine) in (ImOx)­PdCl_2_ **19** and **20** are significantly shorter than those of C1–N1­(pyridine) in (^*t*Bu^PyOx)­PdCl_2_.^41,42^ Additionally, the C1–C2 bonds, which connect the oxazoline and imine in **19** and **20**, are substantially longer than those in (^*t*Bu^PyOx)­PdCl_2_, indicating less extensive conjugation in (ImOx)­PdCl_2_ compared to (^*t*Bu^PyOx)­PdCl_2_. Although the bond lengths of Pd–N1 are comparable, the Pd–N2­(oxazoline) bonds in (ImOx)­PdCl_2_ **19** and **20** are shorter than those in (^*t*Bu^PyOx)­PdCl_2_. Correspondingly, the Pd–Cl1 and Pd–Cl2 bond lengths in (ImOx)­PdCl_2_ **19** and **20** are longer, suggesting a stronger *trans*-influence of ImOx relative to PyOx. However, the similar lengths of the Pd–Cl1 and Pd–Cl2 bonds indicate comparable *trans*-influences of the imine and oxazoline moieties.

**Scheme 4:**
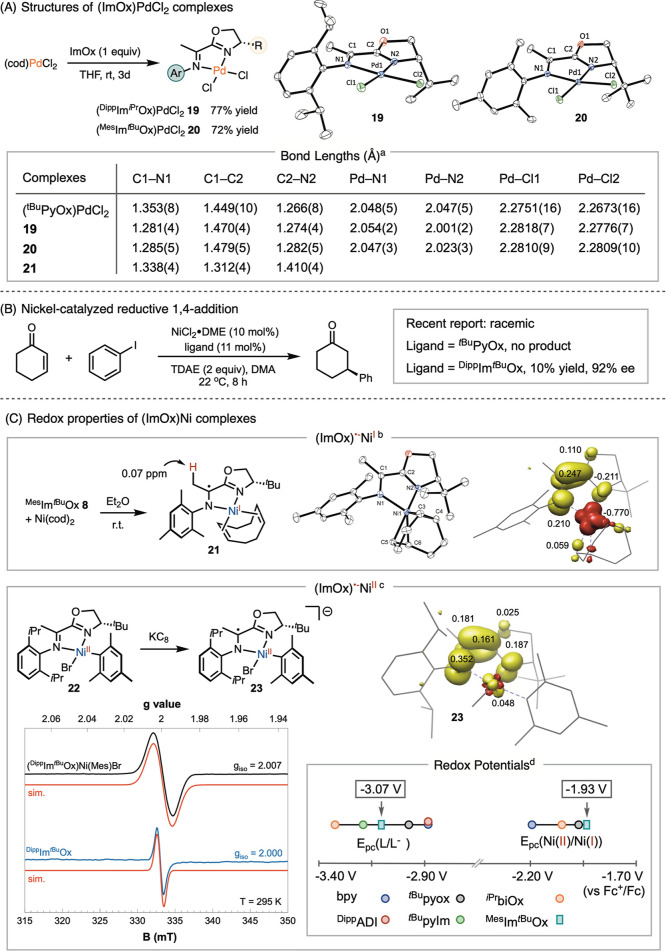
Structural and Electronic Properties of ImOx Complexes

a Bond lengths were measured by single crystal X-ray diffraction. The thermal ellipsoids are shown at 50% probability with H atoms omitted for clarity.
b DFT calculations using the ORCA package with B3LYP-D3/def2-TZVPP level of theory revealed the electronic structures of **21** and **23**. Spin density plot obtained from a Mulliken spin density population analysis. Positive spin density is shown in yellow, and negative spin density is shown in red.
c X-band ERP spectrum was performed under following conditions: temperature = 295 K, solvent = THF, microwave frequency = 9.363381 GHz, power = 0.4743 mW, modulation amplitude = 10.0 G, modulation frequency = 100 kHz.
d Redox potentials measured by cyclic voltammetry.

A preliminary investigation into the reactivity of ImOx in base-metal catalysis reveals its ability to promote the asymmetric reductive addition of aryl iodides to cyclohexenone with 92% ee (Scheme 4B), a reaction that produced nearly racemic products in previous studies.^43^ The yield was low, likely due to the slow insertion of the nickel–aryl species into cyclohexenone, as evidenced by the formation of a significant amount of biphenyl byproduct. The addition of Lewis acids, such as AgOTf, improved the yield but decreased the ee. Notably, replacing ^Dipp^Im^*t*Bu^Ox with ^*t*Bu^PyOx led to no product formation.

While the yield using catalytic nickel requires further optimization, this preliminary result prompted us to evaluate the redox properties of ImOx, focusing on its ability to stabilize low-valent nickel radical complexes, which are critical for modern nickel-catalyzed cross-coupling reactions. Ligand substitution of Ni­(cod)_2_ with ^Mes^Im^*i*Pr^Ox yielded a deep violet complex (^Mes^Im^*i*Pr^Ox)­Ni­(cod) **21**. X-ray crystallography revealed an elongation of the C1–N1 and C2–N2 bonds and a shortening of C1–C2 bond compared to those in Pd­(II) complexes, consistent with a reduction of the ImOx ligand. The ^1^H NMR spectrum of **21** reveals a resonance at 0.07 ppm, assigned to the methyl group adjacent to the imine, which is significantly shifted from its typical region around 2 ppm. DFT calculations of the electronic structure of **21** using the ORCA package resulted in a broken-symmetry BS­(1,1) solution,^44^ where a ligand-centered spin is antiferromagnetically coupled with a nickel-centered spin.^45^ These data are consistent with the electronic structure of a Ni­(I) coordinated with an ImOx radical anion. Chemical reduction of (^Dipp^Im^*t*Bu^Ox)­Ni­(Mes)Br **22** by KC_8_ resulted in an immediate color change from deep purple/red to light golden brown. The isotropic EPR signal of the reduced species **23** has a g value of 2.007, consistent with a ligand-centered radical but distinct from that of a free ligand radical anion (*g* = 2.000). DFT calculations of **23** further support the electronic structure of an ImOx-centered radical coordinated to a nickel­(II).

When measuring the reduction potentials of ImOx by cyclic voltammetry (CV) under the same conditions as other common bidentate *N*-ligands, we found that ^Mes^Im^*t*Bu^Ox is more difficult to reduce than bpy, PyOx, and ADI, yet easier than bioxazoline (biOx) and pyridine-imidazole (PyIm) (Scheme 4C). However, when comparing the nickel-mesityl bromide complexes, (ImOx)­Ni­(Mes)Br displayed the least negative reduction potential. This data highlights ImOx’s pronounced tendency to exhibit redox activity in supporting nickel radical complexes, likely contributed by two factors. The imine moiety of ImOx lacks an α-*O* or *N* atom capable of providing resonance donation, as found in biOx or biIm, and its reduction does not require disrupting aromaticity, as is necessary with bpy or phen. The result that ImOx complexes facilitate reduction of coordinated metal species helps rationalize the difference in reactivity between ImOx and PyOx for the reductive 1,4-addition in Scheme 4B.

## Conclusion

In summary, we developed a *C*_1_-symmetric *N*,*N*-bidentate ligand, ImOx, derived from the chiral pool of amino acids. ImOx improved enantioselectivity in the palladium-catalyzed conjugate addition of phenylboronic acids to β-substituted α,β-unsaturated ketones. The steric effects at both the imine and oxazoline sites strongly influence the ee and be independently optimized owing to the modular structure of ImOx. Studies of well-defined organometallic intermediates demonstrate that the imine and oxazoline moieties in ImOx exhibit a comparable *trans*-influence, which is stronger than that of PyOx. Moreover, ImOx shows excellent redox activity, promoting the reduction of nickel complexes and stabilizing nickel radicals. We anticipate that ImOx will enrich the toolbox of chiral *N*-ligands for asymmetric catalysis.

## Supplementary Material















## Notes

All experimental procedures, additional figures, details of compound synthesis, characterization, and NMR spectra (PDF)
X-ray crystallographic data for **21** (CIF)
X-ray crystallographic data for **15** (CIF)
X-ray crystallographic data for **19** (CIF)
X-ray crystallographic data for **20** (CIF)
X-ray crystallographic data for **S1** (CIF)
X-ray crystallographic data for **22** (CIF)
