# Supplementary material for: Imine-Oxazoline (ImOx): A C 1‑Symmetric N,N‑Bidentate Ligand for Asymmetric Catalysis
Source: ACS Catal. 2025 Jun 17;15(13):11349–55. doi: 10.1021/acscatal.5c03134 (PMC12235631; doi:10.1021/acscatal.5c03134)
Supplement: Supplementary file 1 [file cs5c03134_si_001.pdf]

Supporting Information

Imine-Oxazoline (ImOx): A C_1-Symmetric N,N-Bidentate Ligand for Asymmetric Catalysis

Elliot S. Silk[1], Haozhe Zhu[1], Alexander G. Shtukenberg[1], and Tianning Diao[1]*

[1]Department of Chemistry, New York University, 100 Washington Square East

New York, NY 10003, United States

*E-mail: diao@nyu.edu

Table of Contents

1. Procedures, Materials, and Instrumentation 3
2. Synthesis of ImOx Ligands 4
3. Enantioselective Conjugate Addition of Boronic Acids to 3-Substituted Cyclohexenone 10
4. Synthesis of Palladium Complexes 31
5. Synthesis of Nickel Complexes 33
6. Decomposition of 3 to form 5 37
7. Stoichiometric Experiments 39
8. Conjugate addition to 3-methylcyclohexenone with NH_4PF_6 44
10. NMR Spectra 45
11. UV-Vis Spectra 62
12. Cyclic Voltammetry Data 65
13. EPR Data 68
14. FT-IR Spectra 69
15. Single-Crystal Structure Determination 75
16. DFT Calculations 83
17. References 88

1. Procedures, Materials, and Instrumentation

1.1 General Considerations

All air- and moisture- sensitive manipulations were carried out in a nitrogen-filled MBraun glove box or under a nitrogen atmosphere using standard Schlenk techniques. Solvents were dried and deoxygenated by a Grubbs' type solvent purification system. Unless otherwise noted, metals, substrates, and ligands were purchased from commercial suppliers. (cod)$PdCl_2$,[1] 3-phenylcyclohexenone,[2] and dme•$NiBr_2$[3] were synthesized according to literature procedures. 1,2-dichloroethane, 2,4,6-trimethylaniline, and 2,6-diisopropylaniline were dried over CaH_2 for one day and distilled prior to use. Cyclohexanone, 3-chloropyridine, and phenyliodide were dried over CaH_2 for one day, distilled, degassed, and stored in a nitrogen-filled glovebox. Benzene-d_6 was purchased from Cambridge Isotope Laboratories, dried over molten Na^0 for two days, distilled, degassed, and stored in a nitrogen-filled glove box over 4 Å molecular sieves.

1.2 Materials

Reactions were monitored by thin-layer chromatography (TLC) on Merck TLC silica gel 60 F254 plates and compounds were visualized by UV light (254 nm and 365 nm) or staining with cerium molybdate (Hanessian's Stain). Column chromatography was performed using silica gel (40-53 µm, 60 Å).

1.3 Instrumentation and Software

^{1}H and ^{13}C NMR spectra were recorded on Bruker 400, 500, and 600 MHz Advance spectrometers. The cryoprobe is supported by NIH under grant number S10 OD016343. Chemical shifts are reported in parts per million (ppm) relative to residual deuterated solvent resonances (benzene-d_6, δ = 7.16 ppm; chloroform-d, δ = 7.26 ppm; acetonitrile-d_3, δ = 1.94 ppm) as the internal reference for ^{1}H NMR spectra. ^{13}C NMR chemical shifts are reported in ppm relative to residual deuterated solvent resonances (benzene-d_6, δ = 128.06 ppm; chloroform-d, δ = 77.16 ppm; acetonitrile-d_3, δ = 118.26 ppm, 1.320 ppm) as the internal reference. Spectra are reported as follows: chemical shift (δ ppm), multiplicity (br s = broad singlet, s = singlet, d = doublet, t = triplet, q = quartet, m = multiplet), coupling constant (Hz), and integration. High resolution mass spectra (HRMS) were collected on an Agilent 6224 TOF LC/MS. UV-Vis spectra were recorded on a Agilent Cary 3500 UV-Visible spectrophotometer. Infrared (IR) spectra were recorded on a Nicolet iS50 FTIR. Single crystal diffraction data were recorded on either a Bruker D8 APEX-II diffractometer using Mo-$K\alpha$ radiation or a Bruker D8 VENTURE diffractometer using Mo-$K\alpha$ or Cu-$K\alpha$ radiation. Optical rotations were recorded on a Jasco Polarimeter. Analytical chiral HPLC analyses were recorded on a Agilent 1260 Infinity using either a Chiralpak IA-3 column (4.6 mm I.D. x 250 mm L) or a Chiracel OJ-H (4.6 mm I.D. x 250 mm L) obtained from Diacel Chemical Instustries, Ltd. Gas chromatography/mass spectrometry (GC/MS) data was collected using Shimadzu GC-2010 Plus and GCMS-QP2010 SE, respectively. EPR measurements were taken on a Bruker ELEXSYS E500 Spectrometer System in continuous-wave mode in the X-band frequency using a Bruker ER 4122SHQE resonator. The spectra were simulated with Easyspin in MATLAB or Xepr software. All DFT calculations were carried out using the Gaussian 16 program or the ORCA 6.0.0[4] on NYU GREENE supercomputers. Vibrational frequency calculations were performed for all stationary points to confirm if each optimized structure is a local minimum.

1.4 Abbreviations

cod = 1,5-cyclooctadiene; ^{i}Pr = isopropyl; ^{t}Bu = *tert*-butyl; mes = 2,4,6-trimethylphenyl; dipp = 2,6-diisopropylphenyl; ^{Mes}Im^{tBu}Ox = (*S*,*E*)-1-(4-(tert-butyl)-4,5-dihydrooxazol-2-yl)-N-mesitylethan-1-imine; ^{Mes}Im^{iPr}Ox = (*S*,*E*)-1-(4-isopropyl-4,5-dihydrooxazol-2-yl)-N-mesitylethan-1-imine; ^{Dipp}Im^{tBu}Ox = (*S*,*E*)-1-(4-(*tert*-butyl)-4,5-dihydrooxazol-2-yl)-*N*-(2,6-

diisopropylphenyl)ethan-1-imine; $^{Dipp}Im^{iPr}Ox$ = (*S*,*E*)-*N*-(2,6-diisopropylphenyl)-1-(4-isopropyl-4,5-dihydrooxazol-2-yl)ethan-1-imine; NMR = nuclear magnetic spectroscopy; UV-Vis = ultraviolet-visible spectroscopy; SC-XRD = single-crystal X-ray diffraction; HRMS = high-resolution mass spectroscopy; IR = infrared spectroscopy.

2. Synthesis of ImOx Ligands

General Procedure A:

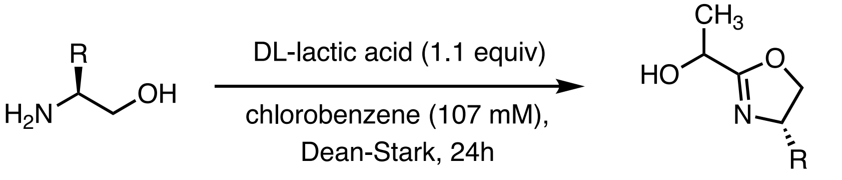

R = *t*Bu: 68% yield
R = *i*Pr: 60% yield

The procedure was performed in analogy to previously reported conditions.[5, 6] To a flame-dried round bottom flask charged with a PTFE-coated stir bar was added chlorobenzene (800 mL, 107 mM), amino alcohol (85.3 mmol, 1 equiv), and DL-lactic acid (93.9 mmol, 1.1 equiv). The flask was fit with a Dean-Stark apparatus and heated to reflux using a sand-filled heating mantle, insulating the flask and neck of the Dean-Stark apparatus with aluminum foil. After 24 hours, the flask was cooled to room temperature and solvent was removed *in vacuo* or under a stream of N_2 gas overnight resulting in a translucent clear/white paste. The crude mixture was purified by vacuum distillation (boiling point 50°C—55°C at 280 mTorr) to give the corresponding hydroxyoxazoline.

1-((*S*)-4-(*tert*-butyl)-4,5-dihydrooxazol-2-yl)ethan-1-ol **(1)**

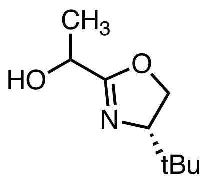

The title compound was synthesized according to General Procedure A. Product collected as a low-melting white crystalline solid (9.9 g, 68% yield).

^{1}H NMR (400 MHz, Chloroform-*d*): δ 4.41 (, 1H), 4.29 (m, 1H), 4.17 (m, 1H), 3.87 (m, 1H), 2.99 (br s, 1H), 1.41 (d, *J* = 6.7, 3H), 0.89 (s, 9H).*Note: splitting and J-coupling cannot accurately be assigned due to overlap of diastereomers.

^{13}C{^{1}H} NMR (101 MHz, Chloroform-*d*): δ 169.9, 75.2, 75.0, 70.3, 63.7, 33.7, 25.83, 25.76, 21.3, 21.2.

HRMS (ESI) m/z: $[M + H]^+$ Calculated for $C_9H_{18}NO_2^+$ 172.1338; Found 172.1377.

FT-IR: (cm^{-1}, neat, ATR) ṽ **=** 3283.72, 2957.46, 1604.54, 1463.86, 1367.48, 952.84.

1-((*S*)-4-isopropyl-4,5-dihydrooxazol-2-yl)ethan-1-ol **(2)**

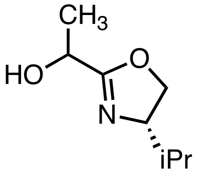

The title compound was synthesized according to General Procedure A on a 48.5 mmol scale. Product collected as a low-melting white crystalline solid (3.60g, 60% yield).

^{1}H NMR (400 MHz, Chloroform-*d*): δ 4.37 (m, 1H), 4.26 (m, 1H), 3.99 (m, 1H), 3.87 (m, 1H), 1.70 (m, 1H), 1.36 (d, *J* = 6.8, 3H), 0.91 (d, *J* = 6.8, 3H), 0.83 (d, *J* = 6.8, 1.6 Hz, 3H). *Note: splitting and J-coupling cannot accurately be assigned due to overlap of diastereomers.

^{13}C{^{1}H} NMR (101 MHz, Chloroform-*d*): δ 170.1, 71.7, 71.5, 63.7, 32.6, 21.2, 18.7, 18.0.

HRMS (ESI) m/z: $[M + H]^+$ Calculated for $C_8H_{16}NO_2^+$ 158.1181; Found 158.1165.

FT-IR: (cm^{-1}, neat, ATR) ṽ = 3225.34, 2959.67, 1665.69, 1464.86, 1117.21, 957.5.

General Procedure B:

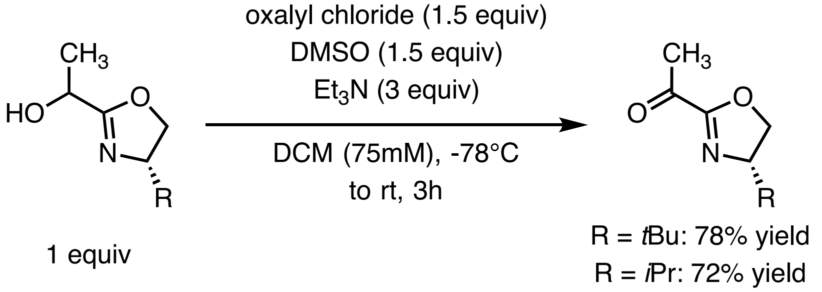

To an oven-dried round bottom flask charged with a PTFE-coated oven-dried stir bar was added dichloromethane (72.5 mL) and dimethylsulfoxide (.659 mL, 9.29 mmol, 1.5 equiv). The flask was purged with N_2 for five minutes and cooled to -78°C (dry ice/acetone). Oxalyl chloride (.813 mL, 9.29 mmol, 1.5 equiv) was added and the solution was stirred for five minutes. The remaining 10 mL of dichloromethane (82.5 mL total, 75 mM) was used to solubilize corresponding hydroxyoxazoline (6.19 mmol, 1 equiv) and the solution was slowly added to the reaction mixture. The contents of the flask were stirred at -78°C for three hours. Triethylamine (2.59 mL, 18.6 mmol, 3 equiv) was added and stirred for 30 minutes. The flask was warmed to room temperature. During this time, the solution changed color from clear to opaque white to translucent yellow. The reaction was quenched with 40 mL deionized water and extracted 3 x 30 mL with dichloromethane. The organic layers were combined, dried with Na_2SO_4, and filtered, into a flask containing 1,3,5-trimethoxybenzene as an internal standard. An aliquot was removed from the homogenous solution and dried. An NMR yield was collected (~75% yield). The bulk solution was concentrated *in vacuo* to give a yellow oil. The crude product was used immediately with no further purification.*
*Note: ketone intermediate is instable and decomposes at room temperature over the course of 8 hours (see section 6).

(*S*)-1-(4-(*tert*-butyl)-4,5-dihydrooxazol-2-yl)ethan-1-one **(3)**

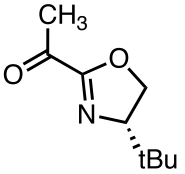

The title compound was synthesized according to General Procedure B and collected as a crude yellow oil (820 mg, 78% NMR yield).
^{1}H NMR (400 MHz, Chloroform-*d*): δ 4.34 (dd, *J* = 10.4, 8.8, 1.1 Hz 1H), 4.20 (t, *J* = 8.8, 1.2 Hz 1H), 4.08 (dd, *J* = 10.2, 8.7, 1.2 Hz, 1H), 2.53 (s, 3H), 0.93 (s, 9H).
^{13}C{^{1}H} NMR (101 MHz, Chloroform-*d*): δ 190.9, 159.8, 69.7, 53.3, 34.0, 27.1, 25.7.
HRMS (ESI) m/z: $[M + H]^+$ Calculated for $C_8H_{14}NO_2^+$ 170.1181; Found 170.1168.
FT-IR: (cm^{-1}, neat, ATR) ṽ **=** 2958.04, 1717.15, 1634.52, 1118.15, 975.19.

(*S*)-1-(4-isopropyl-4,5-dihydrooxazol-2-yl)ethan-1-one **(4)**

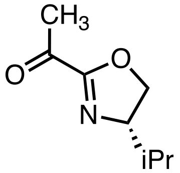

The title compound was synthesized according to General Procedure B and collected as a crude yellow oil (710 mg, 74% NMR yield).
^{1}H NMR (400 MHz, Chloroform-*d*): δ 4.44 – 4.33 (m, 1H), 4.16 – 4.07 (m, 2H), 2.52 (s, 3H), 1.85 (m, 1H), 1.01 (d, *J* = 6.7 Hz, 3H), 0.91 (d, *J* = 6.8 Hz, 3H).
^{13}C{^{1}H} NMR (101 MHz, Chloroform-*d*): δ 190.8, 159.9, 73.2, 71.1, 32.6, 27.4, 18.9, 18.4.
HRMS (ESI) m/z: $[M + H]^+$ Calculated for $C_8H_{14}NO_2^+$ 156.102; Found 156.0987.
FT-IR: (cm^{-1}, neat, ATR) ṽ **=** 2963.38, 1733.73, 1635.69, 1117.55, 1024.08.

General Procedure C:

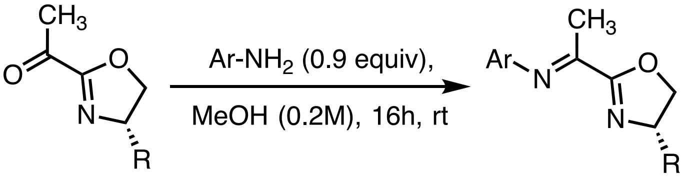

1 equiv

R = *t*Bu: Ar = Mes: 32% yield; Ar = Dipp: 46% yield
R = *i*Pr: Ar = Mes: 35% yield; Ar = Dipp: 58% yield

To the flask containing the crude ketone was added methanol (0.20 M) and a PTFE-coated stir bar. The corresponding aniline derivative (6.20 mmol, 0.9 equiv) was added dropwise over one hour. The homogenous yellow/orange solution was stirred at room temperature for 16 hours. Reaction progress was monitored by TLC or GCMS. Solvent was removed *in vacuo* and the crude orange/red oil was purified by column chromatography.

^{Mes}Im^{iPr}Ox **(6)**

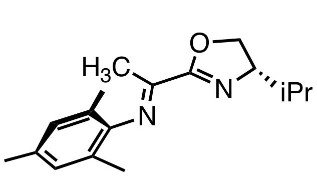

The title compound synthesized according to General Procedure C and purified by column chromatography on silica eluting with 4:1 hexanes : ethyl acetate (R_f = 0.24). Product collected as an off-white/yellow solid. 200.0 mg, 35% yield.
^{1}H NMR (400 MHz, Chloroform-*d*): δ 6.83 (s, 2H), 4.47 (dd, *J* = 9.4, 8.0 Hz, 1H)*, 4.18 (t, 1H), 4.12 (dd, 1H)*, 2.25 (s, 3H), 1.96 (s, 3H), 1.98 (s, 6H), 1.93 – 1.81 (m, 1H)*, 1.05 (d, *J* = 6.8 Hz, 3H), 0.95 (d, *J* = 6.7 Hz, 3H). *Overlapping signal, so J-coupling could not be reported.
^{13}C{^{1}H} NMR (101 MHz, Chloroform-*d*): δ 162.6, 159.1, 145.2, 132.8, 128.7, 128.6, 124.8, 124.6, 73.0, 70.9, 32.9, 20.8, 19.1, 18.3, 17.9, 17.88, 17.85.
HRMS (ESI) m/z: [M + H]$^+$ Calculated for $C_{17}H_{25}N_2O^+$ 273.1967. Found 273.1968.
FT-IR: (cm^{-1}, neat, ATR) ṽ **=** 3372.93, 2961.17, 1646.57, 1523.09, 1217.22.

^{Dipp}Im^{iPr}Ox **(7)**

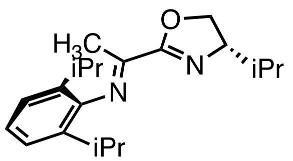

The title compound synthesized according to General Procedure C and purified by column chromatography on silica eluting with 10:1 hexanes : ethyl acetate (R_f = 0.16). Product collected as a yellow oil. 381.20 mg, 58% yield.
^{1}H NMR (400 MHz, Chloroform-*d*): δ 7.15 – 7.05 (m, 3H), 4.49 (dd, *J* = 9.4, 8.1 Hz, 1H), 4.20 (t, 1H)*, 4.14 (dd, 1H)*, 2.68 (hept, *J* = 6.9 Hz, 2H), 2.02 (s, 3H), 1.91 (hept, *J* = 6.5 Hz 1H), 1.18 – 1.09 (m, 12H), 1.06 (d, *J* = 6.8 Hz, 3H), 0.96 (d, *J* = 6.7 Hz, 3H). *Overlapping signal, so J-coupling could not be reported.
^{13}C{^{1}H} NMR (101 MHz, Chloroform-*d*): δ 162.9, 159.5, 145.5, 136.0, 136.0, 124.8, 123.5, 123.5, 73.4, 71.4, 33.2, 28.6, 28.5, 23.9, 23.8, 23.7, 23.6, 19.4, 19.0, 18.6.
HRMS (ESI) m/z: [M + H]$^+$ Calculated for $C_{20}H_{31}N_2O^+$ 315.2436; Found 315.2425.
FT-IR: (cm^{-1}, neat, ATR) ṽ **=** 2959.65, 1628.66, 1359.96, 1116.64.

$^{Mes}Im^{tBu}Ox$ **(8)**

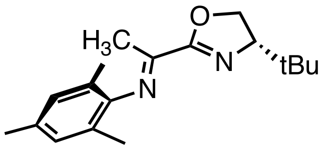

The title compound synthesized according to General Procedure C and purified by column chromatography on silica eluting with 10:1 hexanes : ethyl acetate (R_f = 0.20). Product collected as an off-white/yellow solid. 161 mg, 32% yield.

^{1}H NMR (400 MHz, Chloroform-*d*): δ 6.84 (s, 2H), 4.41 (dd, *J* = 10.3, 8.8 Hz, 1H), 4.28 (t, *J* = 8.5 Hz, 1H), 4.08 (dd, *J* = 10.3, 8.2 Hz, 1H), 2.26 (s, 3H), 1.97 (s, 3H), 1.98 (s, 6H), 0.97 (s, 9H).

^{13}C{^{1}H} NMR (101 MHz, Chloroform-*d*): δ 162.5, 159.2, 145.3, 132.9, 128.68, 128.66, 124.8, 124.7, 76.6, 69.5, 34.0, 26.0, 20.9, 17.91, 17.88, 17.83.

HRMS (ESI) m/z: $[M + H]^+$ Calculated for $C_{18}H_{27}N_2O^+$ 287.2123; Found 287.2163.

FT-IR: (cm^{-1}, neat, ATR) ṽ **=** 3376.41, 2955.23, 1627.98, 1474.27, 1358.09, 1117.38

$^{Dipp}Im^{tBu}Ox$ **(9)**

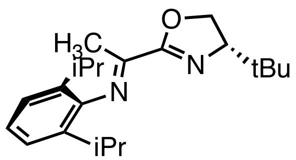

The title compound synthesized according to General Procedure C and purified by column chromatography on silica eluting with 10:1 hexanes : ethyl acetate (R_f = 0.23). Product collected as an off-white/yellow solid. 314.40 mg, 46% yield.

^{1}H NMR (400 MHz, Chloroform-*d*): δ 7.15 – 7.07 (m, 3H), 4.42 (dd, *J* = 10.3, 8.9 Hz, 1H), 4.30 (t, *J* = 8.9, 8.0 Hz, 1H), 4.10 (dd, *J* = 10.3, 8.1 Hz, 1H), 2.68 (hept, *J* = 6.8, 1.1 Hz, 2H), 2.02 (s, 3H), 1.16 – 1.10 (m, 12H), 0.98 (s, 9H).

^{13}C{^{1}H} NMR (101 MHz, Chloroform-*d*): δ 162.5, 159.2, 145.2, 135.7, 135.7, 124.4, 123.2, 123.1, 76.5, 69.5, 34.1, 28.4, 28.2, 26.0, 23.6, 23.4, 23.30, 23.27, 18.7.

HRMS (ESI) m/z: $[M + H]^+$ Calculated for $C_{21}H_{33}N_2O^+$ 329.2593; Found [M+H]: 329.2471.

FT-IR: (cm^{-1}, neat, ATR) ṽ **=** 3379.82, 2958.91, 1629.96, 1361.93, 1119.42.

3. Enantioselective Conjugate Addition of Boronic Acids to 3-Substituted Cyclohexenone

3.1 General Procedure D:

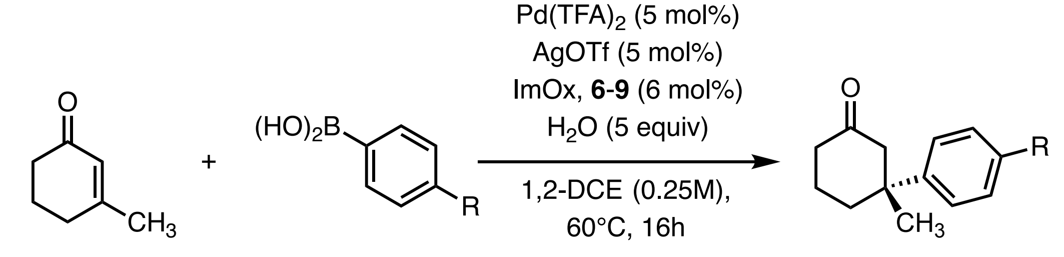

1 equiv, 0.250 mmol 2 equiv

This reaction was performed in analogy to previous reported conditions.[7, 8] No additional measures were taken to exclude air or moisture. To a 4 mL vial containing a magnetic stir bar was added cyclohexenone substrate (0.250 mmol, 1 equiv), $Pd(TFA)_2$ (4.2 mg, 0.013 mmol, 0.05 equiv), ImOx (0.015 mmol, 0.06 equiv), AgOTf (3.2 mg, 0.013 mmol, 0.05 equiv), boronic acid (0.500 mmol, 2 equiv), and deionized water (22.5 mg, 1.25 mmol, 5 equiv). 1,2-dichloroethane (1.0 mL, 0.25 mM) was added. The vial was capped with a Teflon/silicone septum and heated to 60°C at once using a ChemGlass Reaction Block (15 mm x 45 mm, 19 mm hole depth), stirring at 900 rpm for 16 hours. For NMR yields, the crude reaction mixture was filtered into a vial containing 1,3,5-trimethoxybenzene through a plug of celite, washing 3 x 3 mL DCM. An aliquot was removed, dried *in vacuo*, and suspended in 500 µL Chloroform-d. For isolated yields, the crude reaction mixture was directly subjected to column chromatography to afford the desired product.

Racemic products were synthesized using the procedure above using 2,2'-bipyridine (2.4 mg, 0.015 mmol, 0.06 equiv) as an achiral ligand or a reported procedure from Stanley *et. al.*[9]

3.2 General Procedure E:

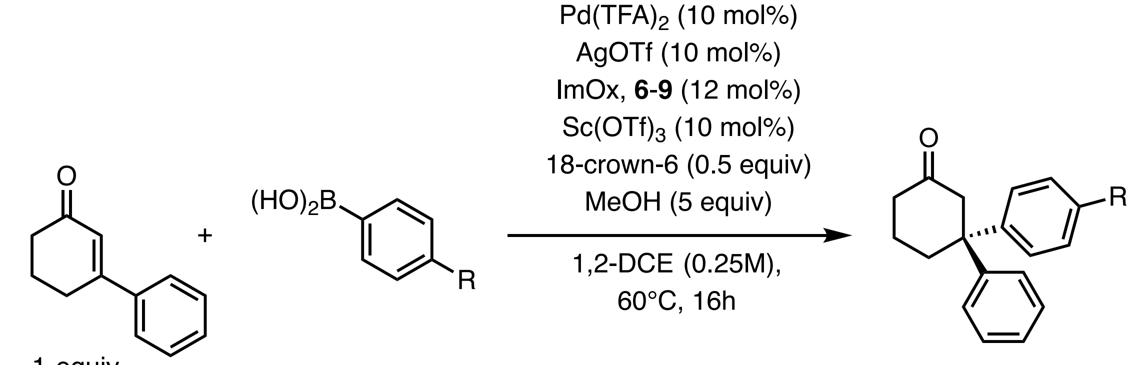

1 equiv, 0.250 mmol 3 equiv, portionwise

This reaction was performed in analogy to previous reported conditions.[10] To a 4 mL vial containing a magnetic stir bar was added 3-phenylcyclohexenone (43.1 mg, 0.250 mmol, 1 equiv), $Pd(TFA)_2$ (8.3 mg, 0.025 mmol, 0.10 equiv), ImOx (0.030 mmol, 0.12 equiv), $Sc(OTf)_3$ (13.1 mg, 0.030 mmol, 0.10 equiv), AgOTf (7.7 mg, 0.025 mmol, 0.10 equiv), boronic acid (0.250 mmol, 1 equiv), 18-crown-6 (33.0 mg, 0.125 mmol, 0.50 equiv) and methanol (81.4 µL, 1.25 mmol, 5 equiv). 1,2-dichloroethane (1.0 mL, .25 mM) was added. The vial was capped with a Teflon/silicone septum and heated to 60°C at once using a ChemGlass Reaction Block (15 mm x

45 mm, 19 mm hole depth), stirring at 900 rpm for three hours. The reaction was removed from the heat and a second equivalent (0.250 mmol) of boronic acid was added. The vial was capped and continued heating at 60°C for three hours. The reaction was removed from the heat and a third equivalent (0.250 mmol) of boronic acid was added. The reaction was then stirred overnight at 60°C for seven hours. For NMR yields, the crude reaction mixture was filtered through a plug of celite (washing 3 x 3 mL DCM) into a vial containing 1,3,5-trimethoxybenzene. An aliquot was removed, dried *in vacuo*, and suspended in 500 µL of chloroform-d. For isolated yields, the crude reaction mixture was directly subjected to column chromatography (100% DCM, R_F = 0.4 or 100% hexanes to 10% EtOAc in hexanes R_F = 0.2) to afford the product as a white solid.

Racemic products were synthesized according to the procedure above using 2,2'-bipyridine (4.69 mg, .030 mmol, 0.10 equiv) as an achiral ligand, omitting $Sc(OTf)_3$, 18-crown-6, and replacing methanol with deionized water (22.5 µL, 1.25 mmol, 5 equiv) or a reported procedure from Stanley *et. al.*[9]

3.3 Characterization of Products

(*R*)-3-methyl-3-phenylcyclohexan-1-one (10)

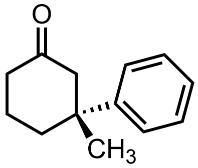

10

The title compound was synthesized following General Procedure D from 3-methylcyclohexenone and phenylboronic acid. Purification by flash chromatography (100% DCM, R_F = 0.4) afforded the product as a colorless oil (45.2 mg, 0.240 mmol, 96% yield). The enantiomeric excess was determined by HPLC analysis (210 nm, 25°C), t_R 11.7 min (major); t_R 14.7 min (minor) [Chiralpak OJ-H (4.6 mm I.D. x 250 mm L) hexane/*i*PrOH, 99:1, 1.0 mL/min] to be >99% ee. $[\alpha]_D^{23}$ = -60.3 (*c* 0.33, CH_2Cl_2). All characterization data was consistent with previous reports.[7, 11, 12, 13, 14]

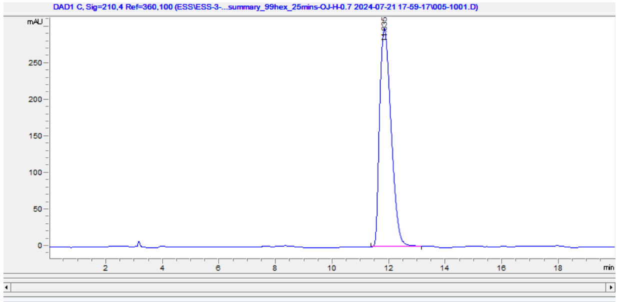

#	Time	Area	Height	Width	Area%	Symmetry
1	11.835	8675.9	298.1	0.4635	100.000	0.654

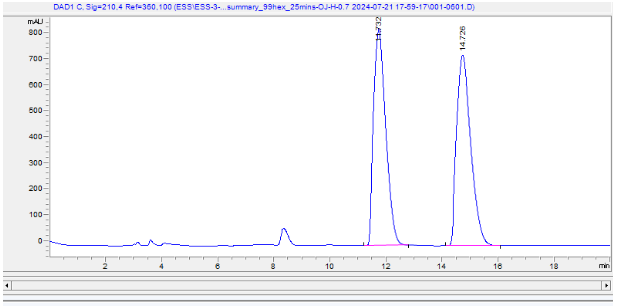

#	Time	Area	Height	Width	Area%	Symmetry
1	11.732	25747.4	833.1	0.4946	50.024	0.714
2	14.726	25722.7	731.4	0.5603	49.976	0.687

(*R*)-3-(4-methoxyphenyl)-3-methylcyclohexan-1-one (11)

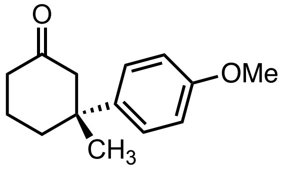

11

The title compound was synthesized following General Procedure D from 3-methylcyclohexenone and 4-methoxyphenylboronic acid. Purification by flash chromatography (100% hexanes to 10% EtOAc in hexanes, R_F = 0.25) afforded the product as a clear yellow oil (32.8 mg, 0.150 mmol, 59% yield). The enantiomeric excess was determined by HPLC analysis (210 nm, 25°C), t_R 26.5 min (major); t_R 31.3 min (minor) [Chiralpak OJ-H (4.6 mm I.D. x 250 mm L) hexane/*i*PrOH, 99:1, 1.0 mL/min] to be 92% ee. $[\alpha]_D^{23}$ = -6.28 (*c* 0.0022, CH_2Cl_2). All characterization data was consistent with previous reports.[7, 13]

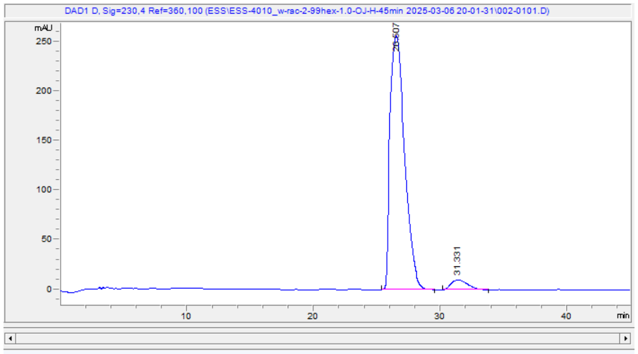

#	Time	Area	Height	Width	Area%	Symmetry
1	26.507	20510.1	255.7	1.2238	95.868	0.688
2	31.331	883.9	9.8	1.0695	4.132	0.606

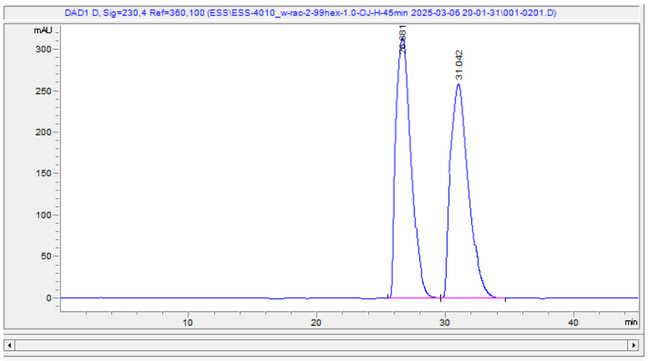

#	Time	Area	Height	Width	Area%	Symmetry
1	26.681	25470.7	313.2	1.2808	49.970	0.748
2	31.042	25501.4	257.8	1.36	50.030	0.798

(*R*)-3-phenyl-3-(*p*-tolyl)cyclohexan-1-one (12)

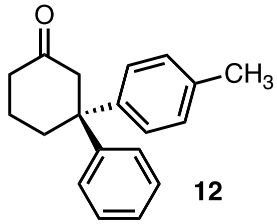

12

The title compound was synthesized following General Procedure E from 3-phenylcyclohexenone and *para*-tolylboronic acid. Purification by flash chromatography (100% hexanes to 10% EtOAc in hexanes, R_F = 0.25) afforded the product as a white solid (57.5 mg, 0.218 mmol, 87% yield). The enantiomeric excess was determined by HPLC analysis (220 nm, 25°C), t_R 29.0 min (major); t_R 31.2 min (minor) [Chiralpak IA-3 (4.6 mm I.D. x 250 mm L) hexane/*i*PrOH, 99:1, 0.3 mL/min] to be 98% ee. $[\alpha]_D^{23}$ = -2.02 (*c* 1.08, CH_2Cl_2). All characterization data was consistent with previous reports.[9, 10]

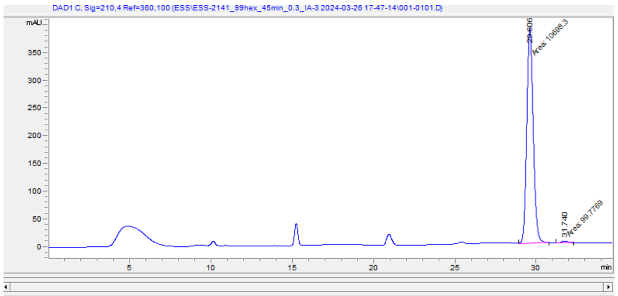

#	Time	Area	Height	Width	Area%	Symmetry
1	29.606	10698.3	385.9	0.462	99.076	0.856
2	31.74	99.8	3.3	0.4968	0.924	0.978

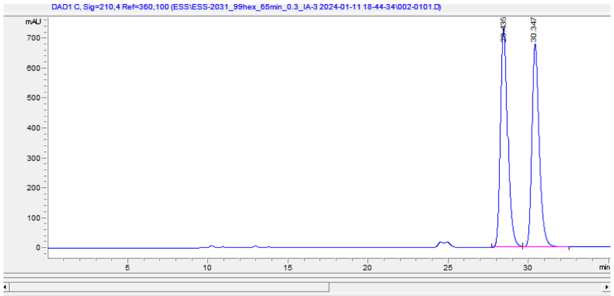

#	Time	Area	Height	Width	Area%	Symmetry
1	28.435	21458.4	730.6	0.4467	49.862	0.75
2	30.347	21577.3	681.7	0.4813	50.138	0.725

(*R*)-3-phenyl-3-(*p*-methoxy)cyclohexan-1-one (13)

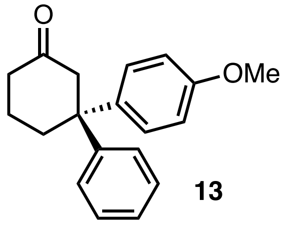

13

The title compound was synthesized following General Procedure E from 3-phenylcyclohexenone and *para*-methoxyboronic acid. Purification by flash chromatography (100% hexanes to 10% EtOAc in hexanes, R_F = 0.30) afforded the product as a white solid (27.3 mg, 0.974 mmol, 39% yield). The enantiomeric excess was determined by HPLC analysis (230 nm, 25°C), t_R 22.4 min (major); t_R 27.0 min (minor) [Chiralpak IA-3 (4.6 mm I.D. x 250 mm L) hexane/*i*PrOH, 99:1, 0.5 mL/min] to be 94% ee. $[\alpha]_D^{23}$ = -5.27 (*c* 0.0014, CH_2Cl_2). All characterization data was consistent with previous reports.[9, 10]

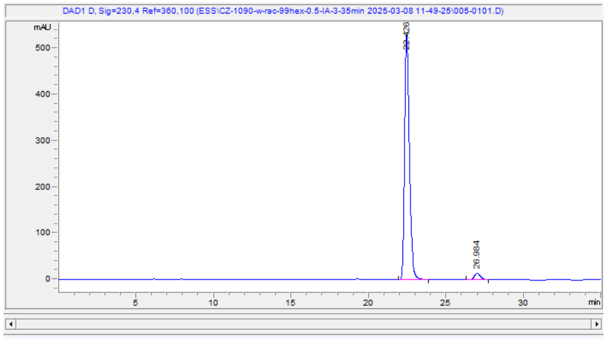

#	Time	Area	Height	Width	Area%	Symmetry
1	22.426	11220.9	529.9	0.321	96.854	0.673
2	26.984	364.5	13.8	0.3974	3.146	0.876

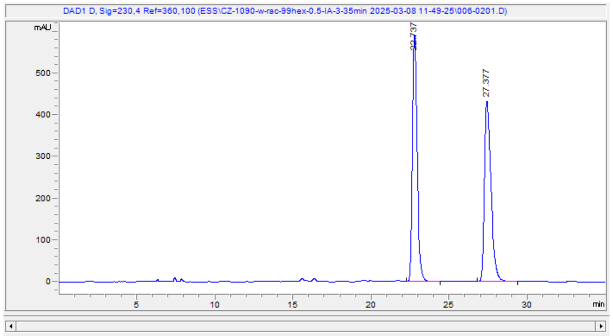

#	Time	Area	Height	Width	Area%	Symmetry
1	22.737	12639.6	590.2	0.3238	49.909	0.668
2	27.377	12685.5	432.3	0.4403	50.091	0.524

3.3 Ligand Effect Summary for the Conjugate Addition to 3-Methylcyclohexenone and Chiral Assays

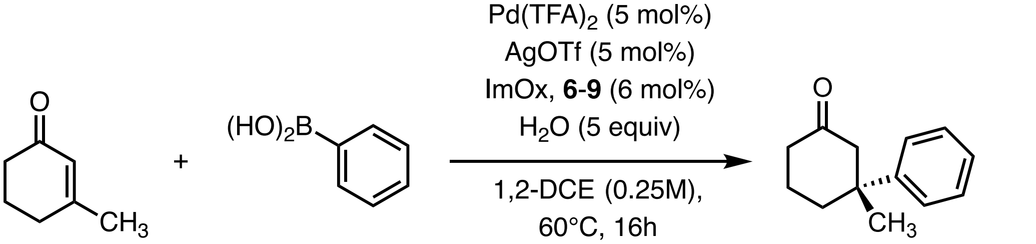

1 equiv, 0.250 mmol

2 equiv

10
up to 96% yield, >99% *ee*

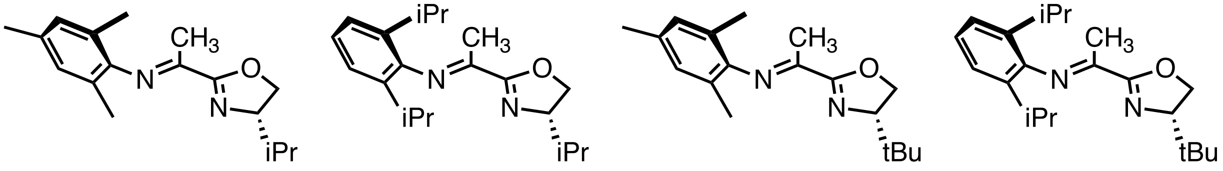

(S)-^{Mes}Im^{iPr}Ox, **6** *(S)*-^{Dipp}Im^{iPr}Ox, **7** *(S)*-^{Mes}Im^{tBu}Ox, **8** *(S)*-^{Dipp}Im^{tBu}Ox, **9**

Entry	ImOx Derivative	Isolated Yield (NMR Yield)	*ee*
1	**6**	90%	62%
2	**7**	86%	90%
3	**8**	89% (94%)	94%
4	**9**	(96%)	>99%

Table S1. Ligand effect summary for the Pd-catalyzed conjugate addition of phenylboronic acid to 3-methylcyclohexenone.

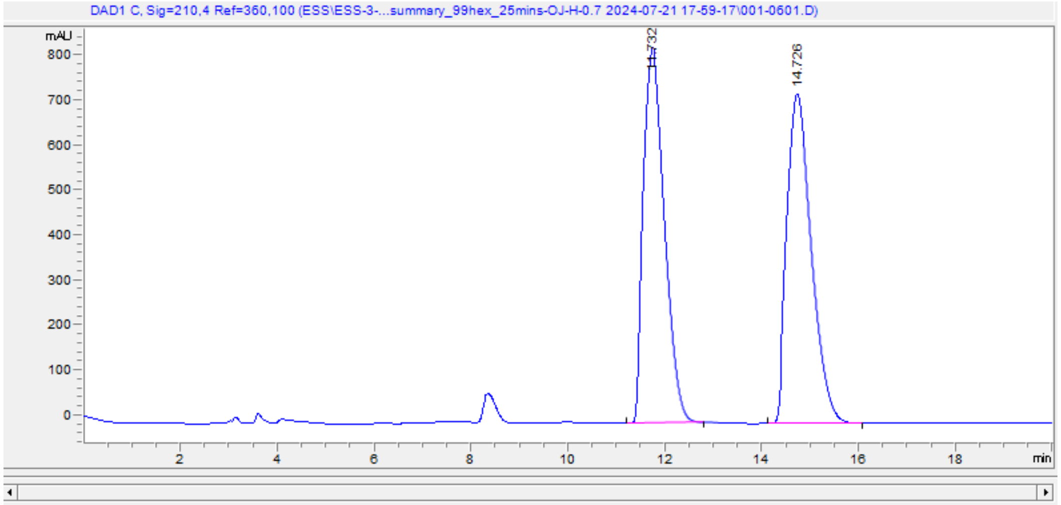

#	Time	Area	Height	Width	Area%	Symmetry
1	11.732	25747.4	833.1	0.4946	50.024	0.714
2	14.726	25722.7	731.4	0.5603	49.976	0.687

Figure S1. HPLC trace of racemic **10**.

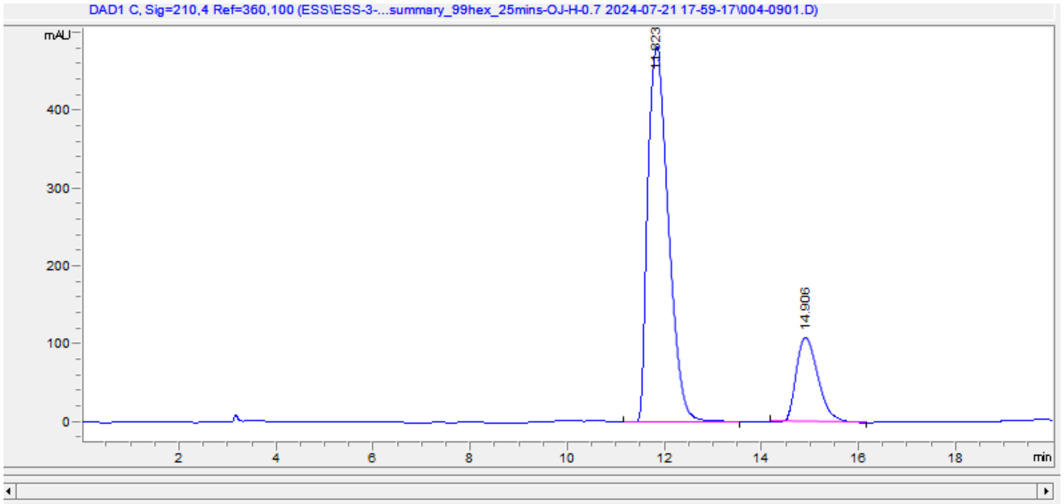

#	Time	Area	Height	Width	Area%	Symmetry
1	11.823	14644.4	482.2	0.4843	81.056	0.675
2	14.906	3422.6	108.7	0.4972	18.944	0.702

Figure S2. HPLC trace of **10** using **6** as the chiral ligand (62% ee).

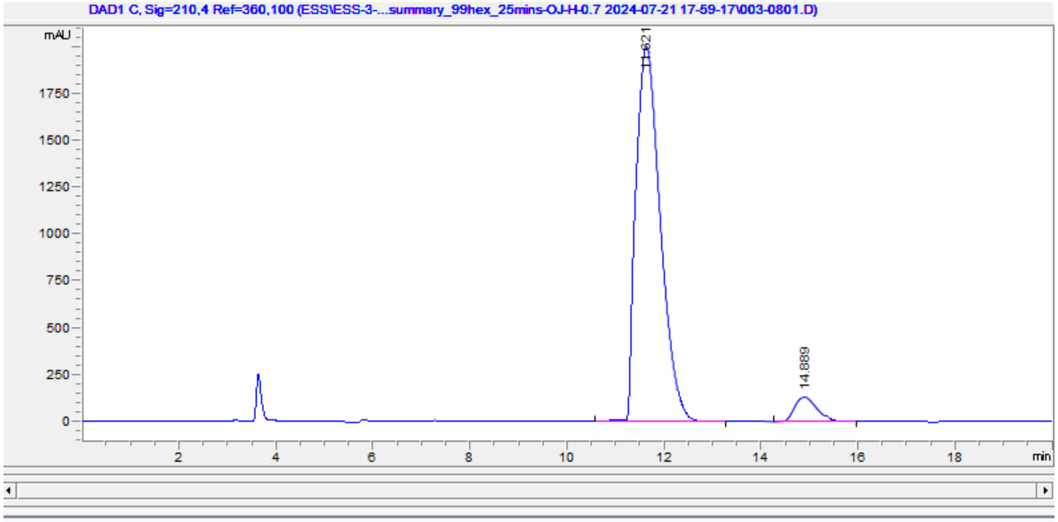

#	Time	Area	Height	Width	Area%	Symmetry
1	11.621	70635.6	1999.6	0.5691	94.488	0.679
2	14.889	4120.2	130.8	0.4936	5.512	0.719

Figure S3. HPLC trace of **10** using **7** as the chiral ligand (90% ee).

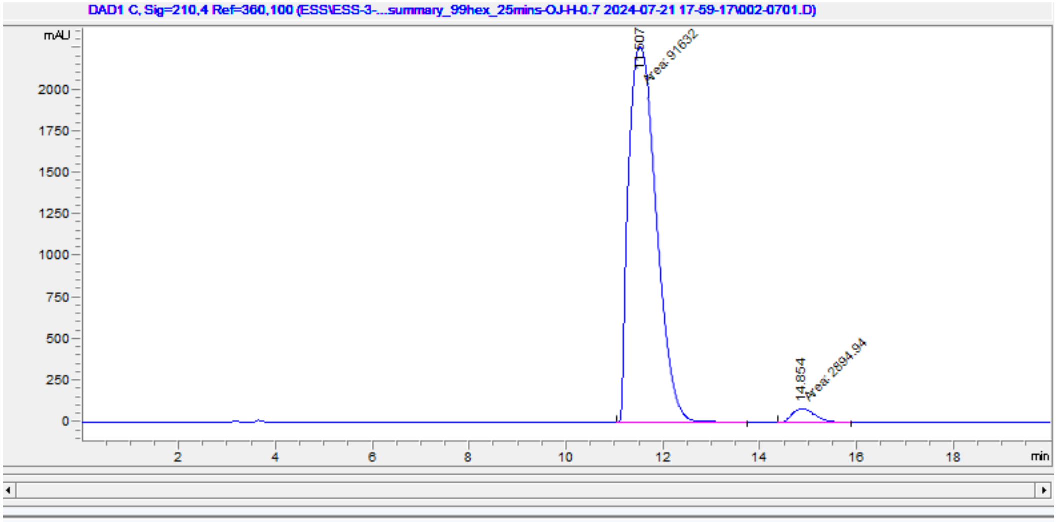

#	Time	Area	Height	Width	Area%	Symmetry
1	11.507	91632	2265.2	0.6742	96.937	0.614
2	14.854	2894.9	85.2	0.566	3.063	0.677

Figure S4. HPLC trace of **10** using **8** as the chiral ligand (94% ee).

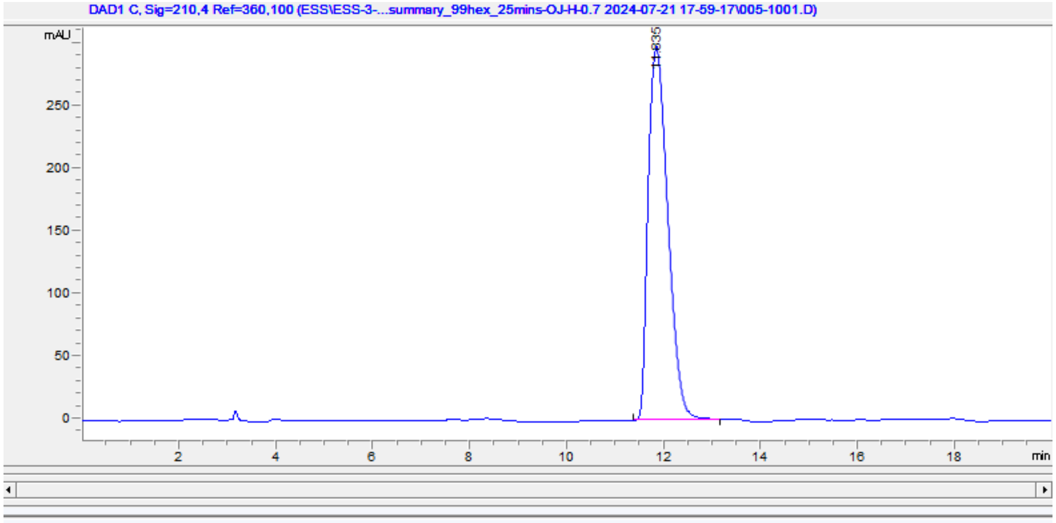

#	Time	Area	Height	Width	Area%	Symmetry
1	11.835	8675.9	298.1	0.4635	100.000	0.654

Figure S5. HPLC trace of **10** using **9** as the chiral ligand (>99% ee).

3.4 Conjugate Addition to 3-Methylcyclohexenone Reaction Optimization

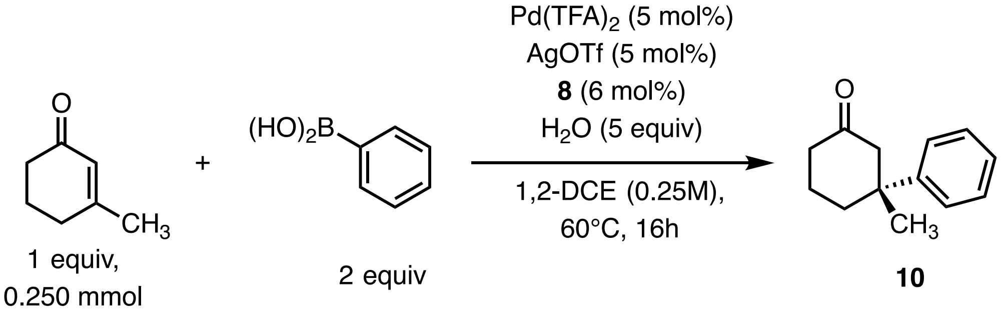

Entry	Deviation from Standard Conditions	Isolated Yield	*ee*
1	None	24%	93%
2	No AgOTf	10%[a]	n.d.
3	8 mol% AgOTf	32%	79%
4	10 mol% AgOTf	34%	75%
5	15 mol% AgOTf	51%	63%
6	70°C	20%	98%
7	50°C	27%	n.d.
8	Double concentration (.50M)	25%[b]	n.d.
9	Half concentration (.125M)	36%[b]	n.d.
10	10 mol% $Pd(OTf)_2$, 10 mol% AgOTf. 12 mol%	51%[b]	n.d.
11	wet $PhB(OH)_2$	55%	93%
12	5 equiv H_2O	89%	96%

Table S2. Optimization of the Pd-catalyzed conjugate addition to 3-methylcyclohexenone. [a]NMR yield reported using 1,3,5-trimethoxybenzene as an internal standard; [b]GC yield reported using tetradecane and an internal standard.

Replacing ^{tBu}PyOx with ImOx in the Pd-catalyzed asymmetric conjugate addition to α,β-unsaturated enones with no other deviation from standard conditions showed trace amount of **10**, but a significant amount of biphenyl . This indicated that an additional transmetallation was occurring to form biphenyl and reductively eliminating. To suppress this off cycle pathway, AgOTf was added to form a cationic Pd^{II} species through counterion abstraction.[15, 16] The purpose of this additive was to increase the affinity for enone binding by increasing the eletrophilicity of the metal center as well as suppress unproductive transmetalation pathways to form biphenyl. In the presence of 5 mol% AgOTf, the conjugate addition gave low yields, but a promising enantioselectivity of 93% ee (entry 1), while the absence of AgOTf gave a 10% NMR yield (entry 2). Increasing the loading of AgOTf showed a direct relationship to product yield and an inverse relationship to the ee (entries 3-5). This result suggests that there may be a competing pathway to form the desired product mediated by Ag(I) in the absence of a chiral ligand. Changing the temperature of the reaction from 60°C to 70°C or 50°C did not impact the product yield, but did show increased ee at elevated temperatures (entries 6 and 7). Making the reaction slightly more dilute at a concentration of 0.125 molar marginally increased the yield, which suggests that a lower concentration of the boronic acid will prevent the undesired second

transmetalation/reductive elimination pathway (entries 8 and 9). Doubling the catalyst loading boosted the yield to 51% (entry 10), but was not pursued because of the concurrent discovery that non-dried phenylboronic acid gave a higher yield and ee at the original catalyst loading (entry 11). Thorough studies have been conducted to demonstrate the role of a proton source in catalyst turn over for this reaction.[7, 10, 12] The stoichiometric addition of water drastically improved the yield and ee, ultimately delivering the desired conjugate addition product in an 89% yield with 96% ee (entry 12).

3.5 Ligand Effect Summary for the Conjugate Addition to 3-Phenylcyclohexenone and Chiral Assays

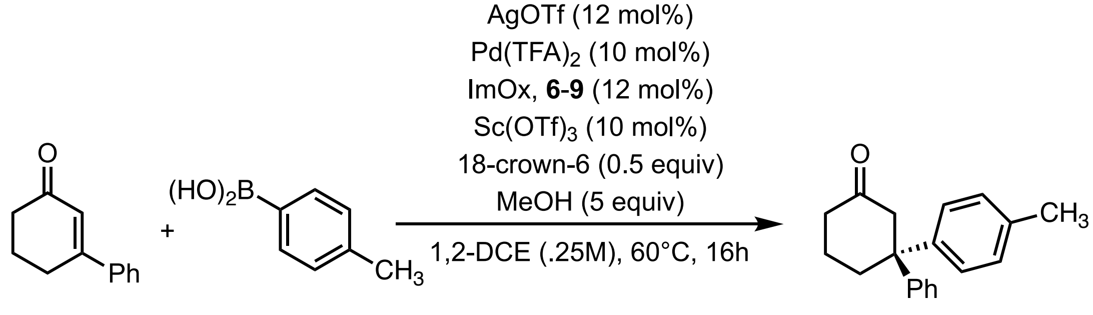

1 equiv
0.102 mmol

3 equiv, portionwise

12
up to 87% yield, 98% ee

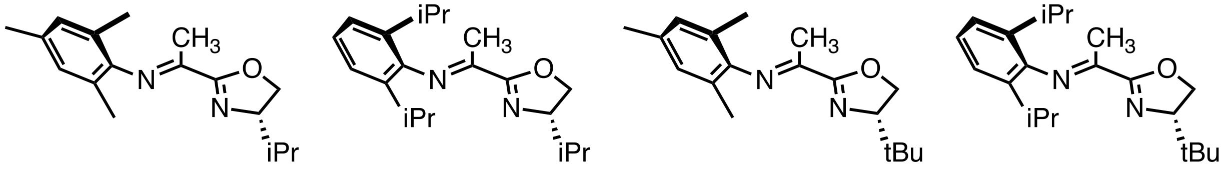

(S)-^{Mes}Im^{iPr}Ox, **6** *(S)*-^{Dipp}Im^{iPr}Ox, **7** *(S)*-^{Mes}Im^{tBu}Ox, **8** *(S)*-^{Dipp}Im^{tBu}Ox, **9**

Entry	ImOx Derivative	NMR Yield (Isolated Yield)	*ee*
1	**6**	62% (53%)	66%
2	**7**	66% (52%)	88%
3	**8**	72% (72%)	94%
4	**9**	(87%)	98%

Table S3. Ligand effect summary for the Pd-catalyzed conjugate addition of *para*-tolylboronic acid to 3-phenylcyclohexenone.

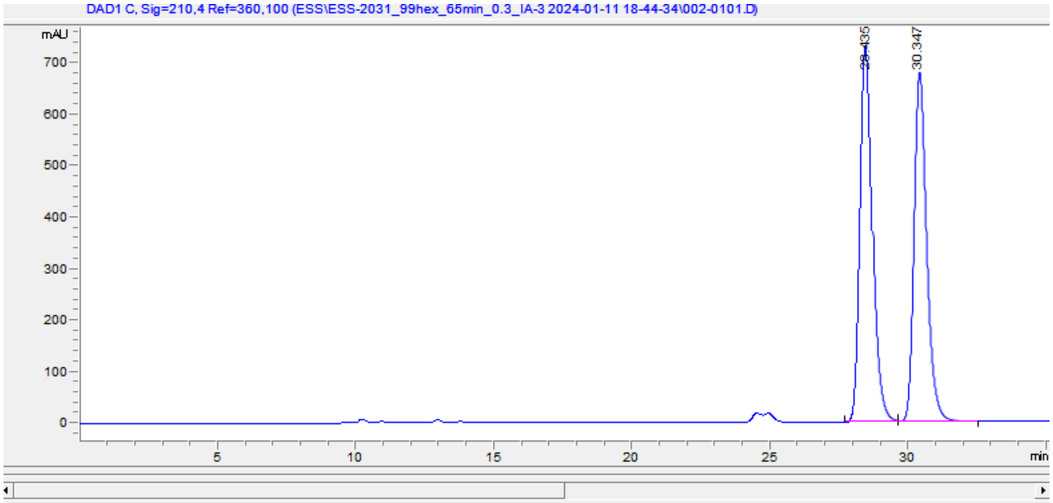

#	Time	Area	Height	Width	Area%	Symmetry
1	28.435	21458.4	730.6	0.4467	49.862	0.75
2	30.347	21577.3	681.7	0.4813	50.138	0.725

Figure S6. HPLC trace of racemic **12**.

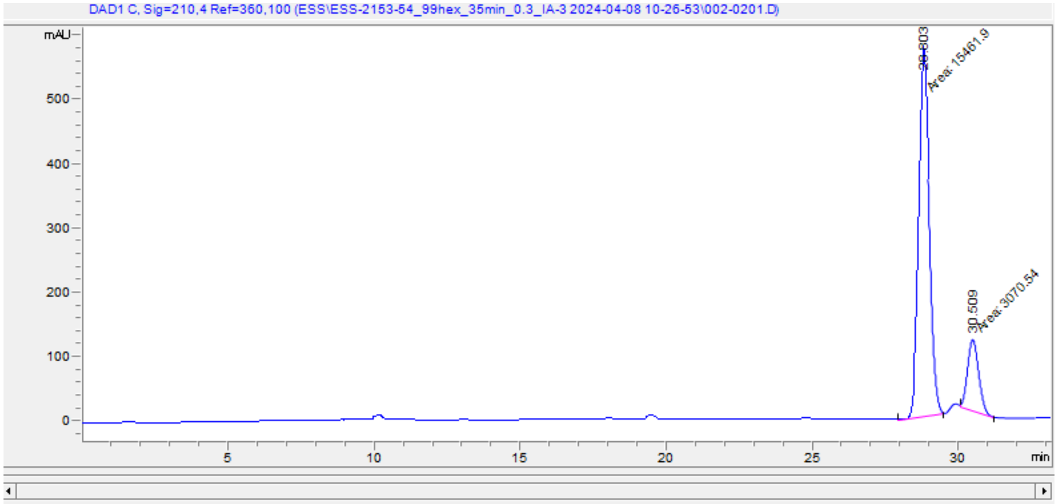

#	Time	Area	Height	Width	Area%	Symmetry
1	28.803	15461.9	576.2	0.4472	83.432	0.862
2	30.509	3070.5	112.8	0.4537	16.568	0.825

Figure S7. HPLC trace of **12** using **6** as the chiral ligand (66% ee).

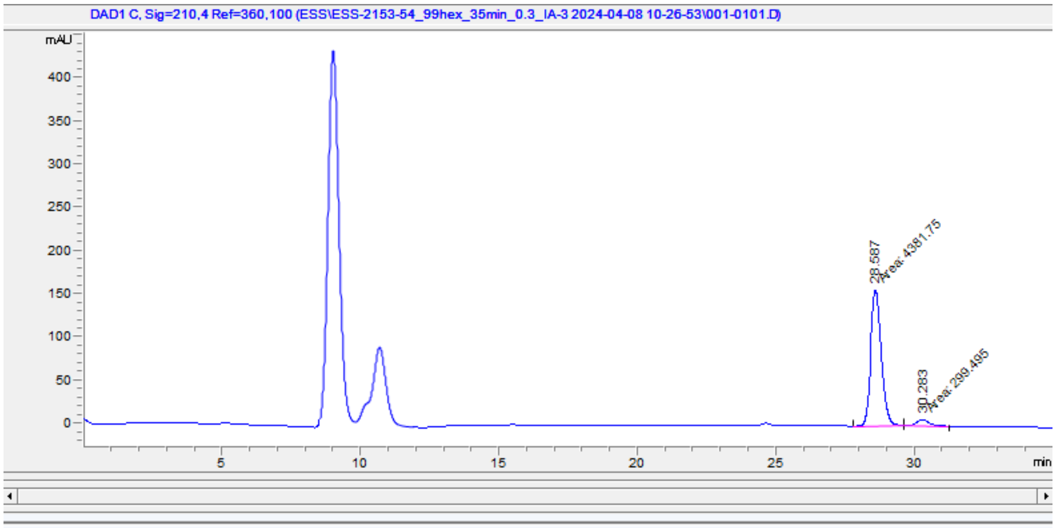

#	Time	Area	Height	Width	Area%	Symmetry
1	28.587	4381.7	158.5	0.4608	93.602	0.875
2	30.283	299.5	7.6	0.6547	6.398	0.702

Figure S8. HPLC trace of **12** using **7** as the chiral ligand (88% ee).

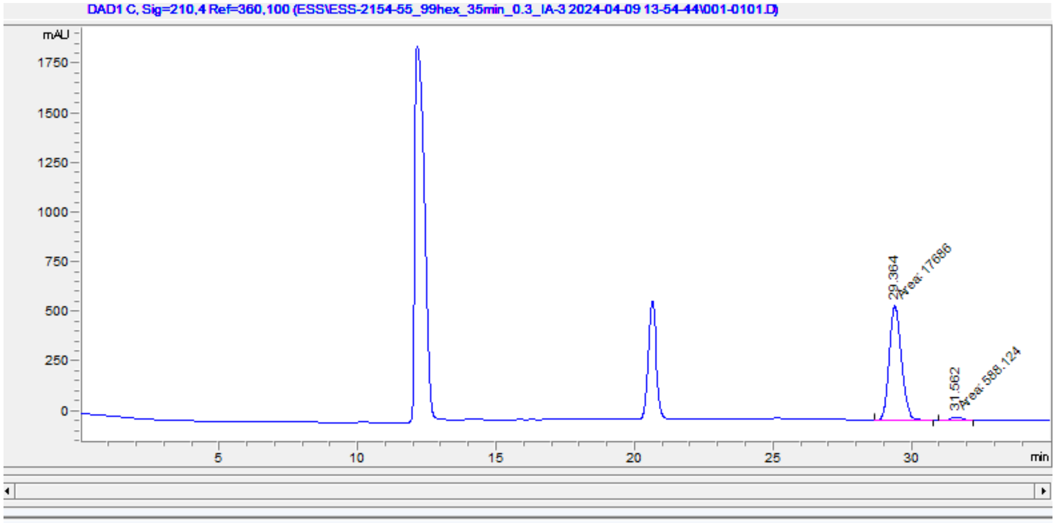

#	Time	Area	Height	Width	Area%	Symmetry
1	29.364	17686	579.8	0.5084	96.782	0.814
2	31.562	588.1	18.7	0.5243	3.218	0.849

Figure S9. HPLC trace of **12** using **8** as the chiral ligand (94% ee).

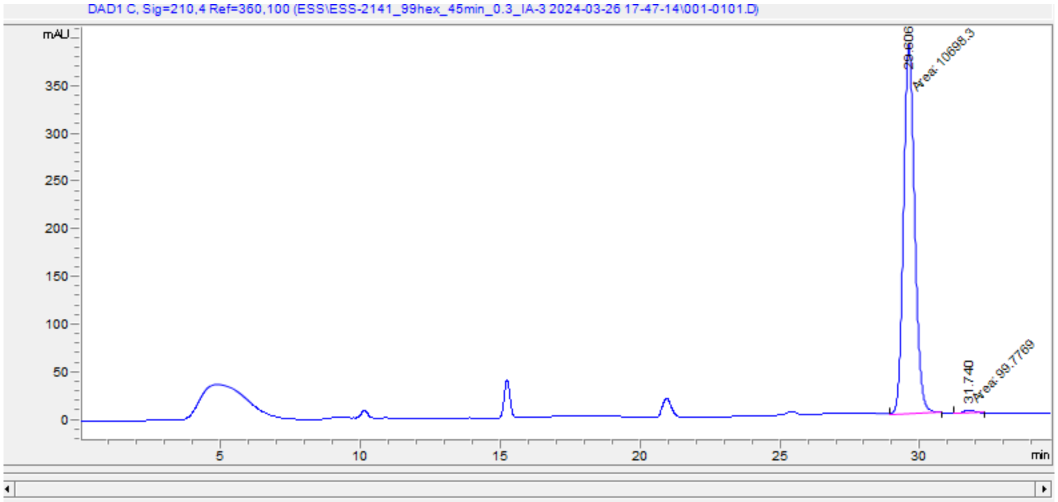

#	Time	Area	Height	Width	Area%	Symmetry
1	29.606	10698.3	385.9	0.462	99.076	0.856
2	31.74	99.8	3.3	0.4968	0.924	0.978

Figure S10. HPLC trace of **12** using **9** as the chiral ligand (98% ee).

3.6 Conjugate addition to 3-phenylcyclohexenone reaction optimization

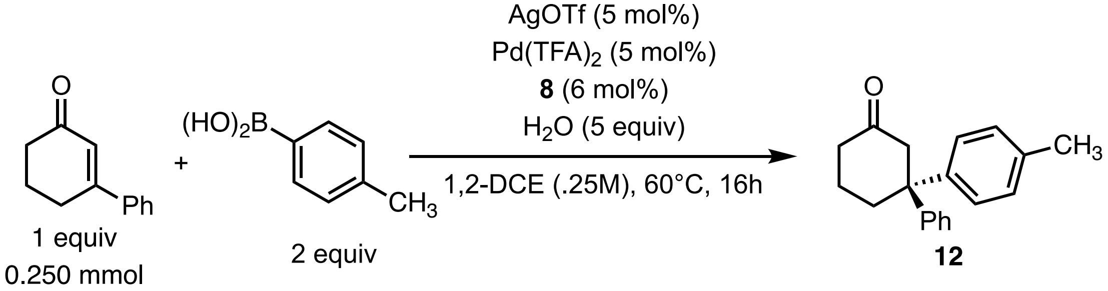

Entry	Deviation from Standard Conditions	NMR Yield (Isolated Yield)	*ee*
1	None	(10%)	92%
2	No AgOTf	trace	n.d.
3	80°C	16 (5%)	86%
4	4 equiv boronic acid	24% (18%)	90%
5	10 mol% Pd(TFA)$_2$, 10 mol% AgOTf, 12 mol% ligand	29%	88%
6	0.5 equiv TBABr	n.d.	n.d.
7	0.5 equiv 18-crown-6	12% (7%)	96%
8	Portionwise addition of boronic acid (3 equiv)	32 (26%)	92%
9	Portionwise addition, 0.5 equiv 18-c-6, double catalyst loading	48%	94%

Table S4. First optimization iteration of the Pd-catalyzed conjugate addition to 3-phenylcyclohexenone.

Initial results formed the desired product, (*R*)-3-phenyl-3-(*p*-tolyl)cyclohexan-1-one, **11** in low yields with a promising ee of 92% (entry 1). The primary byproduct of this reaction was from protodeboronation of the *p*-tolylboronic acid, forming toluene. It is hypothesized that coordination of the substrate and migratory insertion would be slower due to increased steric bulk at C3 of the substrate. With a slower reaction and a stoichiometric amount of water, hydrolysis to form boric acid and toluene is likely to occur. Therefore, subsequent conditions sought to minimize this unproductive pathway. Similar to previous observations in the analogous reaction of 3-methylcyclohexenone, omitting AgOTf stunted reactivity showing trace (<2% yield) product formation (entry 2). Changing the temperature to 80°C impeded the reaction, potentially by decomposing the active catalyst (entry 3). Increasing the amount of boronic acid to four equivalents to account for some decomposition slightly improved the yield (entry 4), while doubling the catalyst loading offered another marginal bump in yield (entry 5). Noting that this Pd-catalyzed conjugate addition reaction is homogenous,[10] 0.5 equivalents of tetrabutylammonium

bromide (TBABr) was added as a phase transfer catalyst to improve the solubility of the boronic acid in the organic phase.[17] This modification completely impeded reactivity (entry 6). However, using 18-crown-6 as a phase transfer catalyst maintained the same yield, but boosted the ee to 96% (entry 7). Adapting a protocol developed by Stanley and colleagues, a low concentration of boronic acid was maintained by portion wise addition of one equivalent every three hours.[14] This modification improved the yield to 26% and 92% ee (entry 8). Overall, a higher catalyst loading, 18-crown-6, and portion wise addition of boronic acid boosted the yield of the desired product to 48% with a 94% ee (entry 9). These conditions were used for subsequent optimization.

3.6 Conjugate addition to 3-phenylcyclohexenone reaction optimization, continued

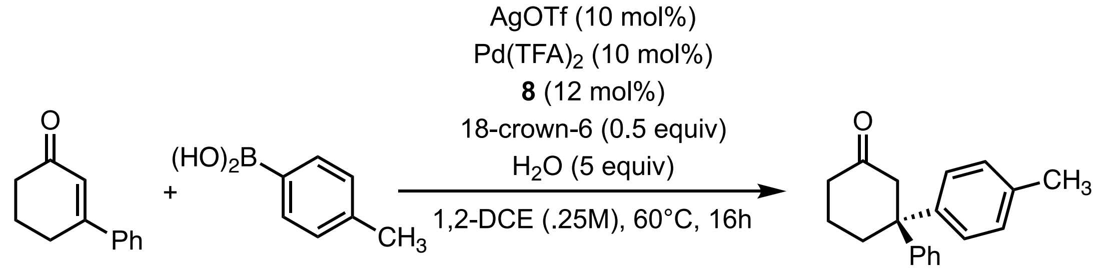

1 equiv
0.250 mmol

3 equiv, portionwise

12

Entry	Deviation from Standard Conditions	NMR Yield (Isolated Yield)	*ee*
1	1 equiv H_2O	42% (n.d.)	n.d.
2	50°C	36% (n.d.)	n.d.
3	10 mol% $Yb(OTf)_3$	57% (52%)	92%
4	10 mol% $AlCl_3$[a]	*trace*	n.d.
5	10 mol% $Sc(OTf)_3$	74% (59%)	92%
6	20 mol% $Sc(OTf)_3$	49% (n.d.)	n.d.
7	no 18-c-6, 10 mol% $Sc(OTf)_3$	30% (n.d.)	n.d.
8	18 mol% Ligand, 10 mol % $Sc(OTf)_3$	60% (54%)	92%
9	1 equiv H_2O, 10 mol% $Sc(OTf)_3$	44% (n.d.)	n.d.
10	65°C, 10 mol % $Sc(OTf)_3$	55% (47%)	90%
11	10 mol% AgOTf, 10 mol% $Sc(OTf)_3$	58% (n.d.)	n.d.
12	12 mol% AgOTf, 10 mol% $Sc(OTf)_3$	68% (n.d.)	n.d.
13	15 mol% AgOTf, 10 mol% $Sc(OTf)_3$	63% (n.d.)	n.d.
14	20 mol% AgOTf, 10 mol% $Sc(OTf)_3$	58% (n.d.)	n.d.

[a] Used **9** instead of **8**

Table S5. Second optimization iteration of the Pd-catalyzed conjugate addition to 3-phenylcyclohexenone.

The primary cause of protodeboronation was hypothesized to be the presence of superstoichiometric water. Decreasing the loading of water from five equivalents to one equivalent slightly depressed the yield (entry 1), suggesting that it is needed in excess to efficiently turn over the catalytic cycle. Lowering the temperature to 50°C significantly depressed product formation (entry 2). This is most likely due to migratory insertion (the rate-determining step)[10] reaction being

slower than the rate of protodeboronation. To increase the speed of the reaction, Lewis acid additives were screened. A catalytic amount of $Yb(OTf)_3$ did indeed improve the yield (entry 3), while $AlCl_3$ shut down reactivity (entry 5). $Sc(OTf)_3$ improved the reaction, delivering the product in 74% yield with 92% ee (entry 5). While the reason for this effect is unclear, it is hypothesized that $Sc(OTf)_3$ will have improved solubility in 1,2-DCE which is key for this transformation. Interestingly, doubling the catalytic loading of $Sc(OTf)_3$ decreased the yield (entry 6). With this result, we sought to retain the catalytic amount of $Sc(OTf)_3$ and remove additives to simplify the reaction conditions. Removing 18-crown-6 (entry 7), increasing the catalytic loading of ImOx (entry 8), reducing the amount of water (entry 9), and increasing the temperature to 65°C (entry 10) did not improve the product yield or enantioselectivity. These findings are consistent with previous trials. Finally, the catalytic loading of AgOTf was screened while maintaining a constant loading of $Sc(OTf)_3$. While there was not a significant impact on the yield, 12 mol% of AgOTf gave the best result, which was carried forward (entries 11-14).

3.6 Conjugate addition to 3-phenylcyclohexenone reaction optimization, continued

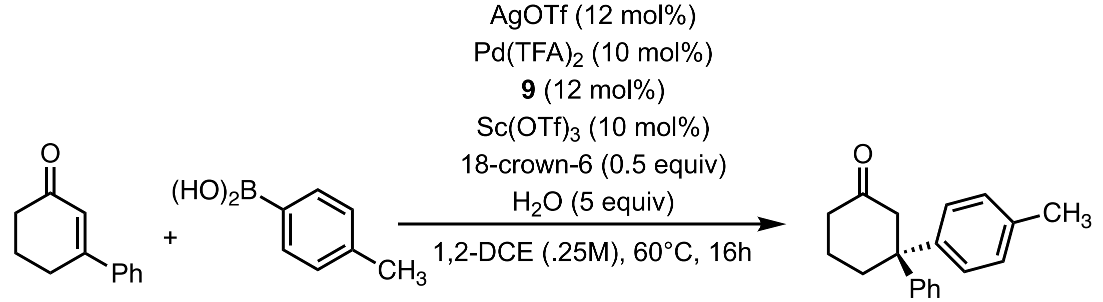

1 equiv
0.250 mmol

3 equiv, portionwise

12

Entry	Deviation from Standard Conditions	NMR Yield (Isolated Yield)	*ee*
1	6-indonlylboronic acid instead of *p*-tolylboronic acid	n.r.	n.d.
2	*p*-tolyl boronic ester	*trace*	n.d.
3	on a shaker	42%	n.d.
4	10 mol% $AgPF_6$ instead of AgOTf	35% (n.d.)	n.d.
5	No H_2O	26%	n.d.
6	2.0 molar	58%	n.d.
7	2.0 molar, no $Sc(OTf)_3$, no 18-crown-6	32%	n.d.
8	5 equiv MeOH instead of H_2O	79% (79%)	98%
9	5 equiv MeOH (repeat), no H_2O	(87%)	98%
10	5 equiv MeOH, no H2O, no 18-6-c	(61%)	94%
11	5 equiv MeOH, no H_2O, no $Sc(OTf)_3$	24% (n.d.)	n.d.
12	5 equiv MeOH, no H_2O, no $Sc(OTf)_3$, no 18-c-6	23% (n.d.)	n.d.

Table S6. Third optimization iteration of the Pd-catalyzed conjugate addition to 3-phenylcyclohexenone.

In the final iteration of reaction optimization, we initially sought to expand the substrate scope by using a challenging nucleophile. Using 6-indonylboronic acid did not form the desired product (entry 1). This indicated that the reaction conditions were not as robust as we had hoped;

therefore, further optimization was conducted. Other efforts to suppress protodeboronation included using a boronic ester as a coupling partner, which only showed trace product yield (entry 2).[18] This result suggests that an anionic boronic acid species is required for transmetalation.[15, 19] Previous studies report that this reaction proceeds in the presence of a mercury drop, supporting the hypothesis that it is homogeneous.[10] In efforts to improve the homogeneity a mechanical shaker was tested. This condition showed a depressed yield and increased amount of protodeboronation product (entry 3). Seminal work done by Stoltz suggests that the ability to solubilize the active cationic Pd species can be modulated by salt additivies.[7] Switching AgOTf with $AgPF_6$ diminished the yield (entry 4), suggesting that the triflate anion is better at solubilizing the active cationic Pd species than the hexafluorophosphate anion. Increasing the concentration by nearly ten-fold was thought to accelerate reactivity; however, running this reaction at 2.0 molar with or without additives did not improve the yield as hoped (entries 6 and 7).[14] When replacing the water additive with methanol, we observed a sharp increase in product yield and ee (entry 8). This result was replicated to deliver the product in 87% yield with an excellent 98% ee (entry 9). Previous reports with similar reaction conditions found success using methanol as a solvent rather than an additive.[20] We hypothesized that the increased solubility of methanol in 1,2-DCE would make for a readily accessible proton source to turn over the catalytic cycle. Control experiments were performed, suggesting that all previously screened additives were key for the success of this reaction (table 5, entries 10-12).

3.7 Control experiments with ^{tBu}PyOx

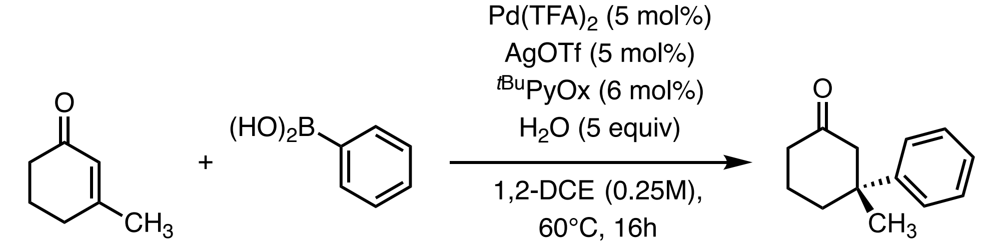

1 equiv, 0.250 mmol | 2 equiv | 88% yield, 90% ee

The reaction was conducted according to General Procedure D using ^{tBu}PyOx (3.14 mg, 0.015 mmol, 0.06 equiv) instead of ImOx. The desired product was isolated in a 88% yield with a 90% ee.

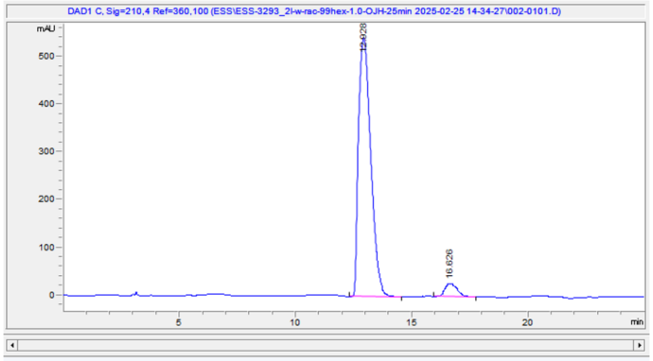

#	Time	Area	Height	Width	Area%	Symmetry
1	12.928	19944.2	544	0.5797	95.098	0.696
2	16.626	1028	27.3	0.4666	4.902	0.699

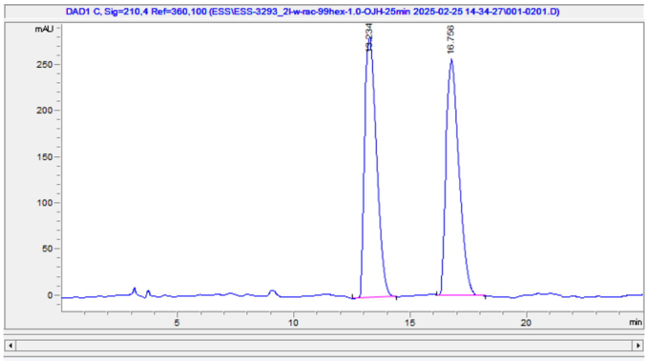

#	Time	Area	Height	Width	Area%	Symmetry
1	13.234	10233.5	282.6	0.5725	50.395	0.656
2	16.756	10073.2	256	0.615	49.605	0.668

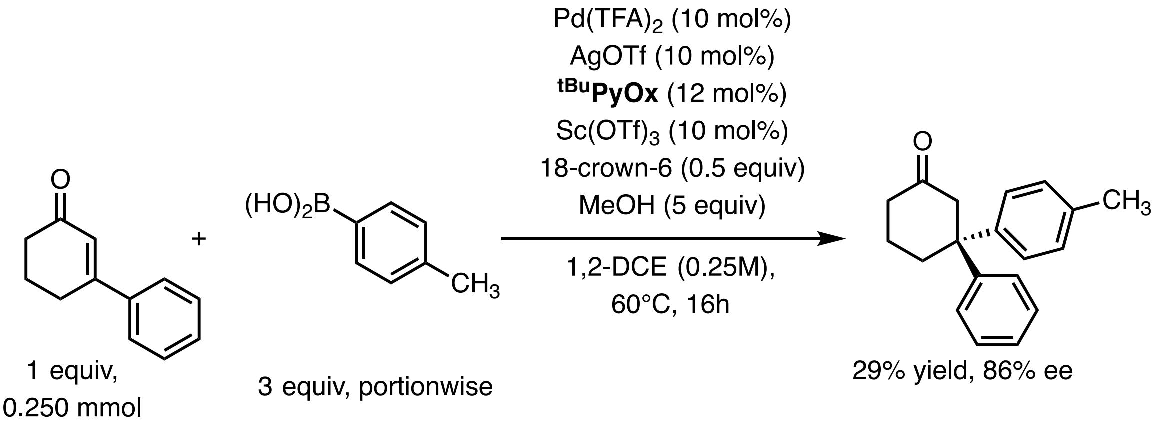

The reaction was conducted according to General Procedure E using ^{tBu}PyOx (6.13 mg, 0.030 mmol, 0.12 equiv) instead of ImOx. The desired product was isolated in a 29% yield with a 86% ee.

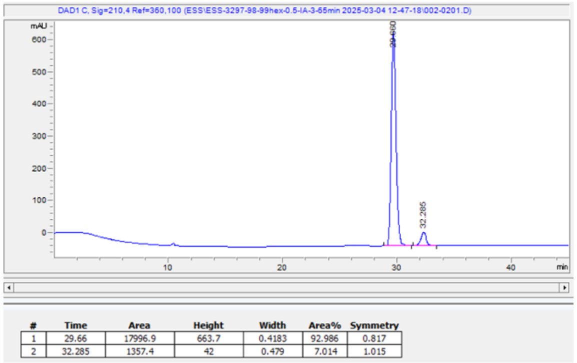

#	Time	Area	Height	Width	Area%	Symmetry
1	29.66	17996.9	663.7	0.4183	92.986	0.817
2	32.285	1357.4	42	0.479	7.014	1.015

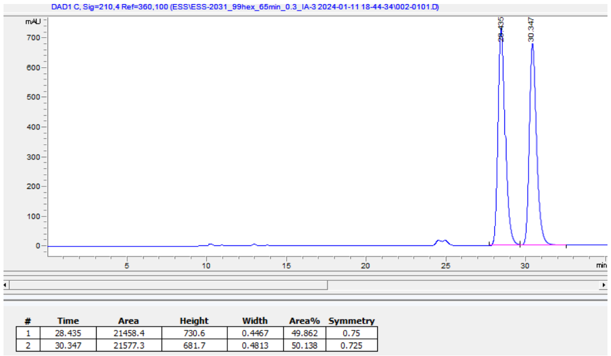

#	Time	Area	Height	Width	Area%	Symmetry
1	28.435	21458.4	730.6	0.4467	49.862	0.75
2	30.347	21577.3	681.7	0.4813	50.138	0.725

3.8 Procedure for the Reductive Conjugate Addition of Phenyliodide to 2-Cyclohexenone and Chiral Assays

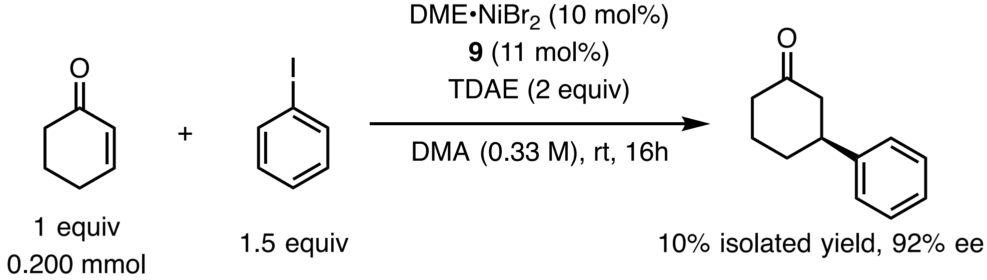

In an N_2-filled glovebox, a 4 mL vial with a stir bar was charged with DME•$NiBr_2$ (6.6 mg, 0.020 mmol, 0.10 equiv), ^{Dipp}Im^{tBu}Ox (7.2 mg, 0.022 mmol, 0.11 equiv), 2-cyclohexenone (19.4 μL, 0.200 mmol, 1 equiv), phenyliodide (33.6 μL, 0.300 mmol, 1.5 equiv), and TDAE (93.1 μL, 0.400 mmol, 2 equiv). 0.300 mL of DMA was added giving a turbid red solution and stirred at room temperature for 16 hours. The reaction was quenched by exposing to air. For GC yields, 19.5 μL of n-decane was added to the crude mixture and an aliquiot was filtered through celite into a GC vial. For isolated yields, the crude reaction was directly subjected to column chromatography (3:1 hexanes/Et_2O, R_F = 0.4) to afford the product as a clear oil (3.4 mg, 9.8% yield). The enantiomeric excess was determined by HPLC analysis (220 nm, 25°C), t_R 21.5 min (minor); t_R 23.6 min (major) [Chiralpak OD-H (4.6 mm I.D. x 250 mm L) hexane/*i*PrOH, 98:2, 0.5 mL/min]. All characterization data was consistent with previous reports.[21,22,23,24,25] Racemic products were synthesized by reducing 3-phenylcyclohexenone with H_2 gas and Pd/C.[26]

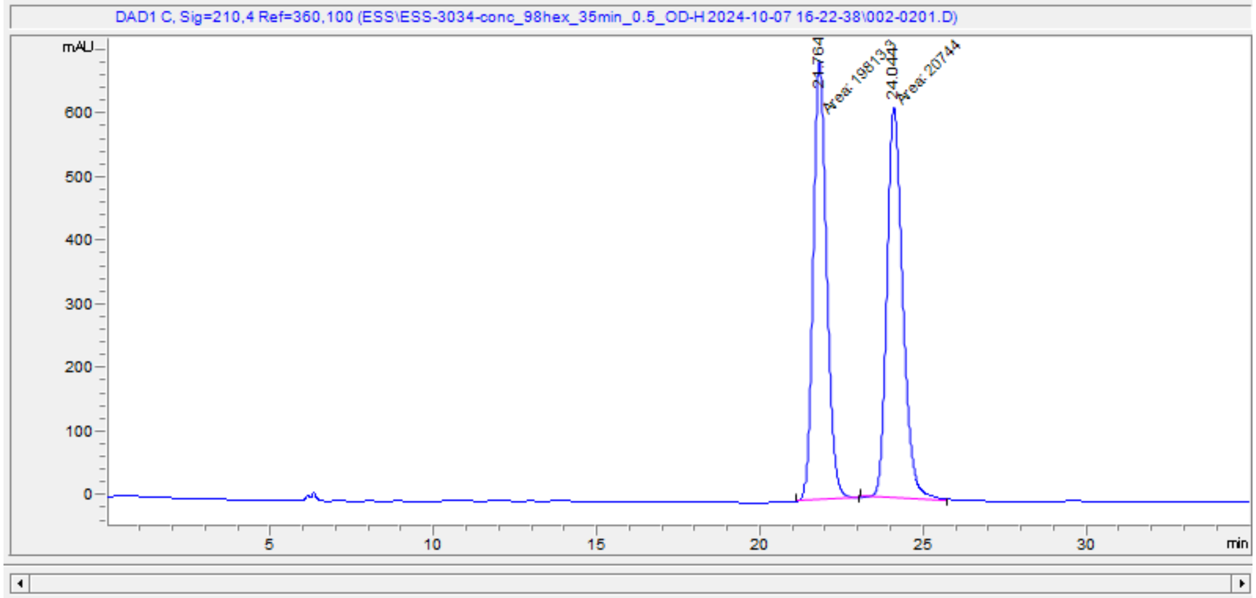

#	Time	Area	Height	Width	Area%	Symmetry
1	21.764	19813.3	691	0.4779	48.853	0.805
2	24.044	20744	614.9	0.5622	51.147	0.745

Figure S11. HPLC trace of racemic 3-phenylcyclohexenone.

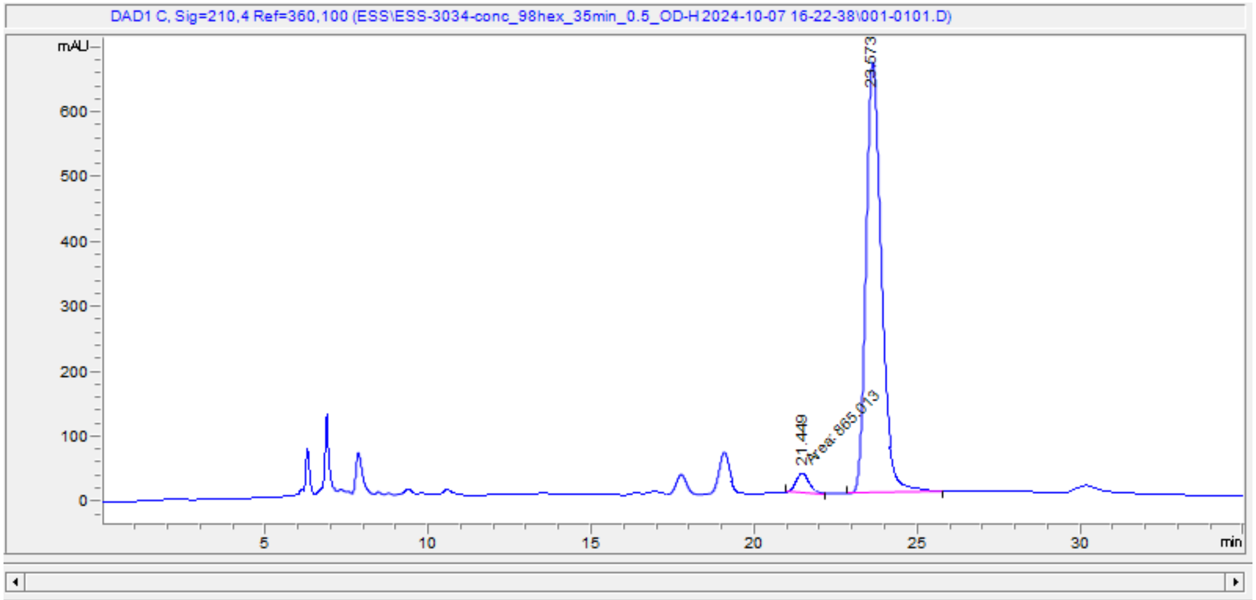

#	Time	Area	Height	Width	Area%	Symmetry
1	21.449	865	30.7	0.4693	3.758	0.873
2	23.573	22152.5	667.1	0.5034	96.242	0.699

Figure S12. HPLC trace of 3-phenylcyclohexenone using **9** as the chiral ligand (92% ee).

4. Synthesis of Palladium Complexes

$(^{Dipp}Im^{iPr}Ox)Pd^{II}(Ph)I$ **(15)**

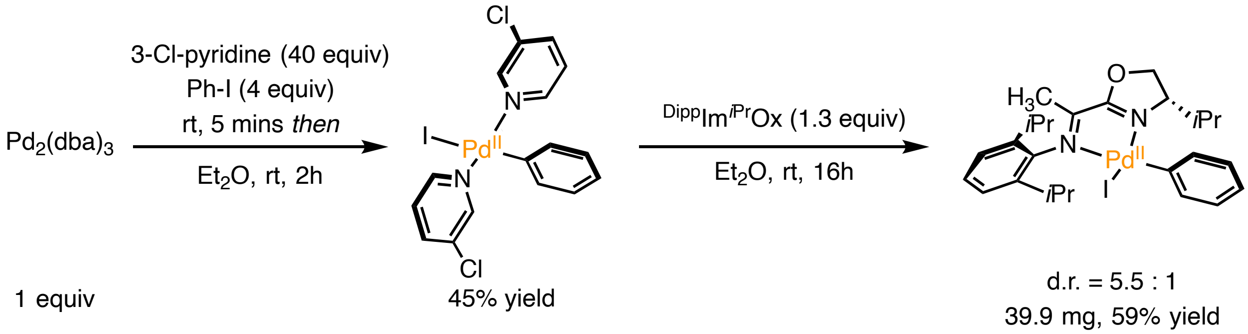

Performed in analogy to previous reported conditions.[27] In an N_2 filled glovebox, Pd_2(dba) (110 mg, 0.120 mmol, 1 equiv) was added to a vial containing a magnetic stir bar, 3-chloropyridine (546 µL, 4.80 mmol, 40 equiv), and phenyliodide (53.6 µL, 0.480 mmol, 4 equiv). The dark green suspension was stirred at ambient temperature for five minutes (became viscous) before adding 2 mL of Et_2O and stirring for an additional 2 hours. The heterogeneous mixture was filtered through a frit, washing with 3 x 5 mL of Et_2O and collecting into a vial. The compound was dried *in vacuo* to give $(3\text{-chloropyridine})_2Pd(Ph)I$ as a blue/grey solid (58 mg, 0.11 mmol, 45% yield). Due to the instability of the product, it was immediately used without further purification. A solution of $^{Dipp}Im^{iPr}Ox$ (44.1 mg, 0.140 mmol, 1.3 equiv) was suspended in Et_2O and added to a heterogeneous solution of $(3\text{-chloropyridine})_2Pd(Ph)I$ in Et_2O. The reaction mixture was stirred at ambient temperature for 16 hours. The crude reaction mixture was filtered through a plug of celite, washing with Et_2O and THF, and concentrated *in vacuo* to give a yellow/orange solid. The product was washed 3 x 15 mL pentane (until the washes were colorless) and recrystallized from Et_2O at -35°C, affording the title complex as a yellow/orange crystalline solid (39.9 mg, 59% yield).

^{1}H NMR (600 MHz, Benzene-*d$_6$*): δ 7.66 – 7.62 (m, 2H), 7.15 – 7.11 (m, 3H), 6.97 (m, 2H), 6.91 – 6.86 (m, 1H), 3.72 (dd, *J* = 9.1, 6.3 Hz, 1H), 3.66 (ddd, *J* = 9.8, 6.3, 3.4 Hz, 1H), 3.51 (dd, *J* = 10.1, 9.1 Hz, 1H), 3.22 (hept, *J* = 6.9 Hz, 1H), 2.96 (hept, *J* = 6.8 Hz, 1H), 1.65 (d, *J* = 6.8 Hz, 3H), 1.60 (d, *J* = 6.7 Hz, 3H), 1.43 (s, 3H), 1.27 – 1.20 (m, 1H), 1.01 (d, *J* = 6.9 Hz, 3H), 0.98 (d, *J* = 7.0 Hz, 3H), 0.49 (d, *J* = 6.9 Hz, 3H), 0.13 (d, *J* = 7.1 Hz, 3H). *Note: only peaks for major diastereomer is reported.
^{13}C{^{1}H} NMR (151 MHz, Benzene-*d$_6$*): δ 167.5, 162.5, 142.1, 138.1, 137.9, 137.9, 137.7, 127.7, 126.6, 123.9, 123.7, 123.4, 70.9, 67.7, 65.9, 29.7, 29.3, 28.2, 24.4, 24.0, 23.9, 23.6, 18.2, 18.1, 15.6, 13.4.
FT-IR: (cm^{-1}, neat, ATR) ṽ **=** 3358.82, 2959.61, 2359.76, 1561.79, 1467.15, 1211.95.
Analysis for $C_{26}H_{35}IN_2OPd$: Calculated C 49.97; H 5.65; N 4.48. Found C 50.37; N 5.12; H 4.48.

General Procedure F:

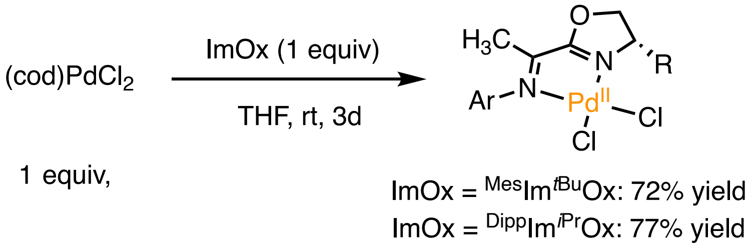

In an N_2 filled glovebox, yellow (cod)$PdCl_2$ (37.5 mg, 0.131 mmol, 1 equiv) was suspended in 2 mL THF. A solution of ImOx (0.131 mmol, 1 equiv) in 2 mL THF was added and the heterogeneous mixture was stirred at room temperature for three days. Solvent was removed *in vacuo*, washed 3 x 5 mL pentane, and further dried. Desired complex collected as an air stable orange powder. Crystals suitable for SC-XRD were grown by slow evaporation of DCM at room temperature.

($^{Dipp}Im^{iPr}Ox$)$Pd^{II}Cl_2$ **(19)**

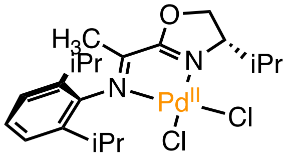

Compound prepared according to General Procedure F on a 0.17 mmol scale. Obtained as a fine orange powder (64.3mg, 77% yield).

^{1}H NMR (400 MHz, Acetonitrile-*d₃*): δ 7.38 – 7.30 (m, 1H), 7.27 – 7.20 (m, 2H), 4.82 (m, 1H)*, 4.77 (m, 1H)*, 4.40 (ddd, *J* = 9.8, 6.1, 3.5 Hz, 1H), 3.05 (dhept, *J* = 10.2, 6.8 Hz, 2H), 2.74 (dhept, *J* = 7.1, 3.5 Hz, 1H), 1.95 (s, 3H), 1.4 (d, *J* = 6.8 Hz, 3H), 1.4 (d, *J* = 6.8 Hz, 3H), 1.2 (d, *J* = 6.8 Hz, 3H), 1.1 (d, *J* = 6.9 Hz, 3H), 0.9 (d, *J* = 1.8 Hz, 3H), 0.9 (d, *J* = 2.0 Hz, 3H).
*Overlapping signal, so J-coupling could not be reported.

^{13}C{^{1}H} NMR (101 MHz, Acetonitrile-*d₃*): δ 170.54, 170.52, 140.78, 140.75, 140.36, 129.57, 124.68, 124.54, 73.29, 68.55, 30.03, 29.68, 29.38, 24.02, 23.97, 23.86, 23.55, 19.46, 18.51, 14.41.

FT-IR: (cm^{-1}, neat, ATR) ṽ **=** 2960.16, 2869.12, 1645.94, 1437.54, 1225.76.

($^{Mes}Im^{tBu}Ox$)$Pd^{II}Cl_2$ **(20)**

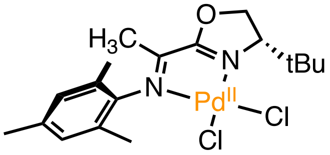

Compound prepared according to General Procedure F. Obtained as an orange powder (42 mg, 72% yield).

^{1}H NMR (400 MHz, Acetonitrile-*d₃*): δ 6.97 (s, 1H), 6.94 (s, 1H), 5.00 (dd, *J* = 9.8, 3.0 Hz, 1H), 4.73 (t, *J* = 9.8, 9.2 Hz, 1H), 4.15 (dd, *J* = 9.2, 3.0 Hz, 1H), 2.27 (s, 3H), 2.20 (s, 3H), 2.18 (s, 3H), 2.12 – 2.08 (m, 2H), 1.06 (s, 9H).

^{13}C{^{1}H} NMR (101 MHz, Acetonitrile-*d₃*): δ 171.03, 170.36, 140.79, 138.64, 130.27, 130.24, 129.66, 129.36, 75.59, 70.91, 55.28, 35.87, 31.61, 26.40, 20.92, 18.48, 18.40, 18.27.

FT-IR: (cm^{-1}, neat, ATR) ṽ **=** 2943.38, 1480.25, 1436.31, 1237.80.

(^{Mes}Im^{tBu}Ox)PdI$_2$ **(S1)**

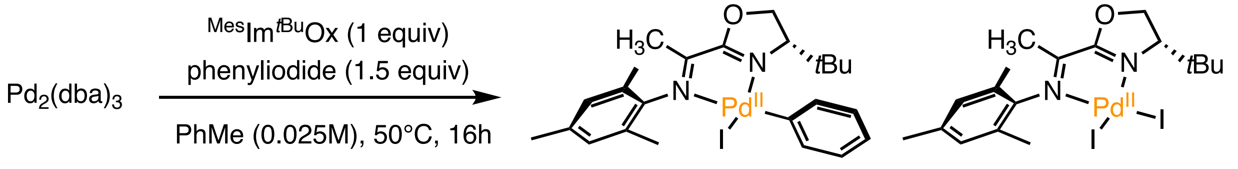

1 equiv | *desired, not observed* | **S1** *isolated*

Desired complex was synthesized in analogy to previous reported conditions.[10] In a 20 mL scintillation vial containing a stir bar, $Pd_2(dba)_3$ (31.0 mg, 0.034 mmol, 0.5 equiv) and ^{Mes}Im^{tBu}Ox (20.0 mg, 0.071 mmol, 1.05 equiv) were suspended in 1.4 mL toluene. Phenyliodide (11.0 µL, 0.10 mmol, 1.5 equiv) was added and the vial was heated to 50°C for 16 hours. Upon completion, the crude reaction mixture was filtered through celite and concentrated *in vacuo*. The orange residue was washed with 3 x 5 mL hexanes, extracted with 3 x 5 mL Et_2O, filtering through celite, and dried, affording the title complex as an orange powder (8.0 mg, 20% yield). Crystals suitable for XRD analysis were grown from a concentrated solution of Et_2O at -7°C.

^{1}H NMR (400 MHz, $CDCl_3$): δ 6.95 (s, 1H), 6.93 (s, 1H), 4.90 (dd, *J* = 9.4, 2.8 Hz, 1H), 4.74 (dd, *J* = 8.9, 2.7 Hz, 1H), 4.64 (t, *J* = 9.1 Hz, 1H), 2.31 (s, 3H), 2.19 (s, 3H), 2.16 (s, 3H), 1.90 (s, 3H), 1.11 (s, 9H).

5. Synthesis of Nickel Complexes

(^{Mes}Im^{tBu}Ox)Ni(cod), **21**

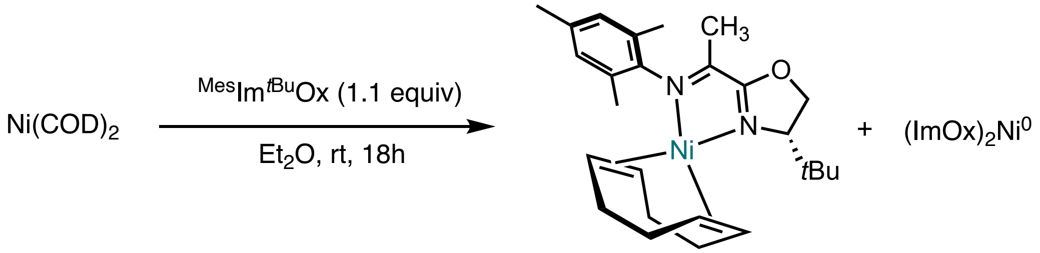

1 equiv *yellow* | **21** *deep violet*

In an N_2 filled glovebox, Ni(cod)$_2$ (10.0 mg, 0.036 mmol, 1 equiv) was suspended in ~2 mL of Et_2O. In a separate vial, ^{Mes}Im^{tBu}Ox (11.5 mg, 0.040 mmol, 1.1 equiv) was suspened ~2 mL of Et_2O and added dropwise to the vial containing Ni(cod)$_2$. The color of the solution slowly changed from yellow to deep violet over 10 minutes and was stirred at room temperature for 18 hours. The solution was filtered through a plug of celite, eluted with Et_2O, and dried *in vacuo*. The crude mixture was resuspended in pentane and recrystallized at -35°C overnight. The dark purple solution was decanted away from residual yellow Ni(cod)$_2$ and allowed to recrytallize in pentane at -35°C. Due to the instability of the compound from facile cod dissociation and competing bis-ligand formation in solution, the resulting characterization from recrystallization is a complex mixture.

^{1}H NMR (400 MHz, C_6D_6): δ 7.06 (s, 1H), 6.75 (s, 1H), 4.18 – 4.01 (m, 4H), 3.77 – 3.69 (m, 1H), 3.68 – 3.54 (m, 2H), 2.89 – 2.72 (m, 2H), 2.65 (s, 3H), 2.64 – 2.52 (m, 1H), 2.28 (s, 3H), 1.89 – 1.76 (m, 2H), 1.49 (s, 3H), 1.45 – 1.33 (m, 2H), 1.33 (s, 9H), 1.29 – 1.16 (m, 1H), 0.07 (s, 3H).

HRMS (ESI) m/z: [M - cod + ^{Mes}Im^{tBu}Ox + Na]$^+$ Calculated for $C_{36}H_{52}N_4NiO_2Na^+$ 653.3341; Found 653.3261.

($^{Mes}Im^{iPr}Ox$)Ni(cod) **(S2)**

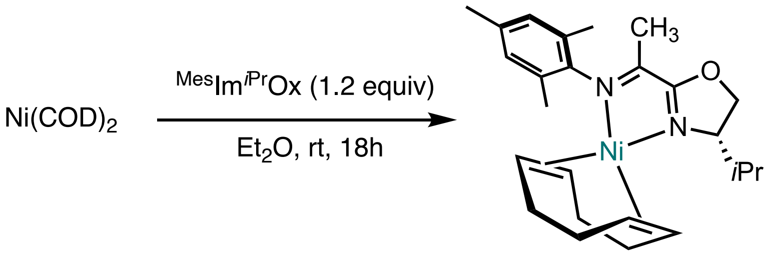

1 equiv
yellow

S2
deep violet

In an N_2 filled glovebox, Ni(cod)$_2$ (24.0 mg, 0.087 mmol, 1 equiv) was suspended in ~2 mL of Et_2O. In a separate vial, $^{Mes}Im^{iPr}Ox$ (27.3 mg, 0.100 mmol, 1.2 equiv) was suspened ~2 mL of Et_2O and added dropwise to the vial containing Ni(cod)$_2$. The color of the solution slowly changed from yellow to deep violet over 10 minutes and was stirred at room tempreature for 18 hours. The solution was filtered through a plug of celite, eluted with Et_2O, and dried *in vacuo*. The crude mixture was then extracted with cold pentane (chilled in a -35°C freezer for 30 minutes) and dried *in vacuo.* The crude mixture was resuspended in pentane and recrystallized at -35°C overnight. The dark purple solution was decanted away from residual yellow Ni(cod)$_2$ and allowed to recrytallize in pentane at -35°C. Due to the instability of the compound from facile cod dissociation and competing bis-ligand formation in solution, the resulting characterization from recrystallization is a complex mixture.

^{1}H NMR (400 MHz, C_6D_6): δ 7.05 (s, 1H), 6.80 (s, 1H), 4.18 – 4.12 (m, 1H), 4.11 – 4.02 (m, 1H), 4.02 – 3.91 (m, 3H), 3.89 – 3.83 (m, 1H), 3.80 – 3.71 (m, 1H), 3.44 – 3.31 (m, 1H), 2.80 – 2.67 (m, 1H), 2.53 (s, 3H), 2.51 – 2.38 (m, 1H), 2.28 (s, 3H), 2.02 – 1.93 (m, 2H), 1.58 (s, 3H), 1.50 – 1.39 (m, 1H), 1.28 – 1.12 (m, 3H), 1.03 (d, *J* = 6.8 Hz, 3H), 0.82 (d, *J* = 6.8 Hz 3H), 0.18 (s, 3H).

($^{Dipp}Im^{tBu}Ox$)Ni^{II}(Mes)Br **(22)**

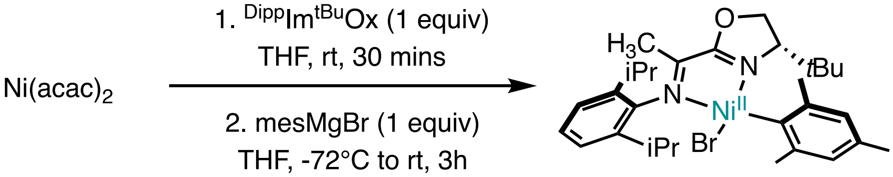

1 equiv

22
red/purple
97% yield (63.0 mg)

Performed in analogy to previous reported conditions.[28] In an N_2 filled glovebox, Ni(acac)$_2$ (28.5 mg, 0.111 mmol, 1 equiv) and $^{Dipp}Im^{tBu}Ox$ (36.7 mg, 0.111 mmol, 1 equiv) were added to a vial containing a magnetic stir bar and suspended in ~2 mL of THF. The contents were stirred at room temperature for 30 minutes, where color gradually changed from a blue/green heterogeneous mixture to a teal homogenous solution. The vial was then cooled to -72°C in a cold well (dry ice/ethanol) and a 1.0 M solution of mesitylmagnesium bromide in THF (111 µL, 0.111 mmol, 1 equiv) was added dropwise. The color immediately changed to deep purple/red. The vial removed from the cold well and stirred at room temperature for 3 hours, after which was filtered through a plug of celite, eluted with THF, and dried *in vacuo.* The dark purple/red oil was resuspended in ~5 mL of Et_2O and stirred at room temperature for 1 hour before filtering through a plug of celite, eluting with Et_2O, and dried in vacuo. The crude product was washed 3 x 5 mL with pentane, affording the title complex as a deep purple/red powder (63.0 mg, 97% yield). Crystals suitable for SC-XRD analysis were grown from a concentrated solution of Et_2O at -35°C.

^{1}H NMR (600 MHz, Benzene-d_6): 7.15 – 7.11 (m, 2H), 7.11 – 7.05 (m, 1H), 6.69 (s, 1H), 6.62 (s, 1H), 4.31 (hept, J = 6.8 Hz, 1H), 3.85 (dd, J = 9.6, 2.6 Hz, 1H), 3.63 (dd, J = 9.6, 8.6 Hz, 1H), 3.37 (s, 6H), 3.00 (dd, J = 8.5, 2.4 Hz, 1H), 2.84 (hept, J = 6.8 Hz, 1H), 2.31 (s, 3H), 1.77 (d, J = 6.8 Hz, 3H), 1.65 (d, J = 6.7 Hz, 3H), 1.25 (s, 3H), 1.05 (d, J = 6.9 Hz, 3H), 1.00 (d, J = 7.0 Hz, 3H), 0.45 (s, 9H).
^{13}C{^{1}H} NMR (151 MHz, Benzene-d_6): δ 196.7, 185.7, 167.7, 158.4, 145.1, 141.5, 140.2, 140.1, 138.7, 134.8, 132.2, 126.3, 125.7, 123.8, 123.4, 102.2, 74.6, 69.8, 34.7, 30.2, 30.1, 29.1, 27.8, 26.2, 25.4 (d, J = 5.0 Hz), 24.3, 23.6, 23.0, 20.7, 17.6.
FT-IR: (cm^{-1}, neat, ATR) ṽ **=** 2959.27, 2867.84, 1593.99, 1517.31, 1394.77

[^{Dipp}Im^{tBu}Ox)Ni(mes)Br]$^{\bullet-}$ **(23)**

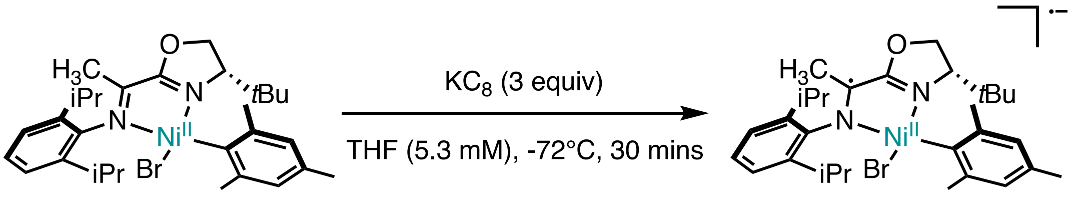

22
deep red/purple
1 equiv

23
golden brown
EPR Active

In an N_2-filled glovebox, **22** (4.7 mg, 8.0 µmol, 1 equiv) and KC_8 (3.3 mg, 24.0 µmol, 3 equiv) were weighed in separate vials, each suspended in 750 µL of THF (1.5 mL total), and cooled to -72°C in a cold well (dry ice/ethanol). The heterogeneous mixture of KC_8 was added to the vial containing **22** and vigorously shaken for 1 minute. The color immediately changed from deep red/purple to golden brown. After 30 minutes at -72°C, the solution was filtered directly into an EPR tube.

[^{Dipp}Im^{tBu}Ox]$^-$ **(S3)**

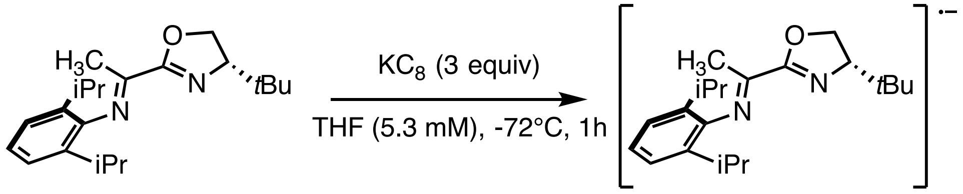

9
pale yellow
2.8 mg (8.5 umol)
1 equiv

S3
light blue
EPR Active

The title compound was prepared in analogy to **23.**

($^{Dipp}Im^{tBu}Ox$)$NiBr_2$ **(S4)**

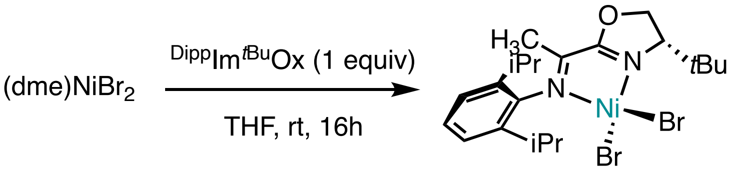

1 equiv

S4

44.1 mg, 92% yield

In an N_2 filled glovebox, orage (dme)$NiBr_2$ (28.0 mg, 0.087 mmol, 1 equiv) was suspended in 2 mL THF. A solution of $^{Dipp}Im^{tBu}Ox$ (0.087 mmol, 1 equiv) in 2 mL THF was added and the heterogeneous mixture became homogenous red. The reaction was stirred at room temperature for 16 hours. Solvent was removed *in vacuo*, washed 3 x 5 mL pentane, triturated 2 x 3 mL Et_2O and further dried. Desired complex collected as an orange powder (44.1 mg, 92% yield). Crystals suitable for SC-XRD were grown from a concentrated solution of THF at -35°C.

^{1}H NMR (400 MHz, $CDCl_3$): δ 21.37, 20.10, 9.82, 3.99, 2.03, 1.36, 1.16, 1.01, -0.05, -0.35, -12.34, -26.89.
FT-IR: (cm^{-1}, neat, ATR) ṽ **=** 3362.16, 2959.24, 2868.45, 1630.50, 1361.42.

6. Decomposition of 3 to form 5

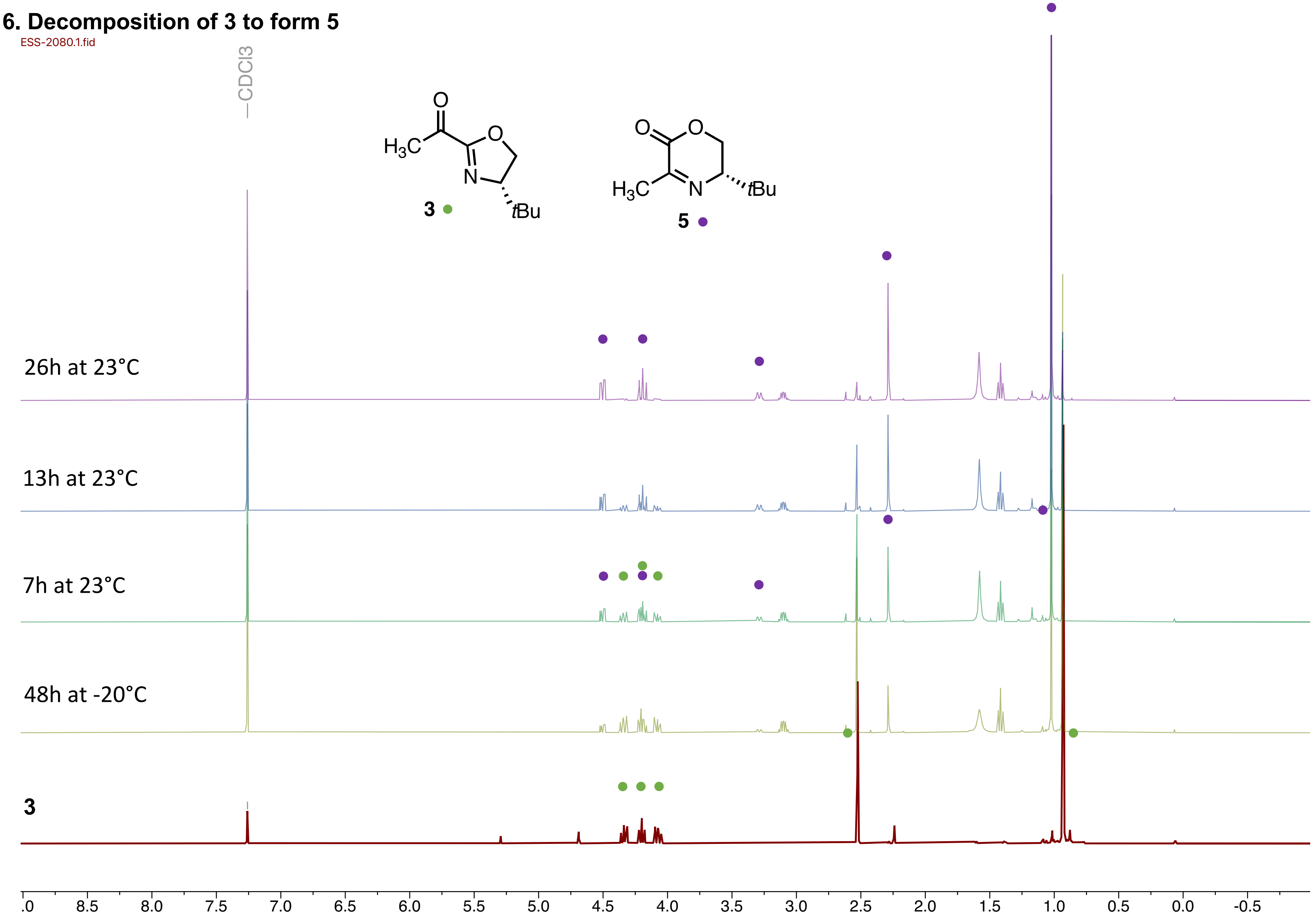

Figure S13. Monitoring decomposition of **3** to **5** by ^{1}H NMR ($CDCl_3$, 400 MHz, 23°C).

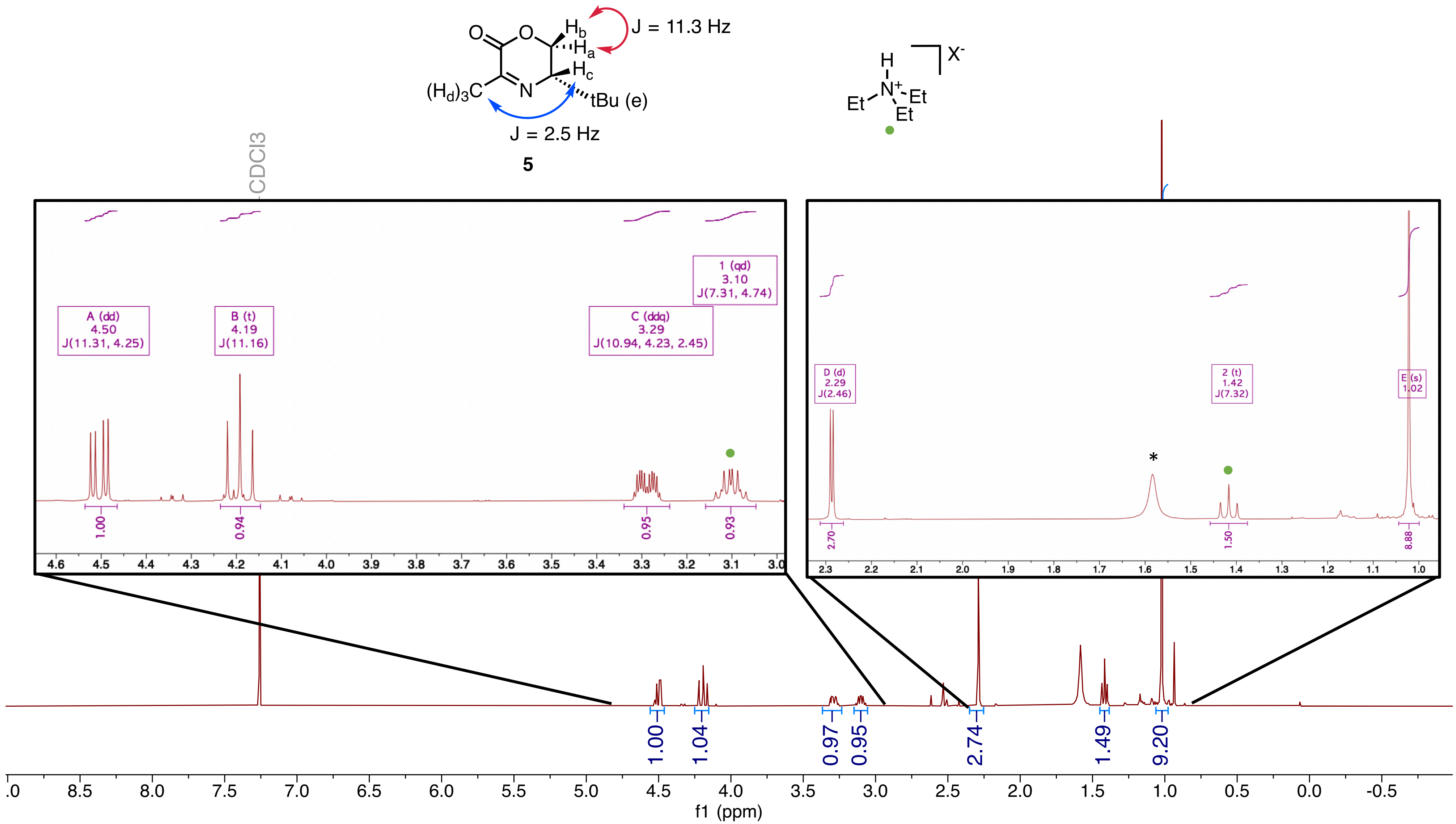

Figure S14. Decomposition of **3** to Form **5**, continued. * = water from NMR solvent.

7. Stoichiometric Experiments

7.1 Reductive Elimination of (^{Dipp}Im^{iPr}Ox)Pd(Ph)I to Form PhI

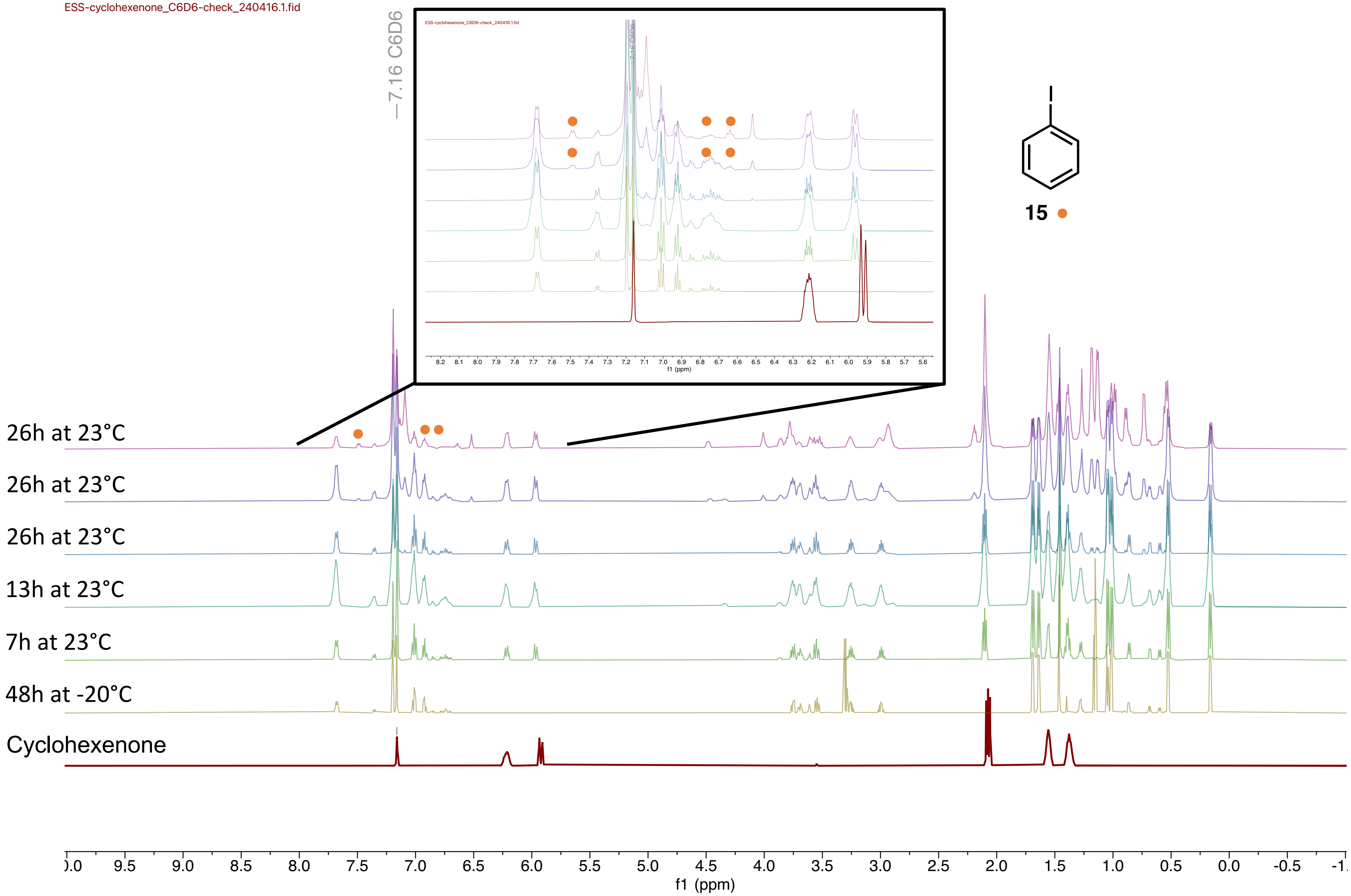

Figure S15. ^{1}H NMR (C_6D_6, 400 MHz, 23°C) monitoring of reaction progress.

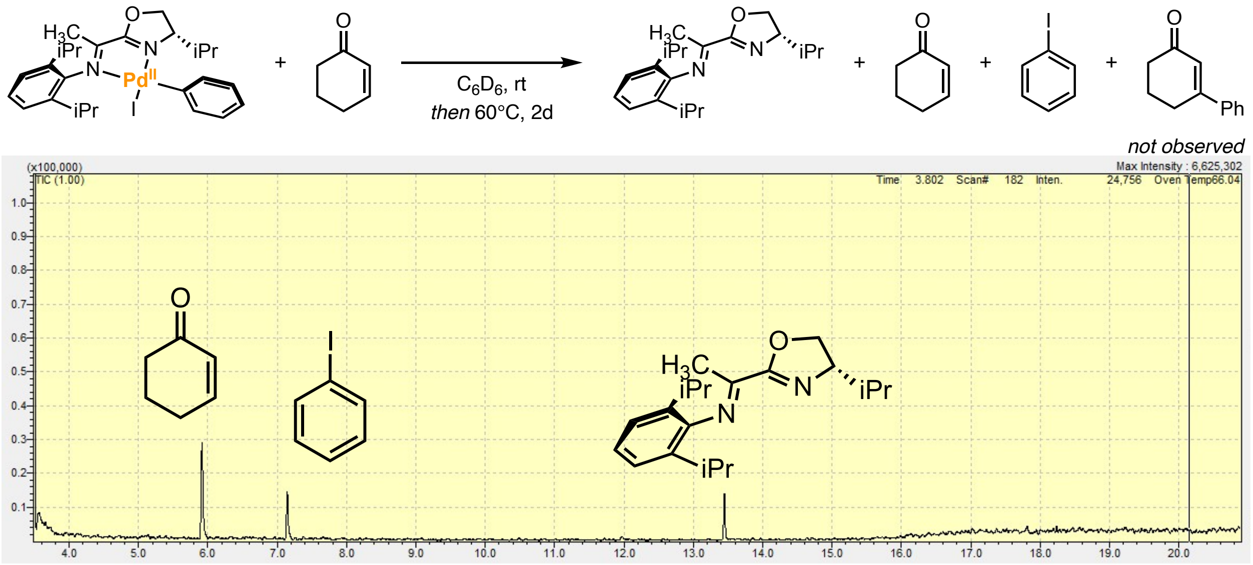

Figure S16. GCMS trace of stoichiometric experiment with **15** and **16**.

7.2 Insertion of (^{dipp}Im^{iPr}Ox)Pd(Ph)I to Cyclohexenone

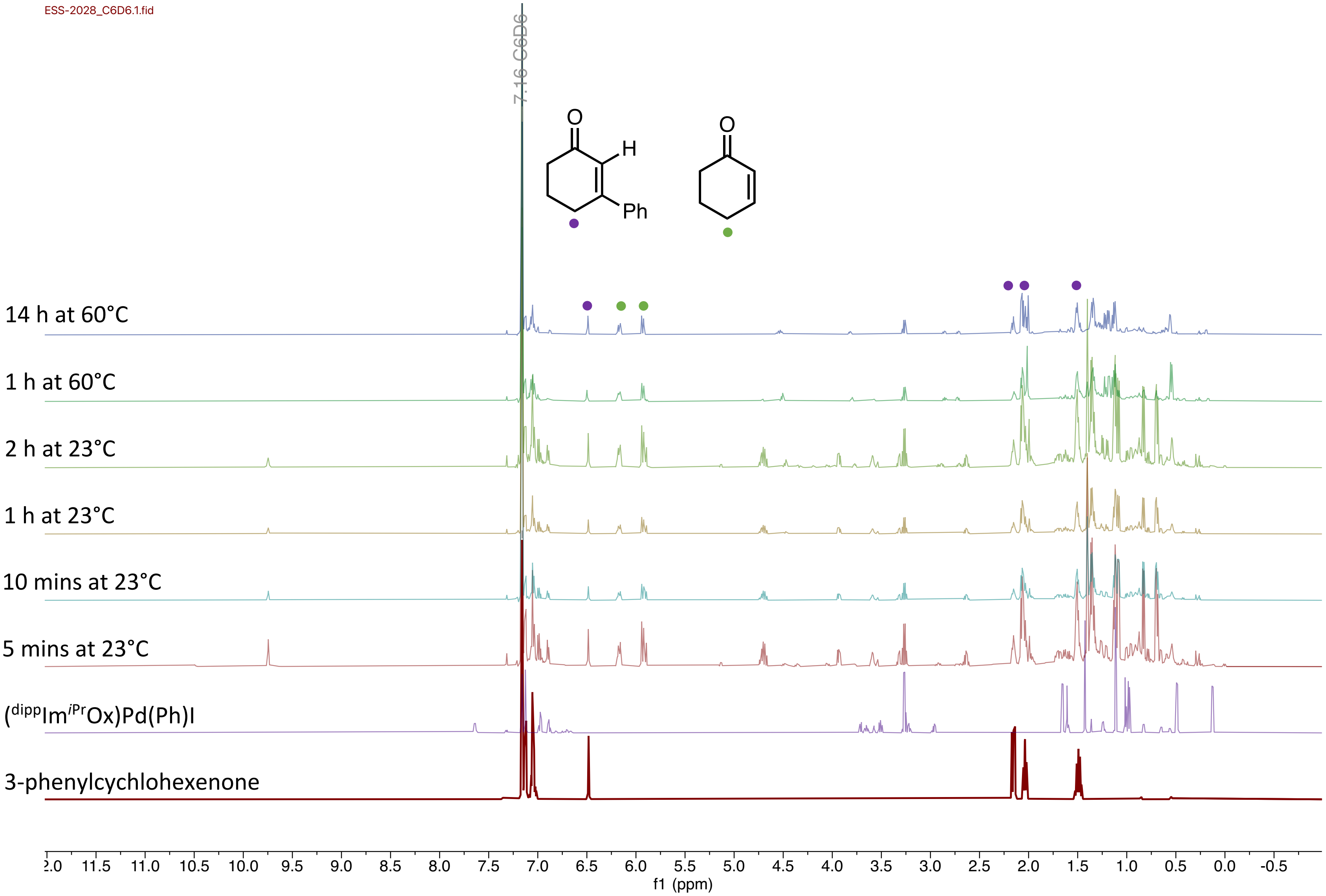

Figure S17. ^{1}H NMR (C_6D_6, 400 MHz, 23°C) monitoring of reaction progress.

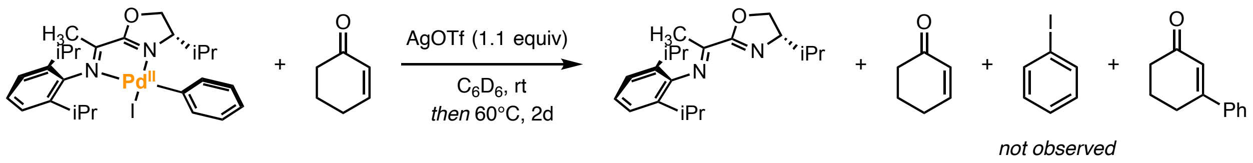

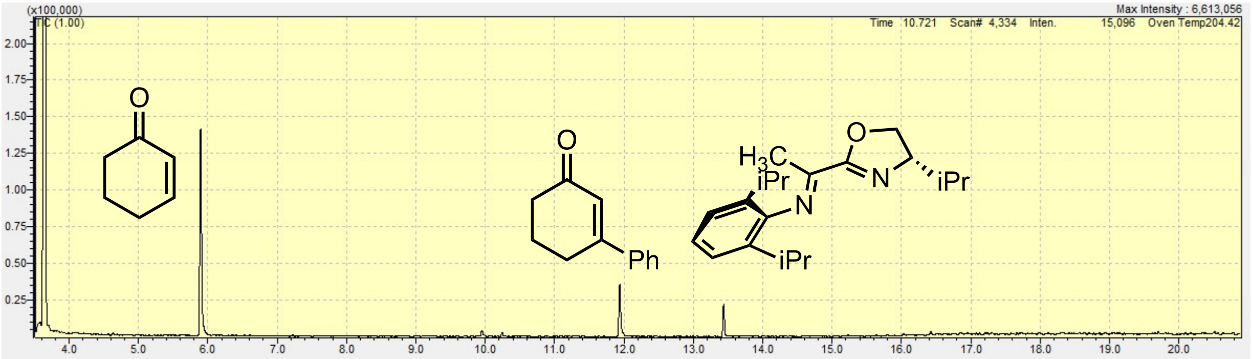

Figure S18. GCMS trace of stoichiometric experiment with **15**, **16**, and AgOTf.

7.3 Stoichiometric Reaction of ($^{dipp}Im^{iPr}Ox$)Pd(Ph)I with 3-methylcyclohexenone

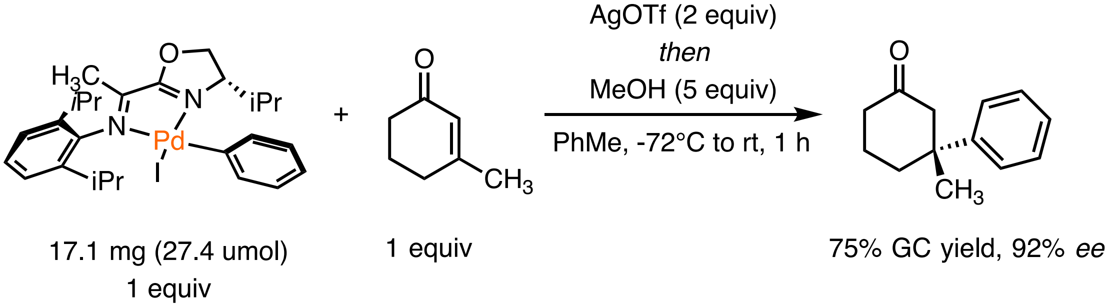

In an N_2 filled glovebox, ($^{dipp}Im^{iPr}Ox$)Pd(Ph)I (17.1 mg, 27.4 umol, 1 equiv) and AgOTf (14.1 mg, 54.8 umol, 2 equiv) were suspended in 0.70 mL PhMe (39 mM) and cooled to -72°C in a cold well for 30 minutes. 3-methylcyclohexenone (3.30 uL, 27.4 umol, 1 equiv) was added and the vial was removed from the cold well. Immediately, methanol (5.54 uL, 137 umol, 5 equiv) was added. After stirring for 1 hour at room temperature, the vial was removed from the glovebox, exposed to air, and analyzed by GC/GCMS. The product was isolated by column chromatography (100% DCM) and the ee was measured in accordance with the procedure detailed in section **3.1**.

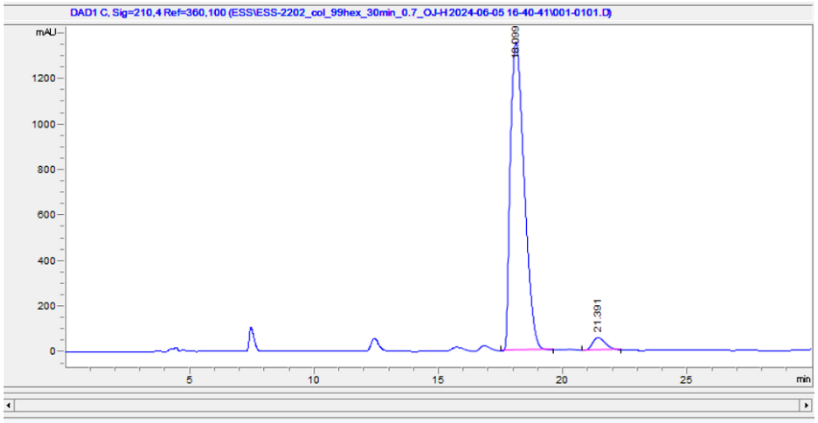

#	Time	Area	Height	Width	Area%	Symmetry
1	18.099	52444.1	1354.3	0.6198	96.431	0.641
2	21.391	1941.1	54.3	0.5567	3.569	0.686

Figure S19. HPLC trace of **10** using **7** as the chiral ligand. (0.7 mL/min flow rate instead of 1.0 mL/min; 92% ee).

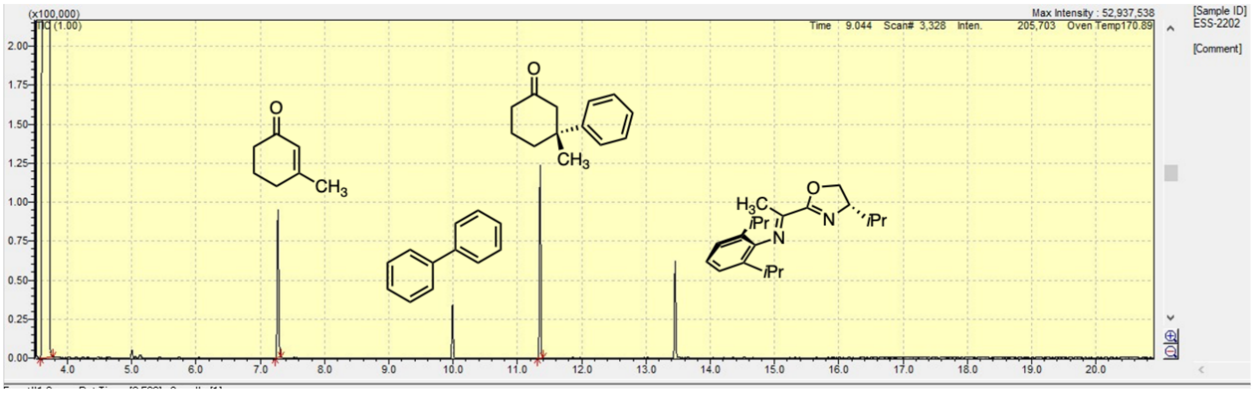

Figure S20. GCMS trace of reaction.

8. Conjugate addition to 3-methylcyclohexenone with NH_4PF_6

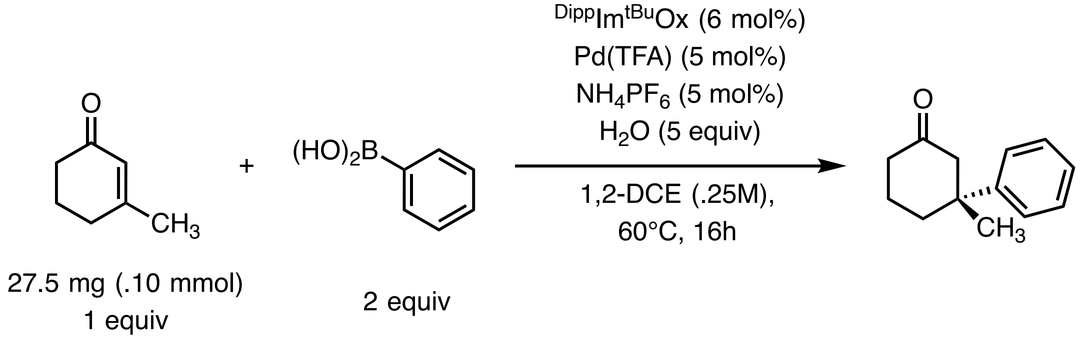

Experiment conducted following the procedure detailed in section 3.1.

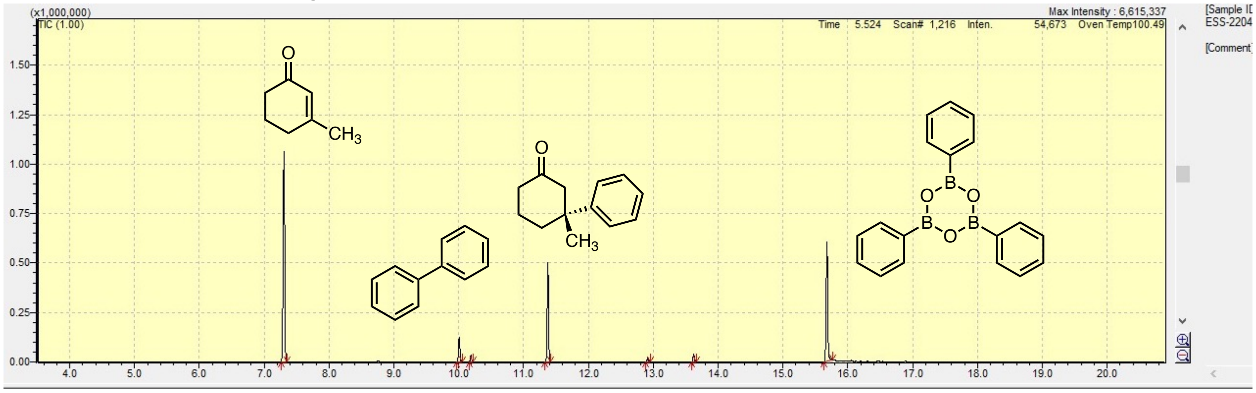

Figure S21. GCMS trace of crude reaction.

9. Conjugate addition to 3-methylcyclohexenone with Tl(OTf)

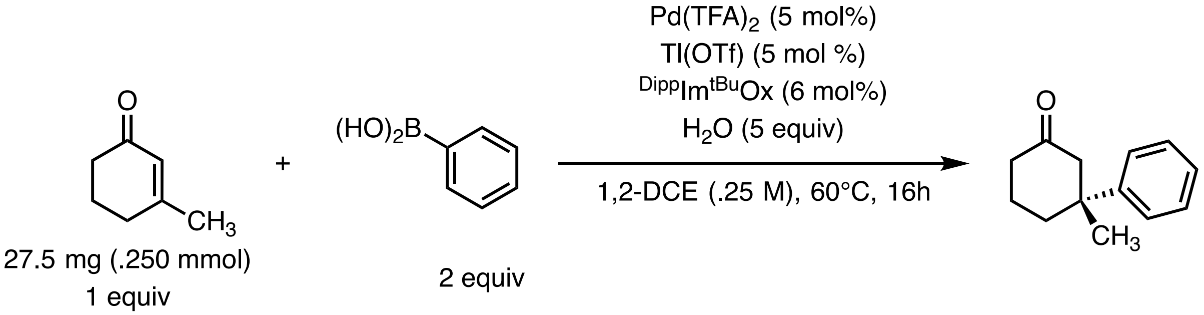

Experiment conducted following the procedure detailed in section 3.1.

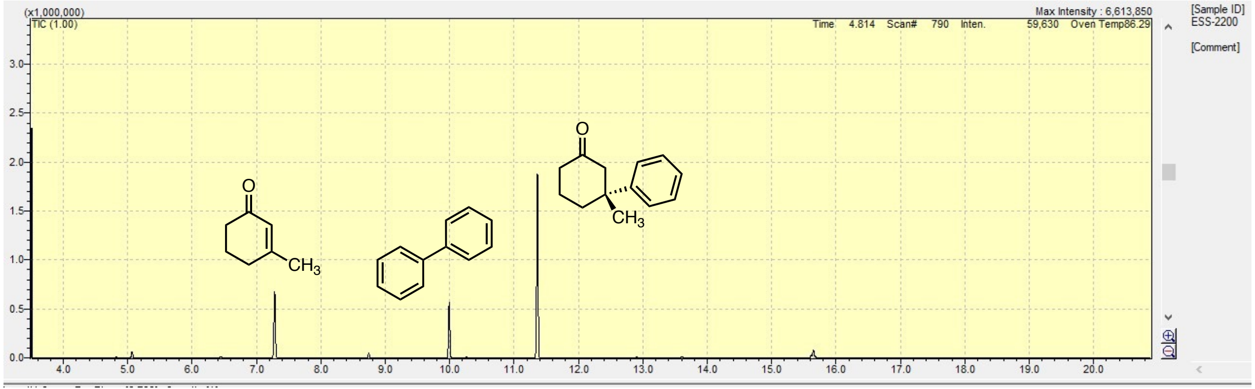

Figure S22. GCMS trace of crude reaction.

10. NMR Spectra

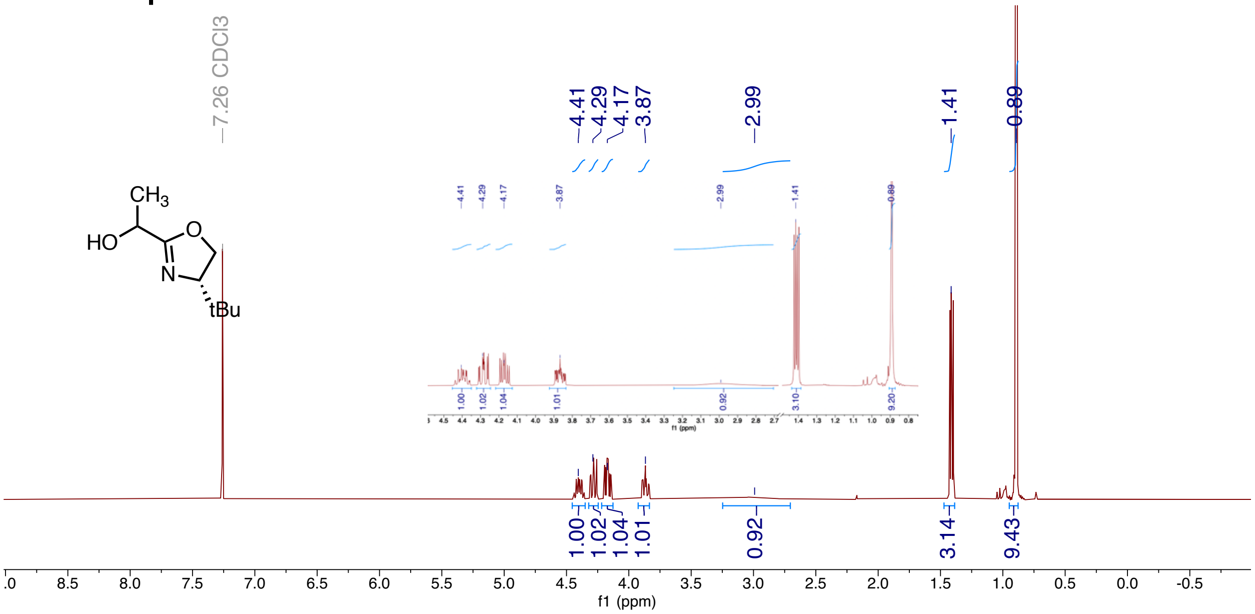

Figure S23. ^{1}H NMR ($CDCl_3$, 400 MHz, 23°C) spectrum of **1**. *Note: two diastereomers are observed.

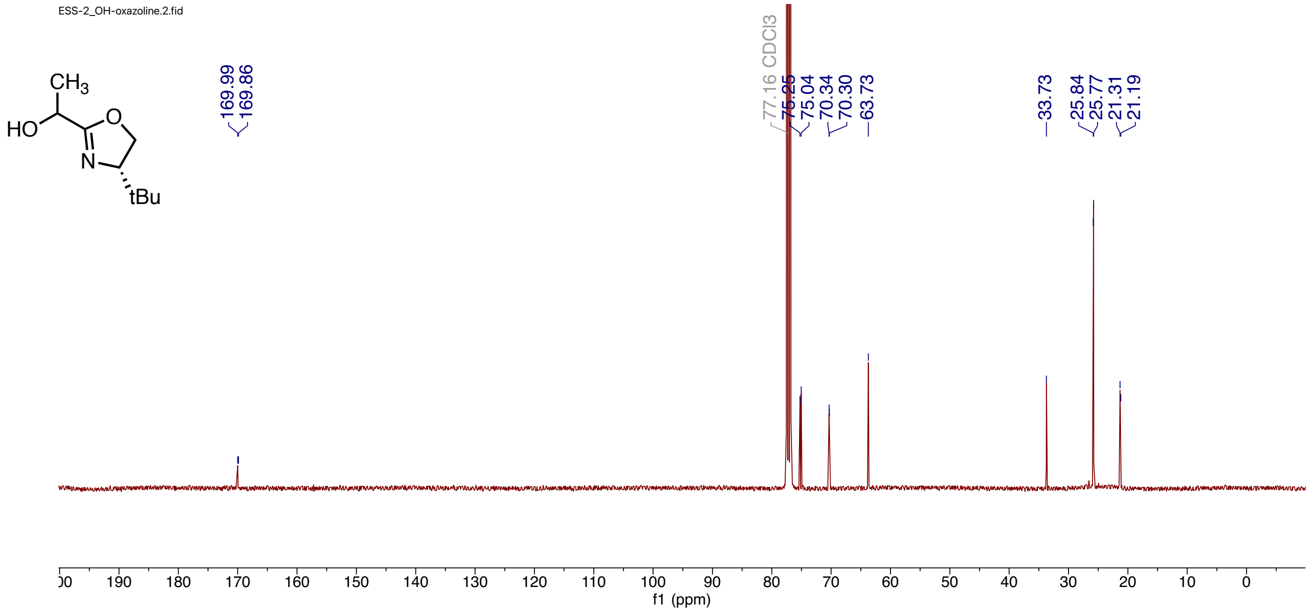

Figure S24. ^{13}C NMR ($CDCl_3$, 400 MHz, 23°C) spectrum of **1** in chloroform-*d.*

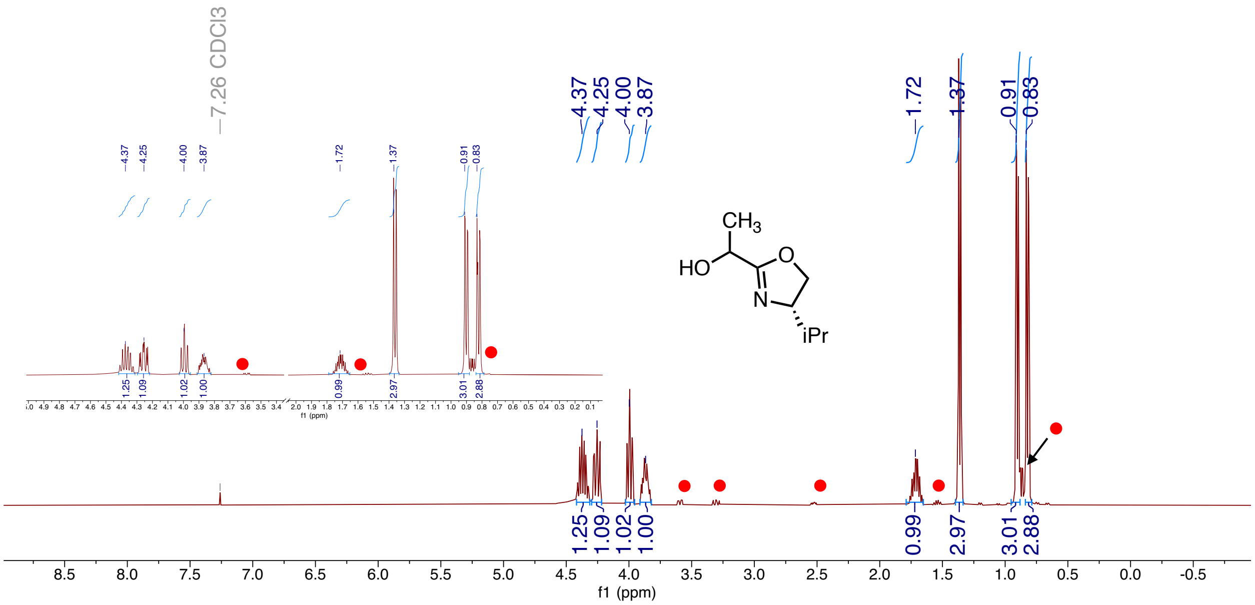

Figure S25. ^{1}H NMR spectrum of **2** in chloroform-*d*. Note: red circles indicate (*S*)-valinol.

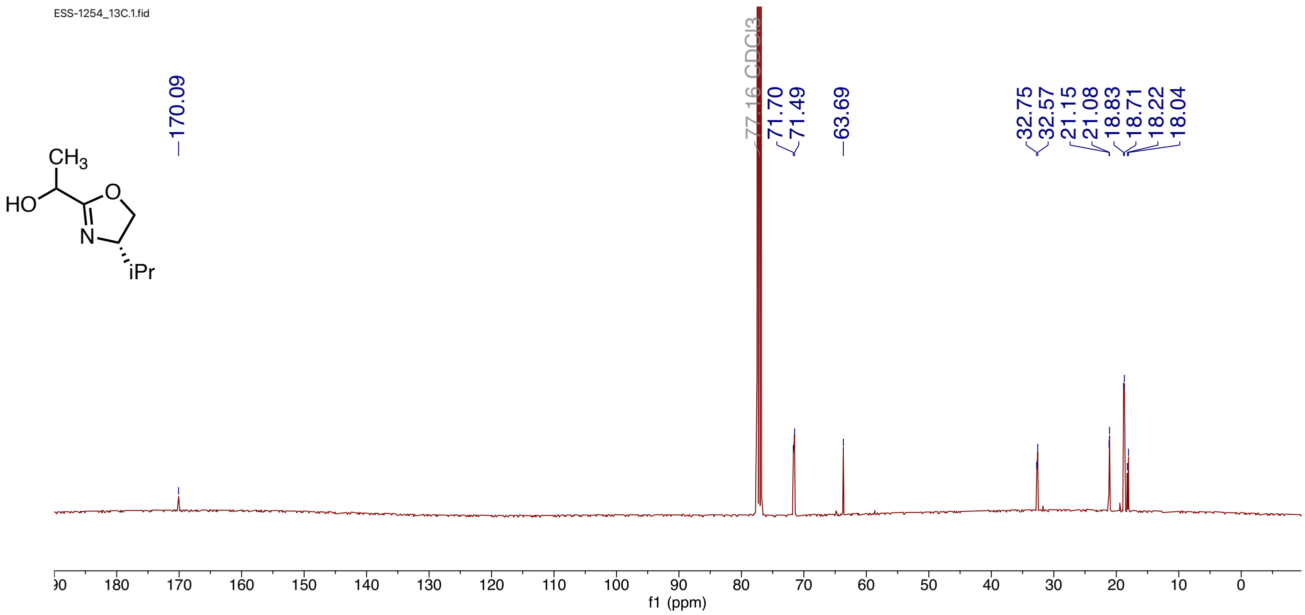

Figure S26. ^{13}C NMR spectrum of **2** in chloroform-*d*.

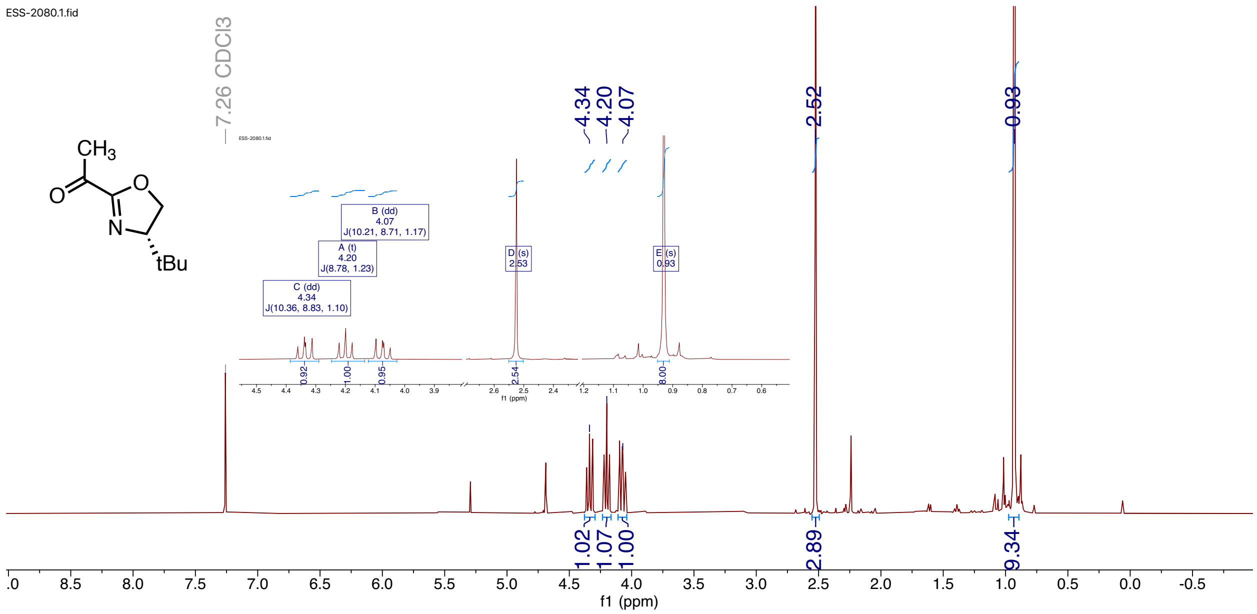

Figure S27. ^{1}H NMR spectrum of crude **3** in chloroform-*d*.

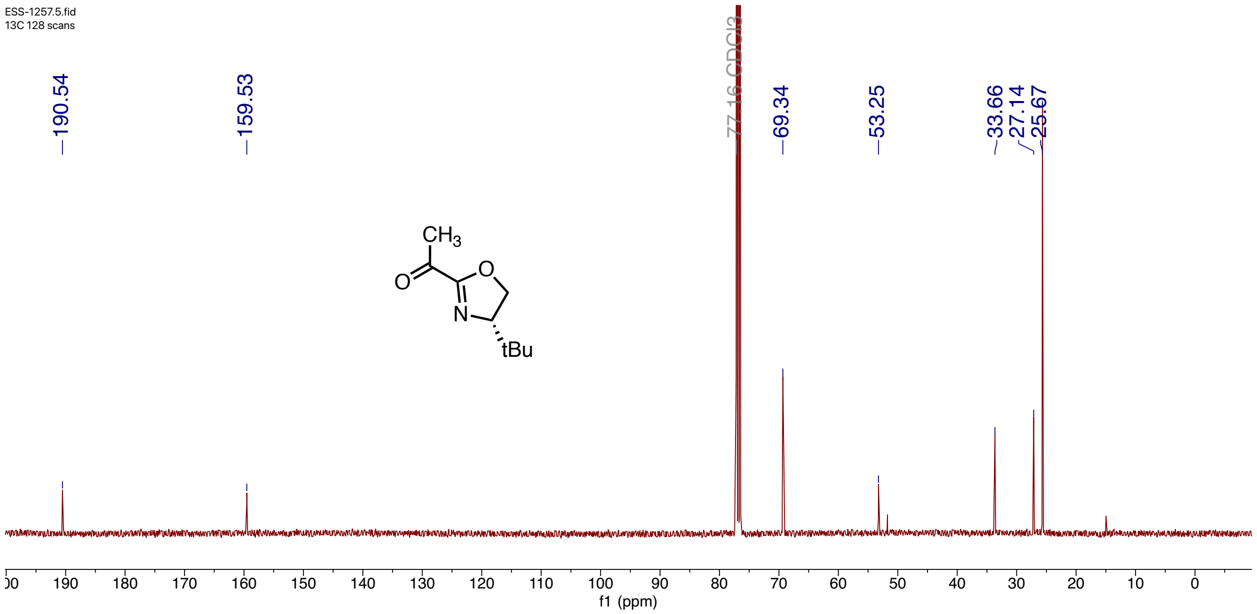

Figure S28. ^{13}C NMR spectrum of crude **3** in chloroform-*d*.

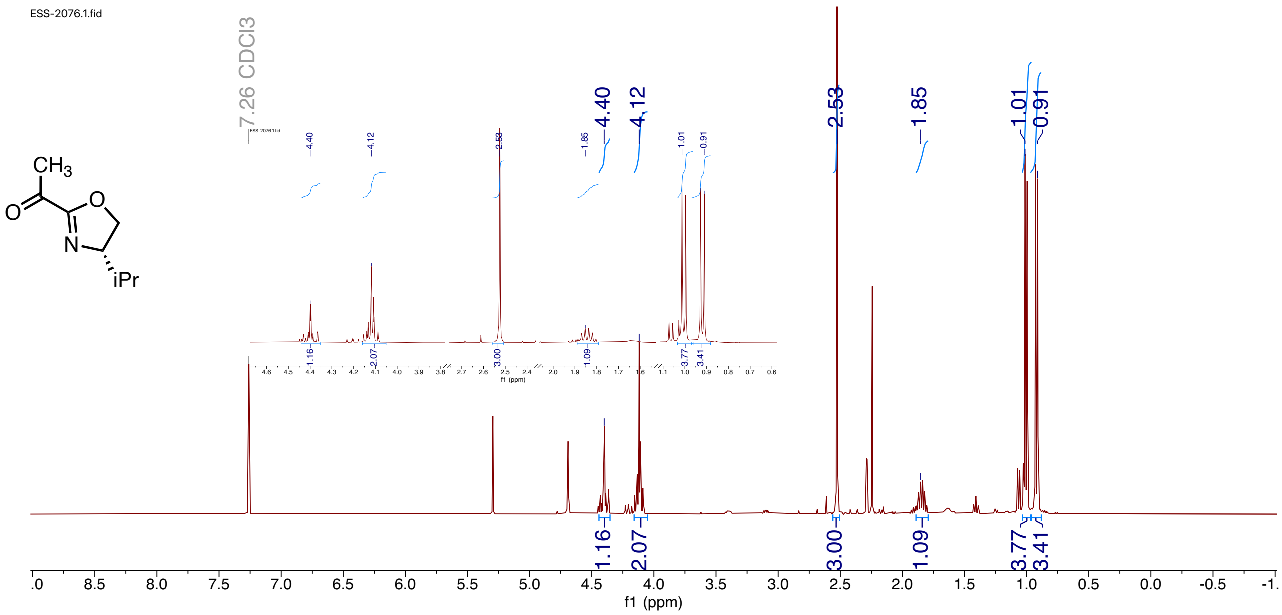

Figure S29. ^{1}H NMR spectrum of crude **4** in chloroform-*d*.

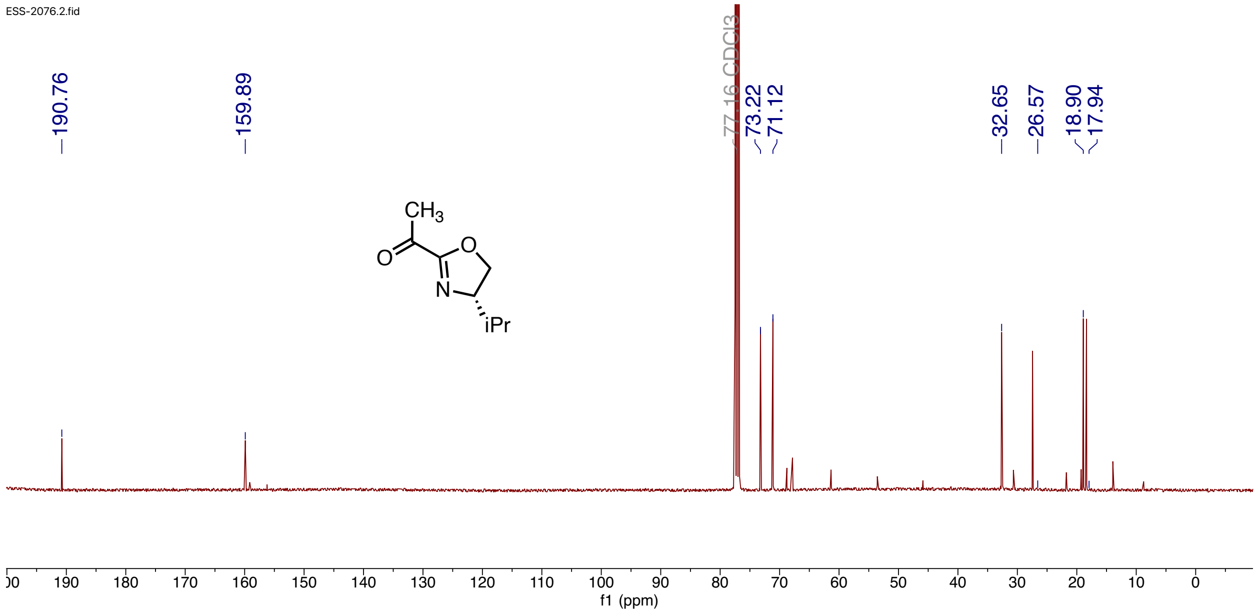

Figure S30. ^{13}C NMR spectrum of crude **4** in chloroform-*d*.

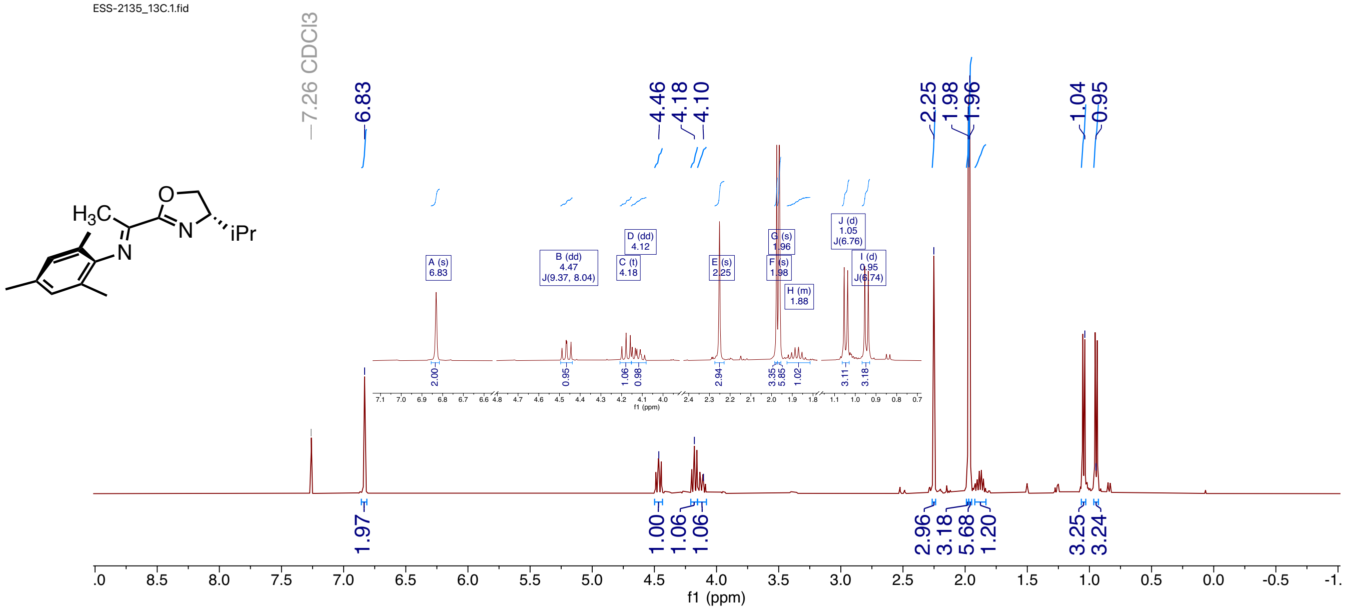

Figure S31. ^{1}H NMR spectrum of **6** in chloroform-*d*.

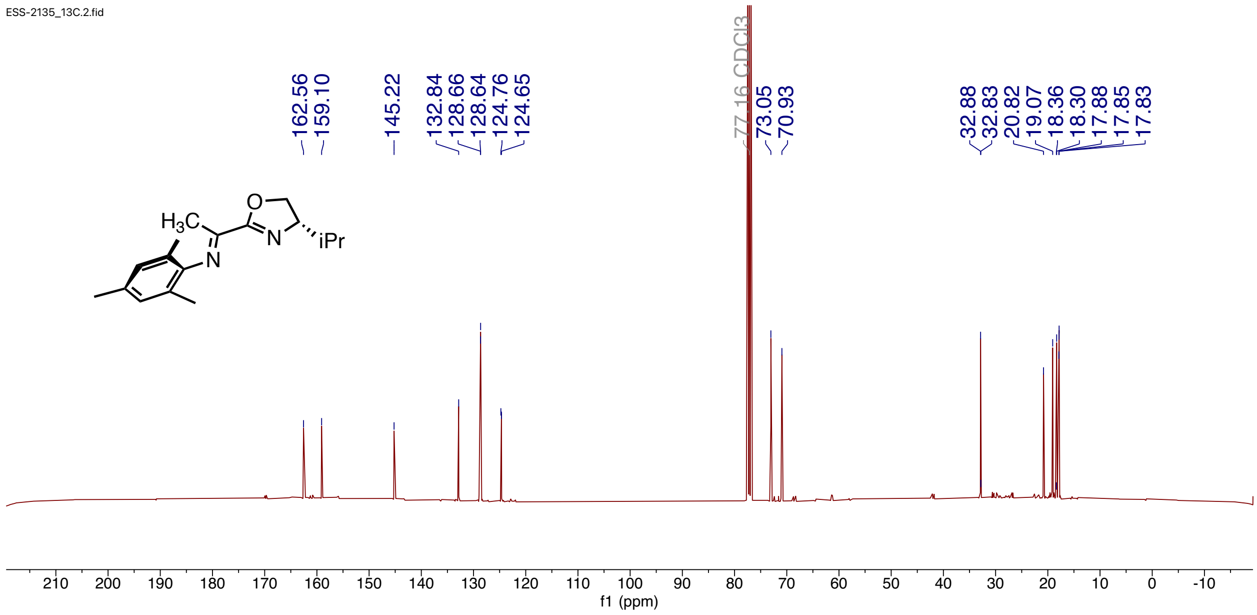

Figure S32. ^{13}C NMR spectrum of **6** in chloroform-*d*.

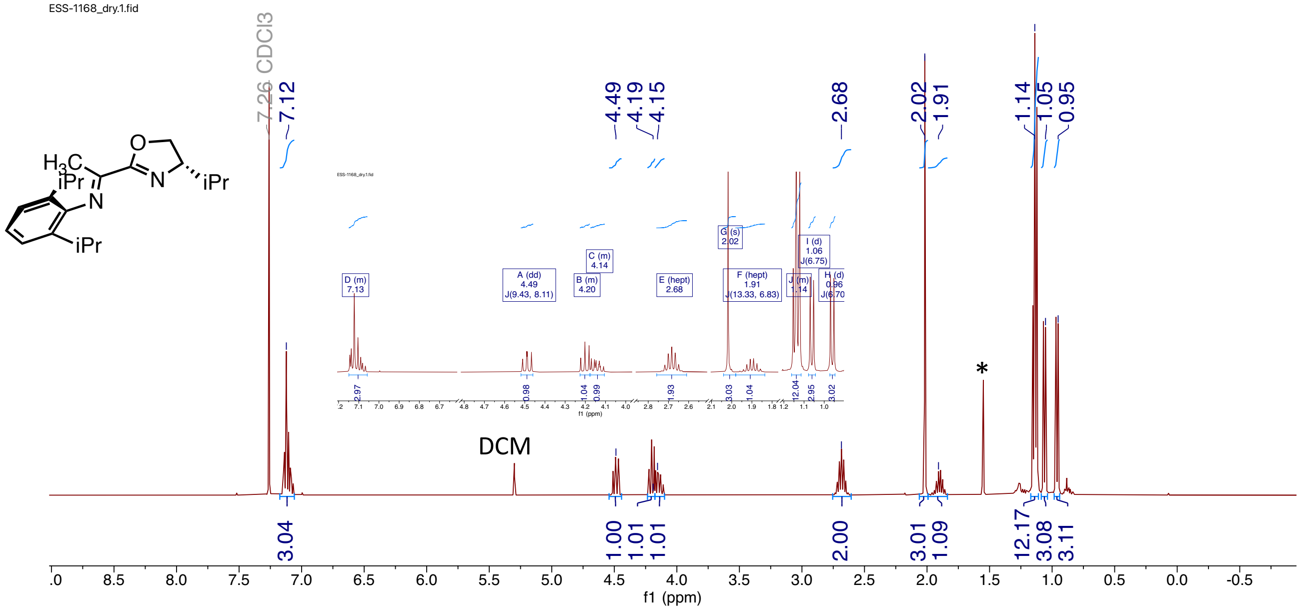

Figure S33. ^{1}H NMR spectrum of **7** in chloroform-*d*. * = water from NMR solvent.

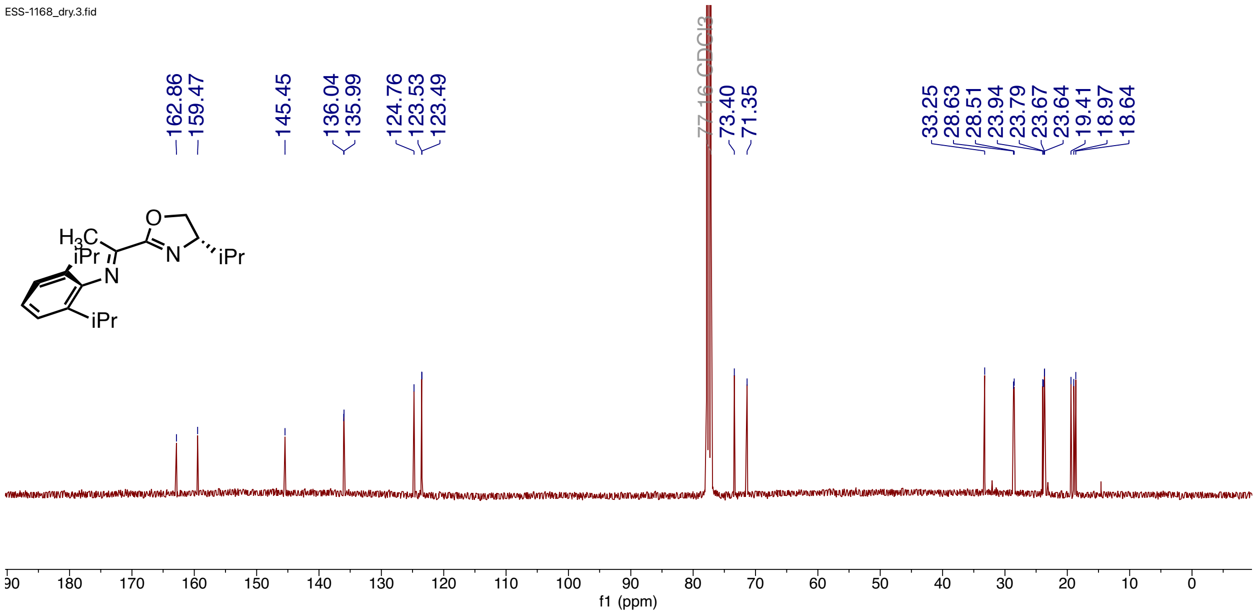

Figure S34. ^{13}C NMR spectrum of **7** in chloroform-*d*.

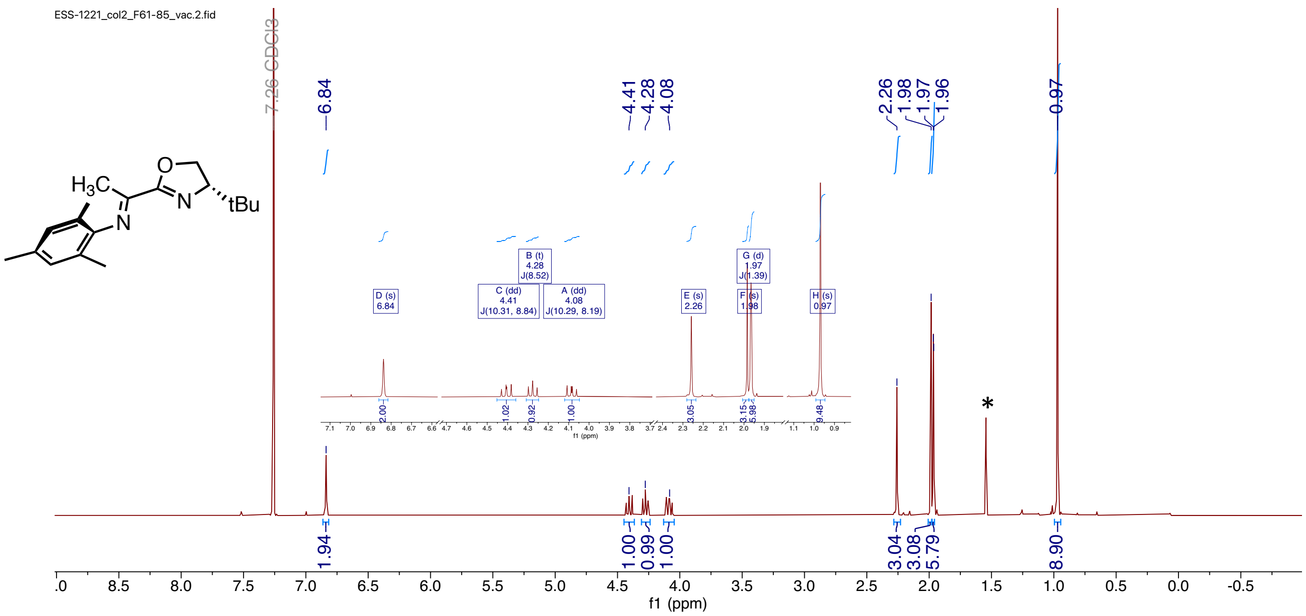

Figure S35. ^{1}H NMR spectrum of **8** in chloroform-*d*. * = water from NMR solvent.

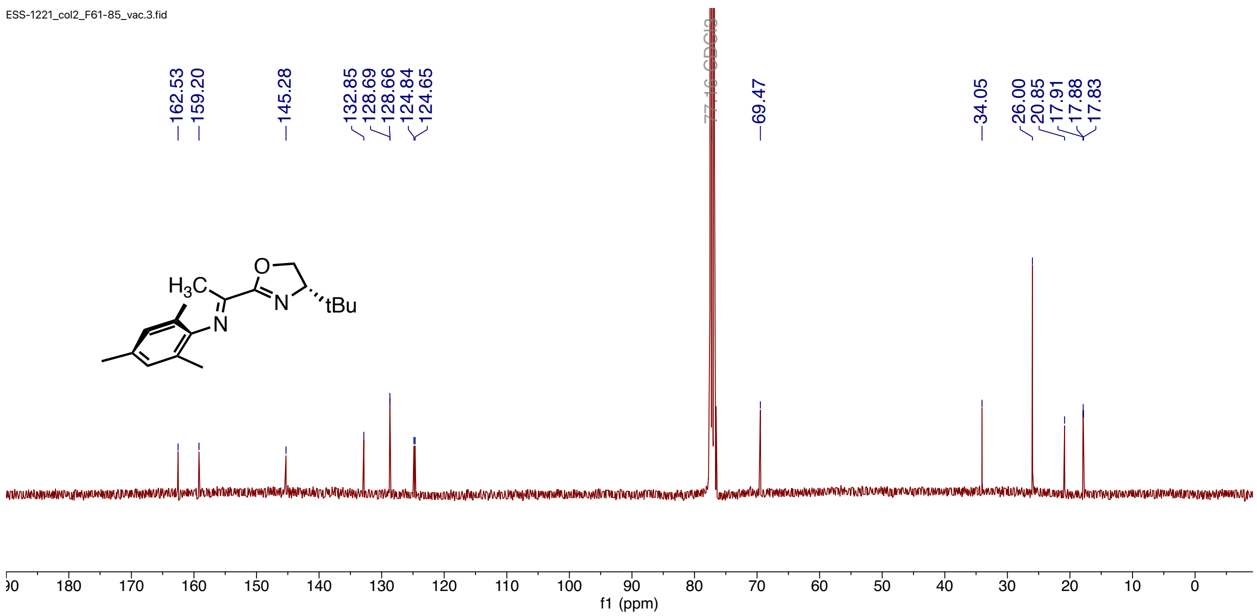

Figure S36. ^{13}C NMR spectrum of **8** in chloroform-*d*.

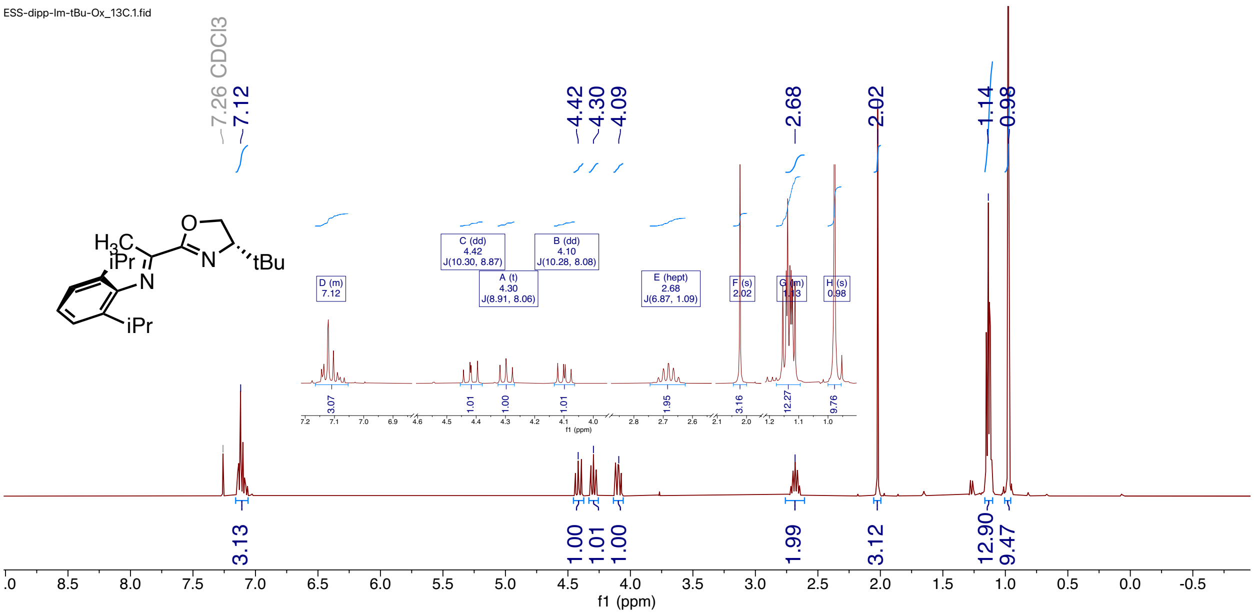

Figure S37. ^{1}H NMR spectrum of **9** in chloroform-*d*.

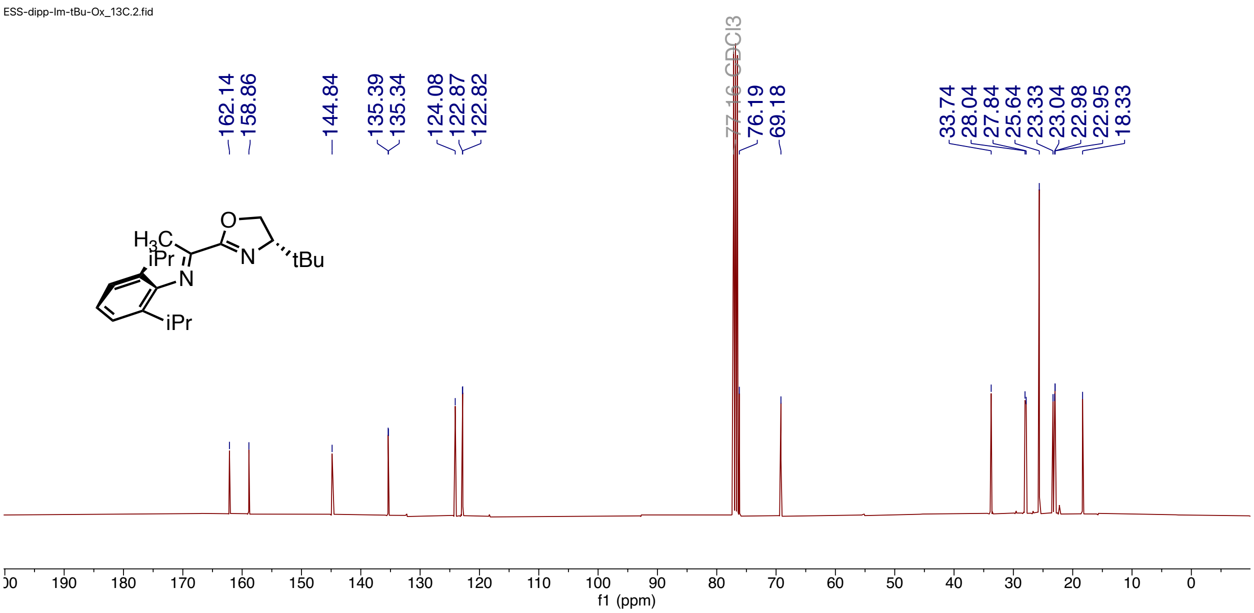

Figure S38. ^{13}C NMR spectrum of **9** in chloroform-*d*.

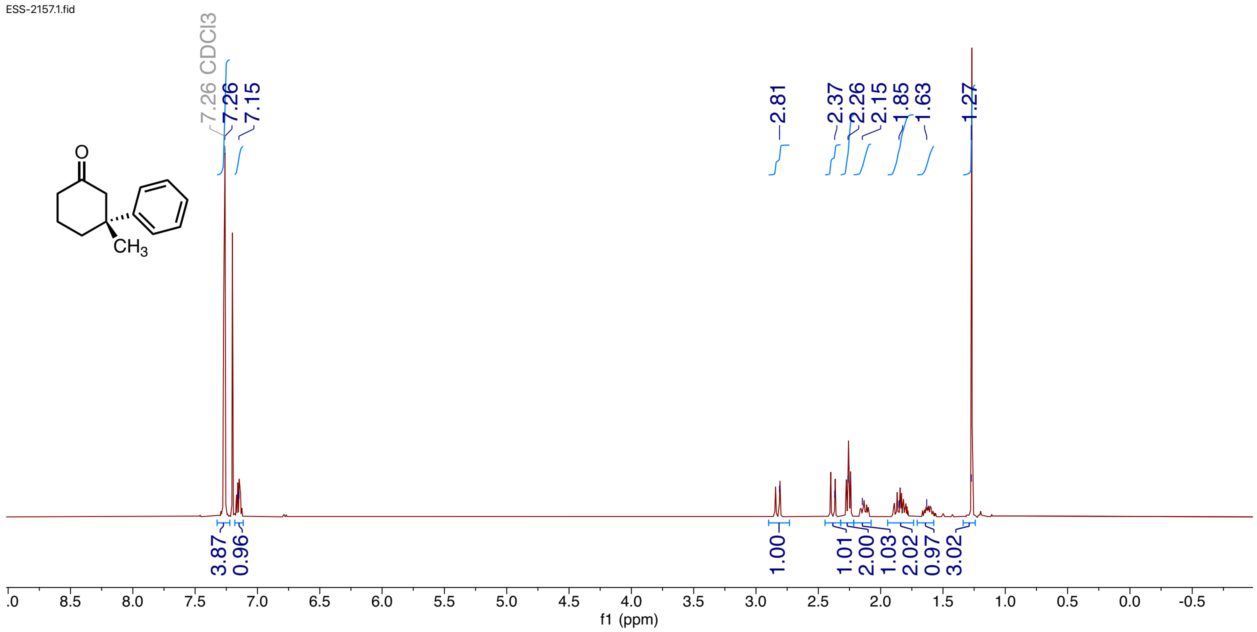

Figure S39. ^{1}H NMR spectrum of **10** in chloroform-*d*.

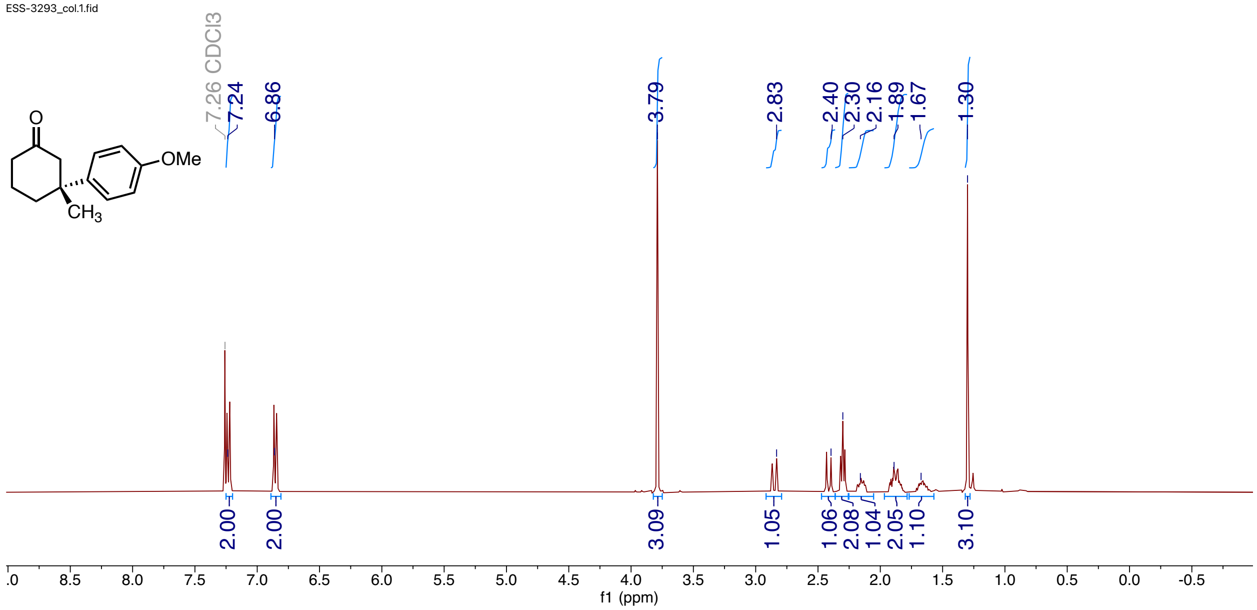

Figure S40. ^{1}H NMR spectrum of **11** in chloroform-*d*.

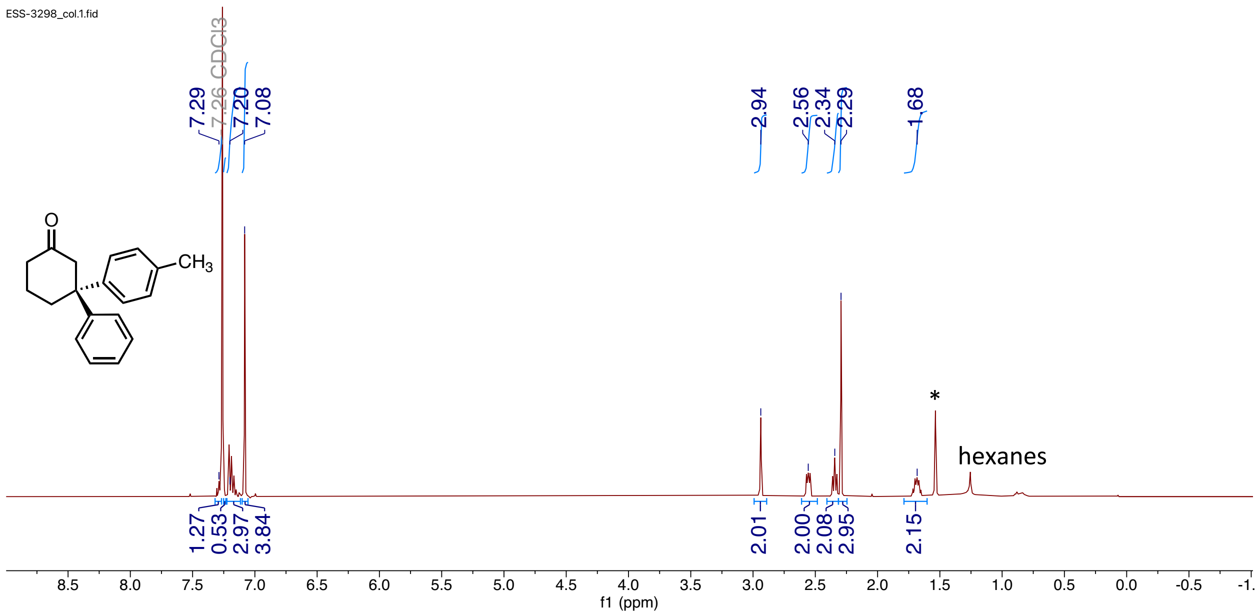

Figure S41. ^{1}H NMR spectrum of **12** in chloroform-*d*. * = water from NMR solvent.

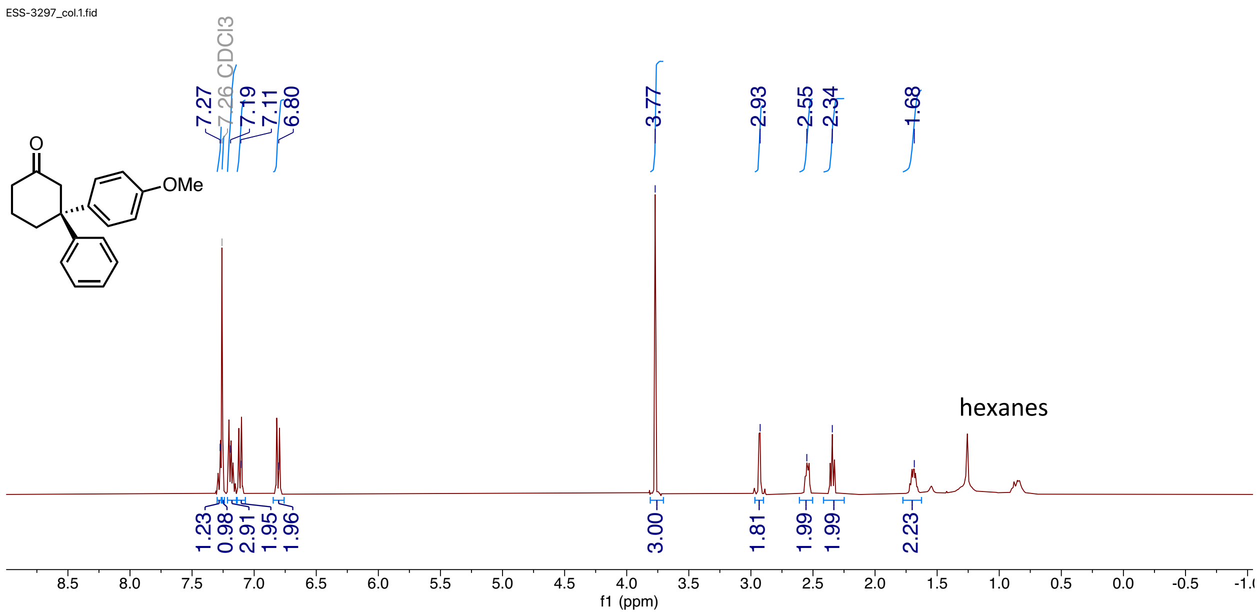

Figure S42. ^{1}H NMR spectrum of **13** in chloroform-*d*.

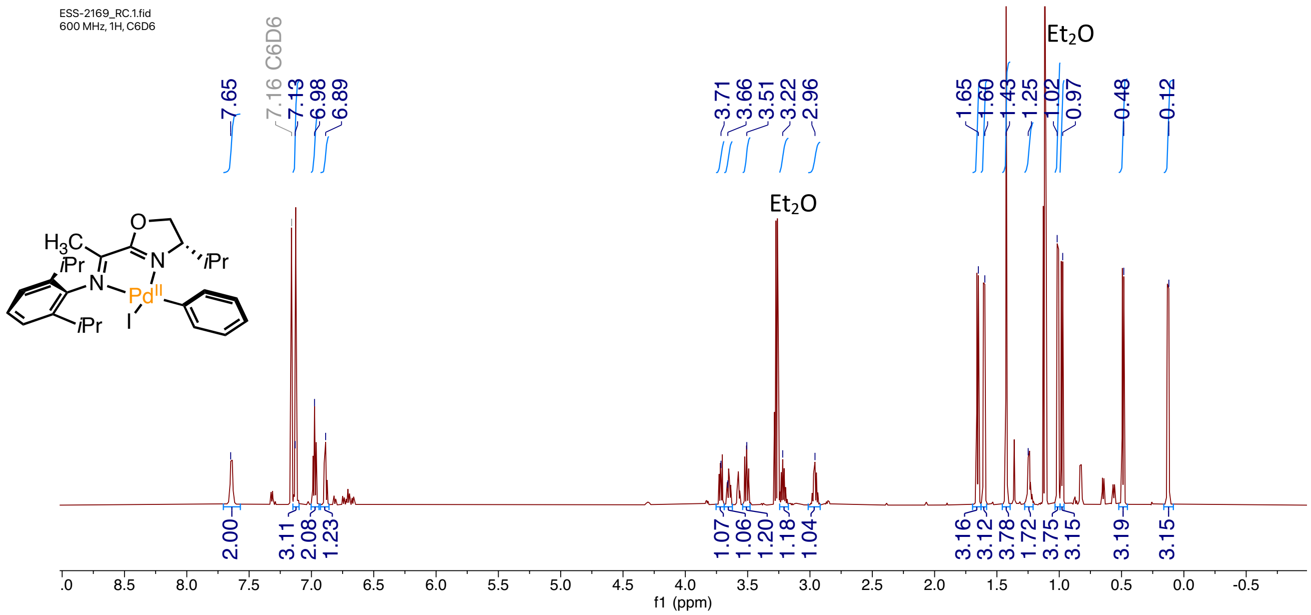

Figure S43. ^{1}H NMR spectrum of **15** in benzene-d_6. Note: peaks for the major diastereomer are picked.

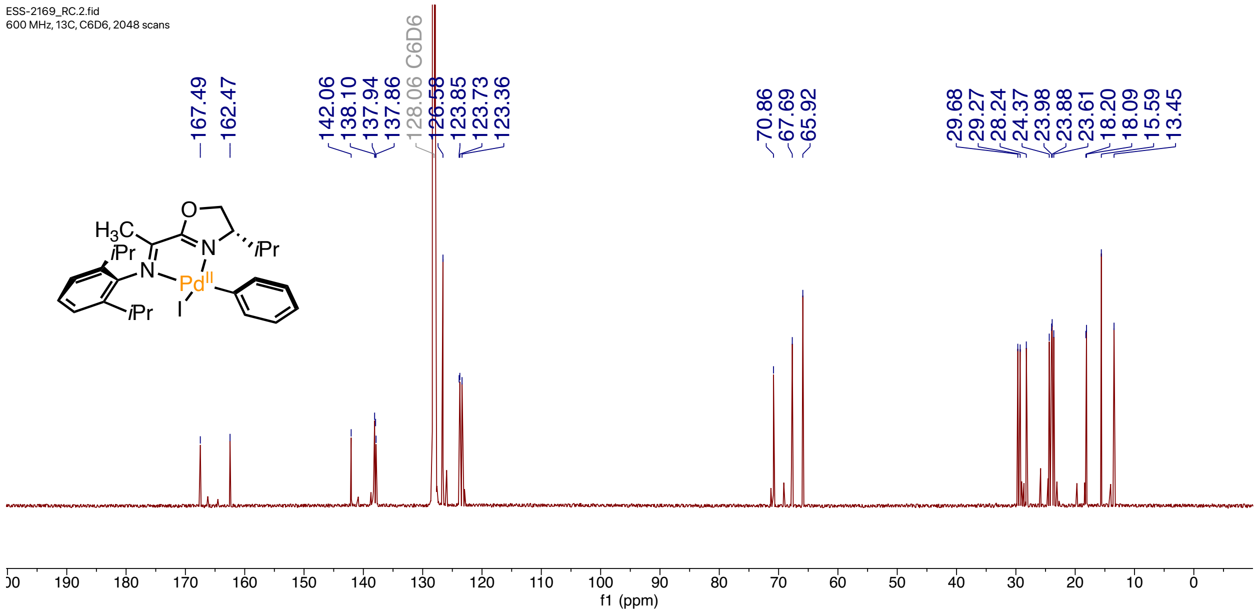

Figure S44. ^{13}C NMR spectrum of **15** in benzene-d_6.

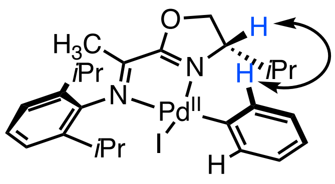

15

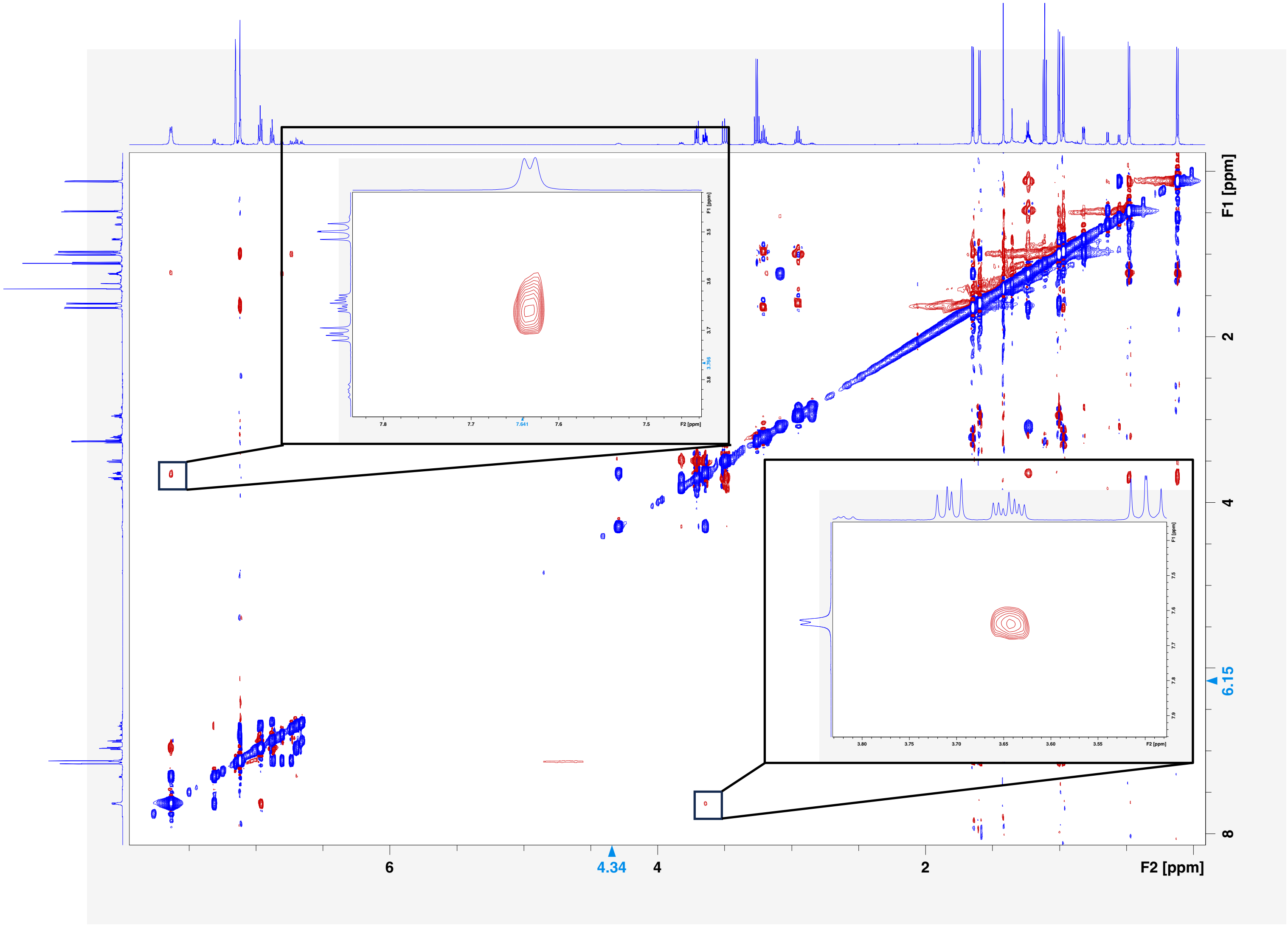

Figure S45. ^{1}H NOESY (600 MHz, C_6D_6, 23°C) of **15**.

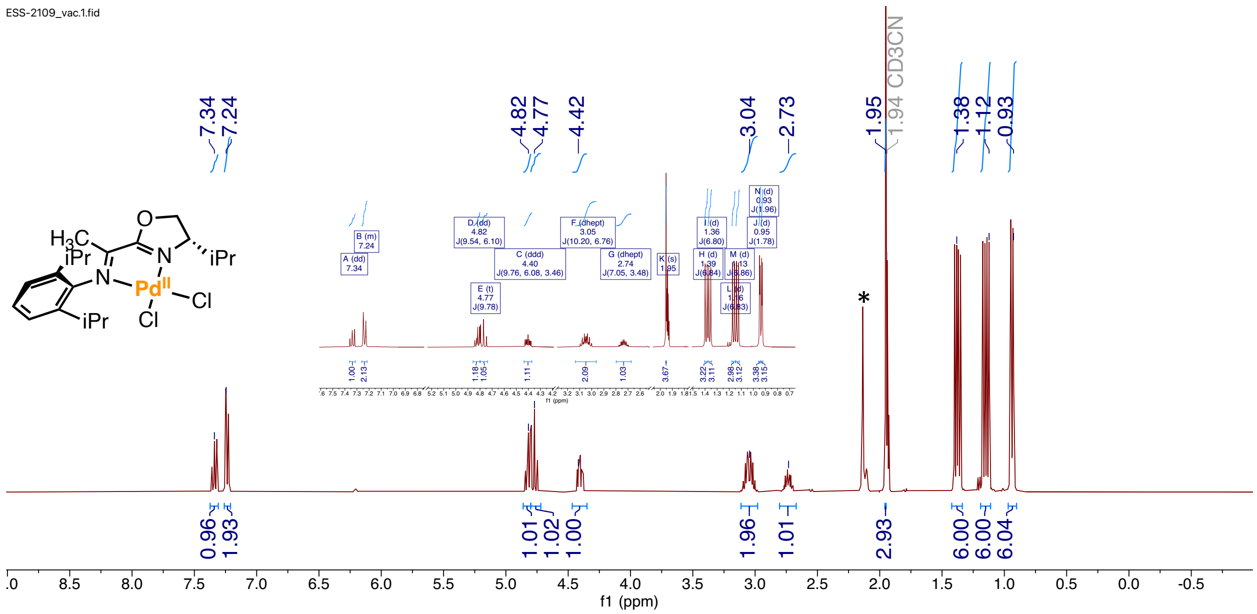

Figure S46. ^{1}H NMR spectrum of **19** in acetonitrile-d_3. * = water from NMR solvent.

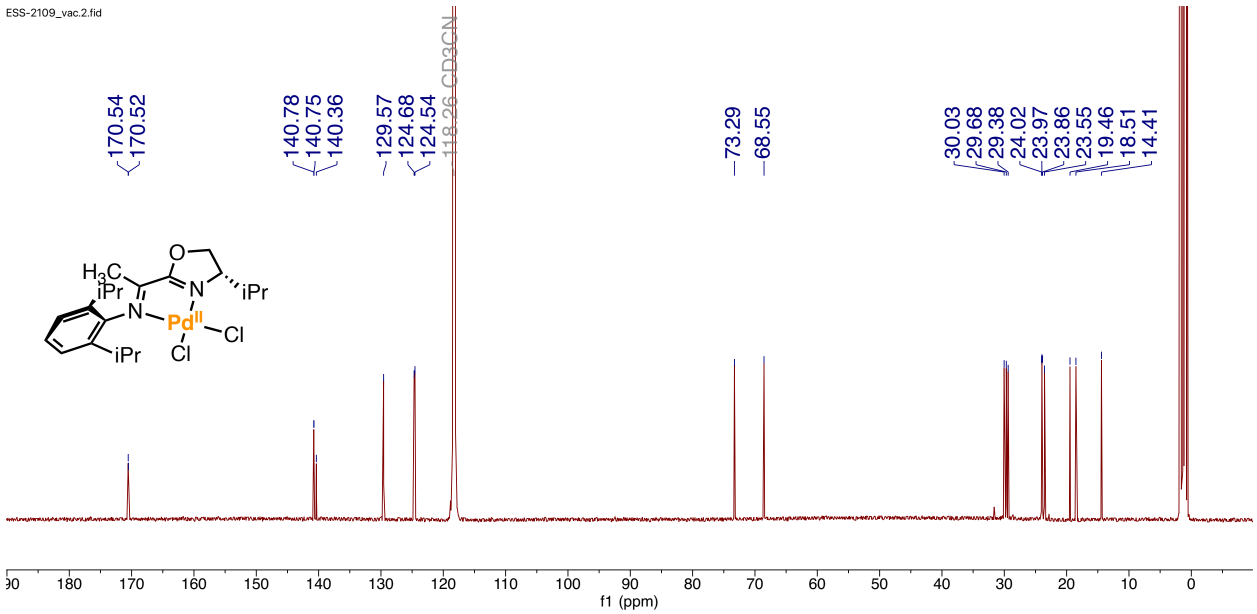

Figure S47. ^{13}C NMR spectrum of **19** in acetonitrile-d_3.

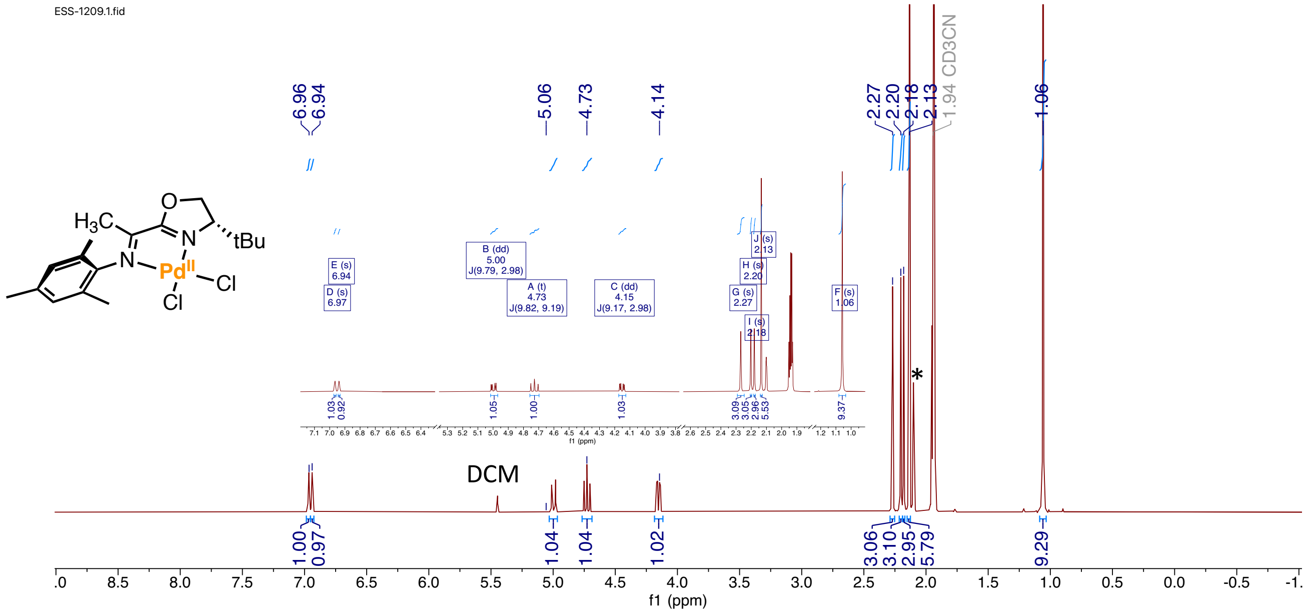

Figure S48. ^{1}H NMR spectrum of **20** in acetonitrile-d_3. * = water from NMR solvent.

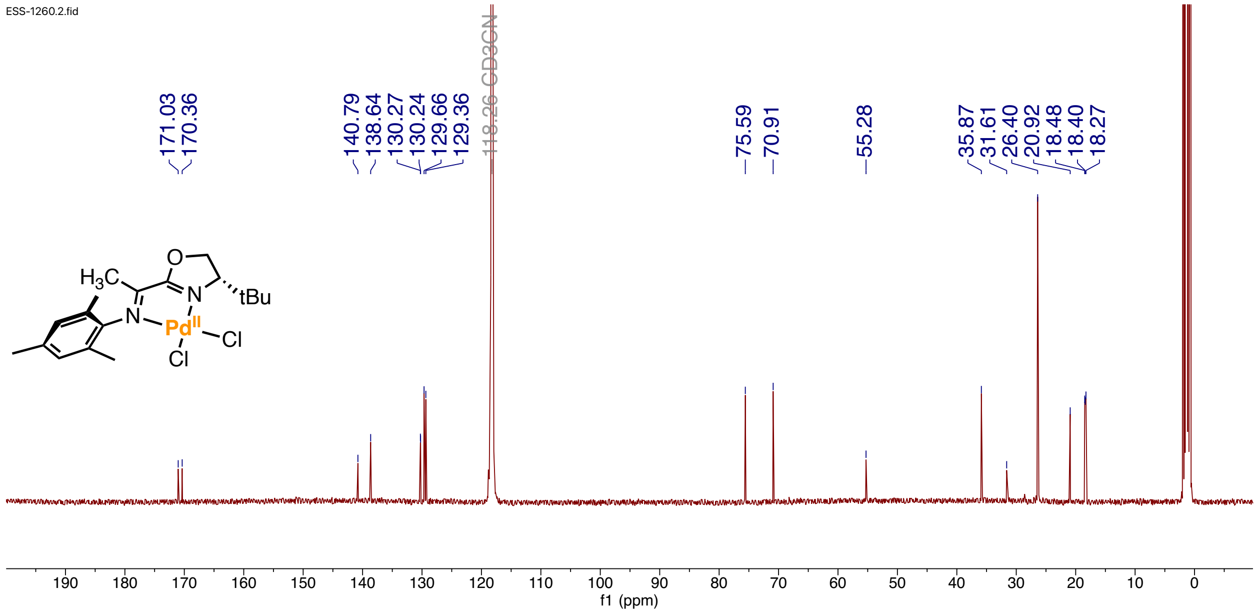

Figure S49. ^{13}C NMR spectrum of **20** in acetonitrile-d_3.

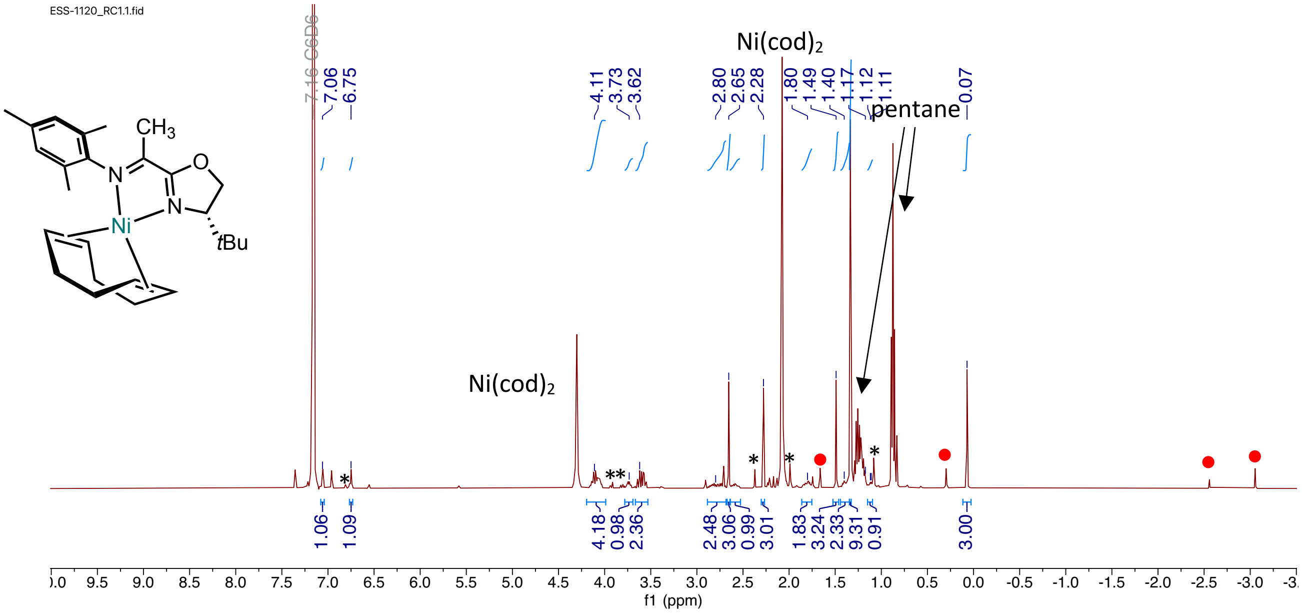

Figure S50. ^{1}H NMR spectrum of **21** in benzene-d_6. Red dots indicate unidentifiable peaks from speciation in solution. * = free ligand.

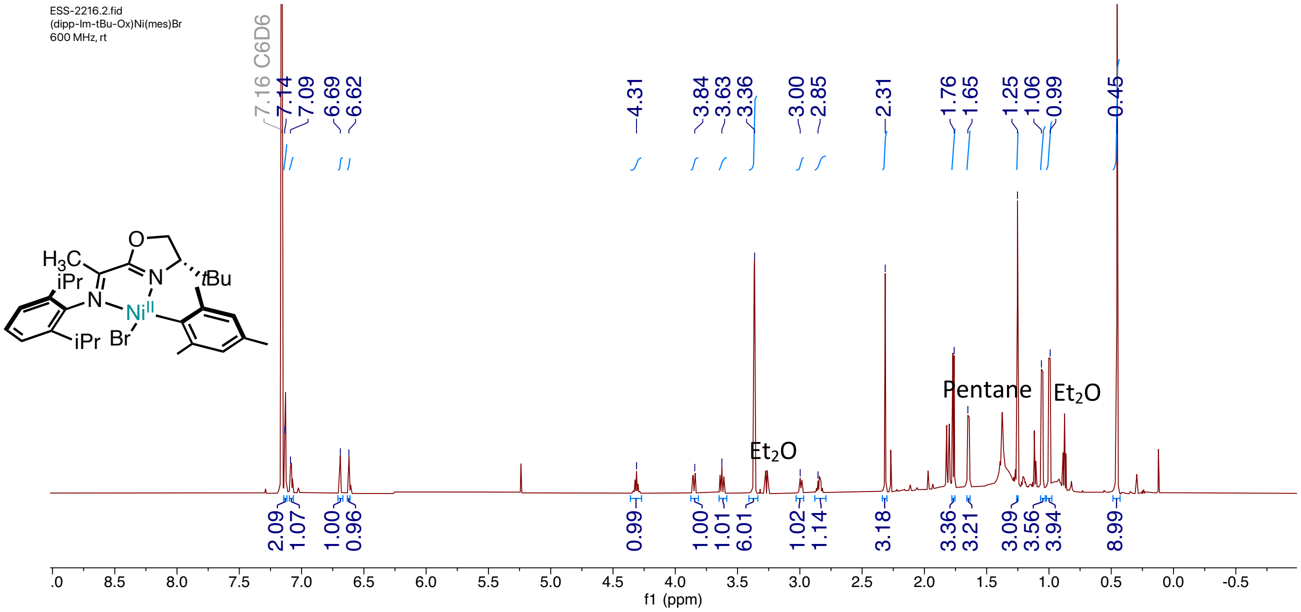

Figure S51. ^{1}H NMR spectrum of **22** in benzene-d_6.

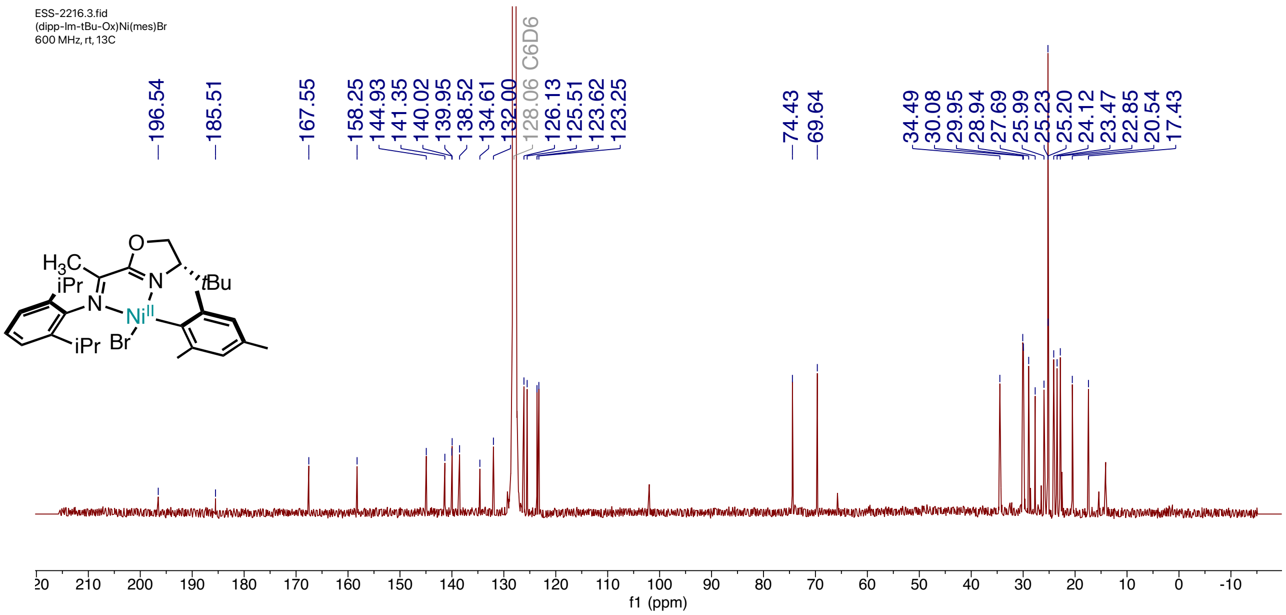

Figure S52. ^{13}C NMR spectrum of **22** in benzene-d_6.

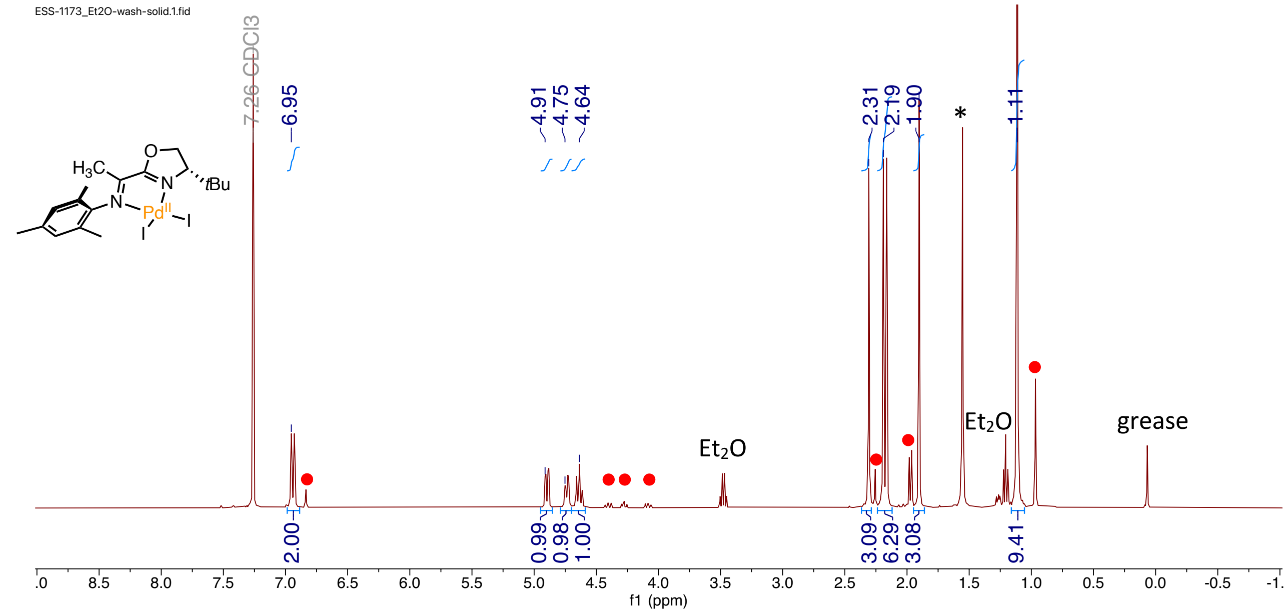

Figure S53. ^{1}H NMR spectrum of **S1** in $CDCl_3$. Red dots indicate free ligand ($^{Mes}Im^{tBu}Ox$); * = water from NMR solvent.

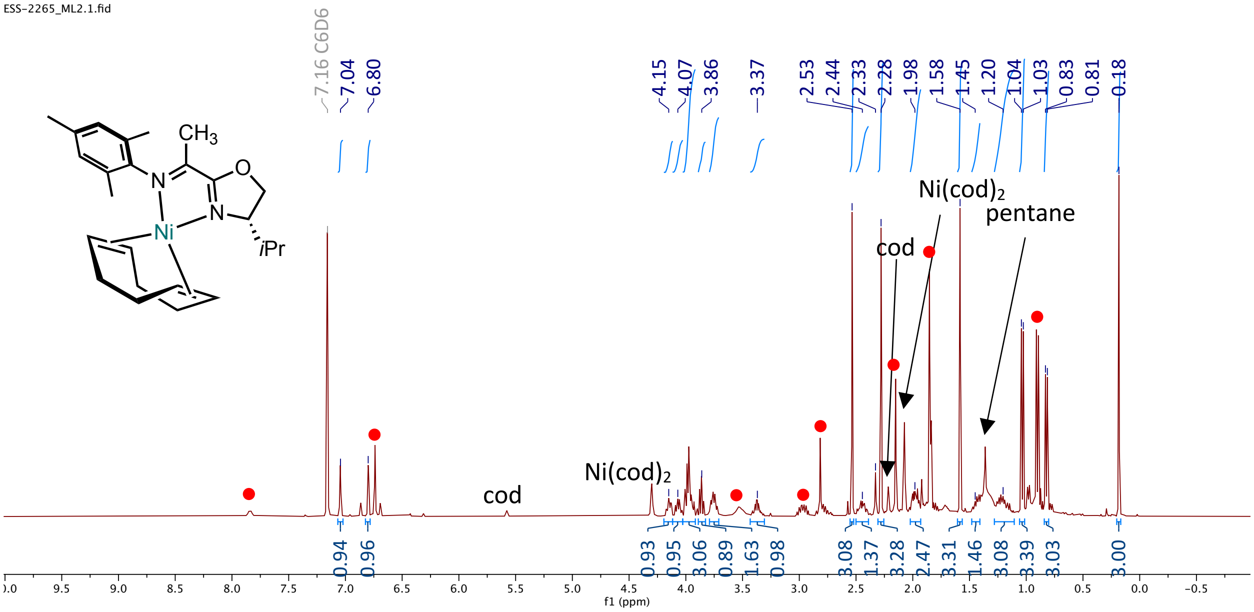

Figure S54. ^{1}H NMR spectrum of **S2** in C_6D_6. Red dots indicate unidentifiable peaks from speciation in solution.

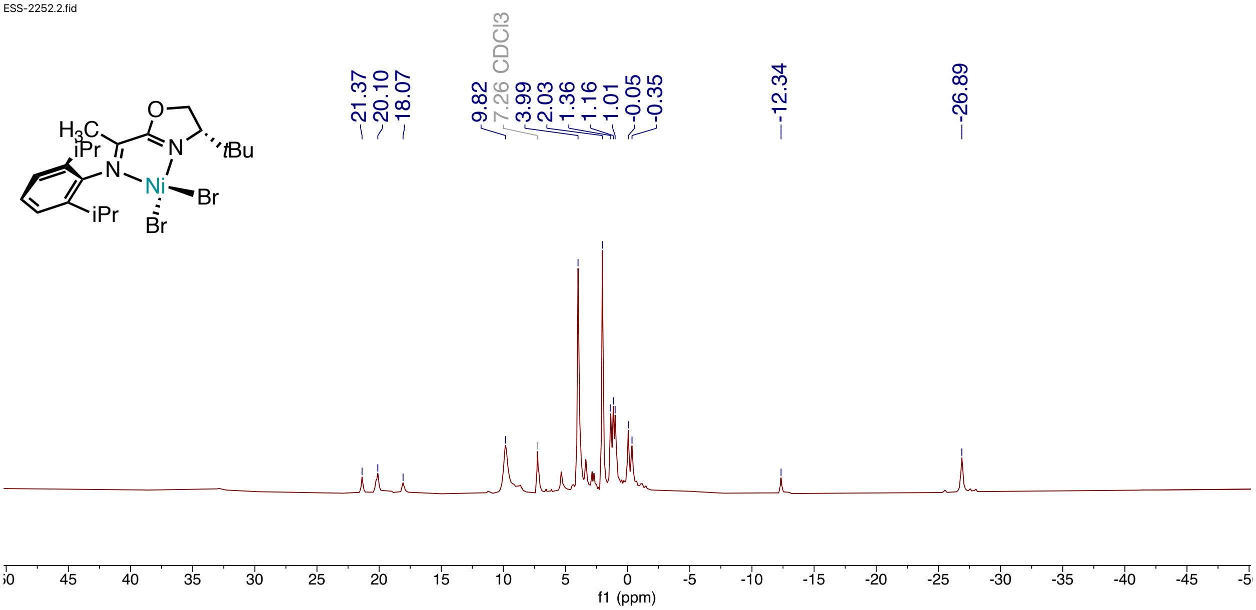

Figure S55. ^{1}H NMR spectrum of **S4** in chloroform-*d*.

11. UV-Vis Spectra

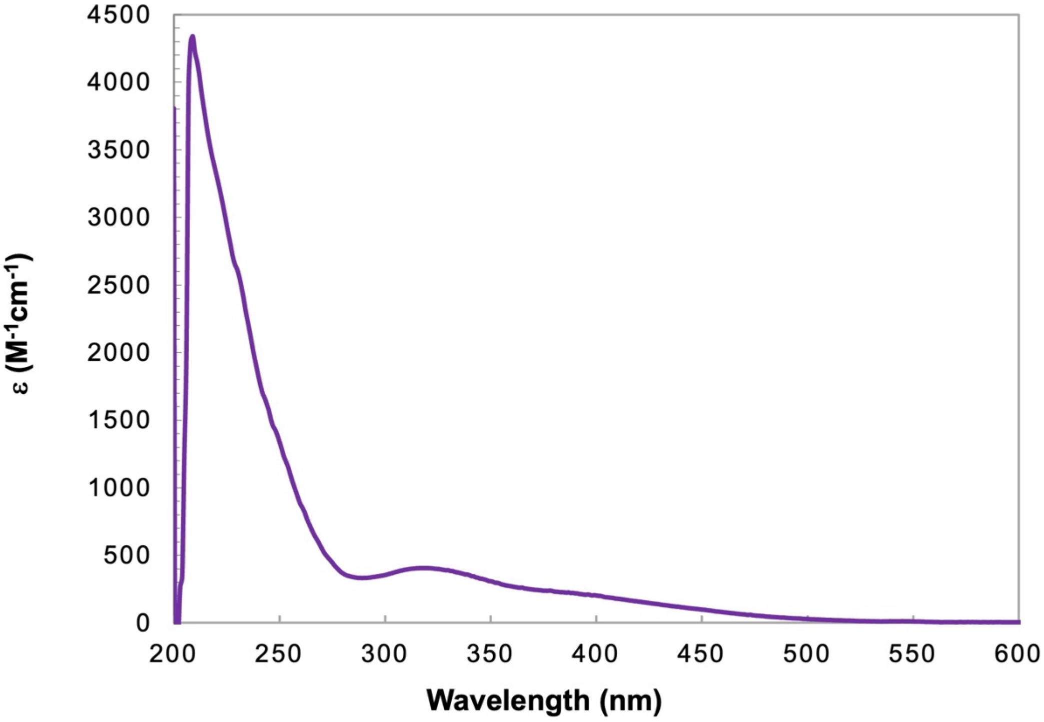

Figure S56. UV-Vis spectrum of ($^{Dipp}Im^{iPr}Ox$)Pd(Ph)I, **15** (THF, 23 °C, 8.7 μM).

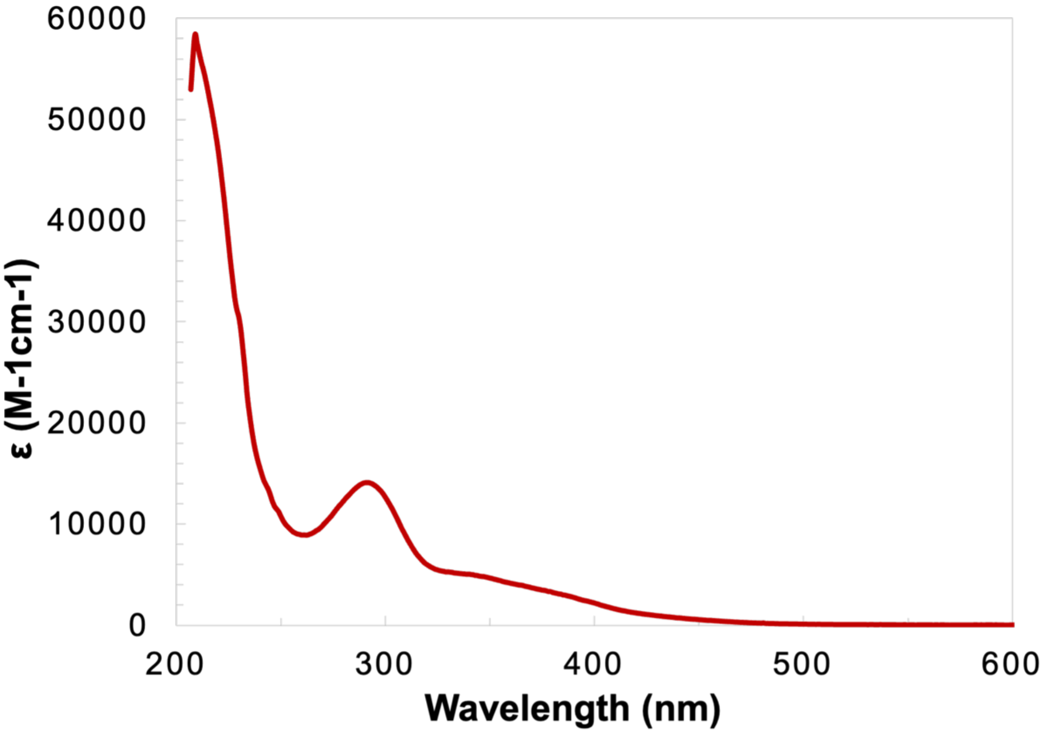

Figure S57. Us-Vis spectrum of ($^{Dipp}Im^{iPr}Ox$)$PdCl_2$, **19** (THF, 23 °C, 8.0μM).

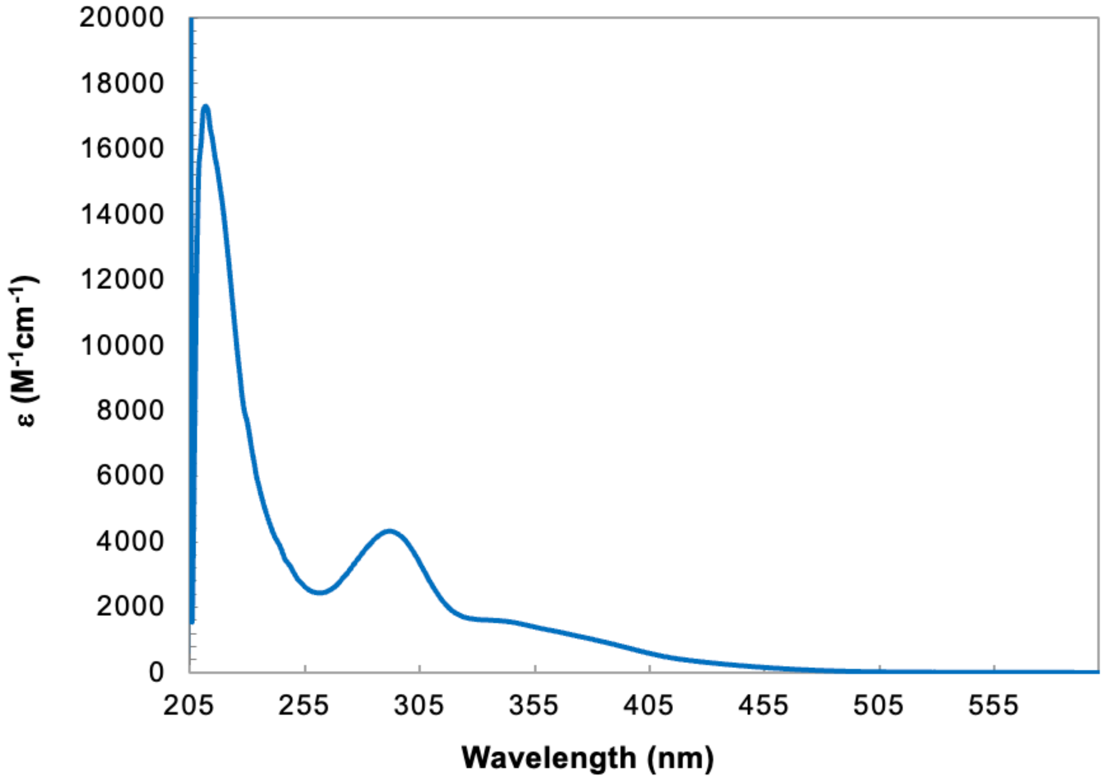

Figure S58. UV-Vis spectrum of (^{Mes}Im^{tBu}Ox)PdCl$_2$, **20** (THF, 23 °C, 5.8 μM).

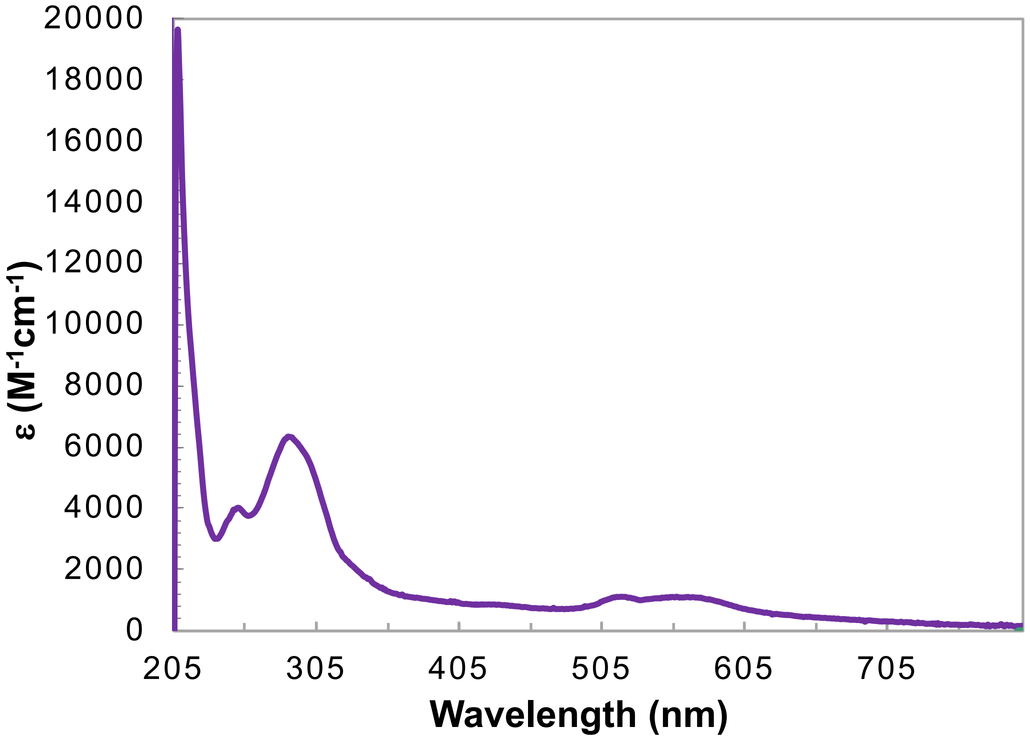

Figure S59. UV-Vis spectrum of (^{Dipp}Im^{tBu}Ox)Ni(Mes)Br, **22** (THF, 23 °C, 8.7 μM).

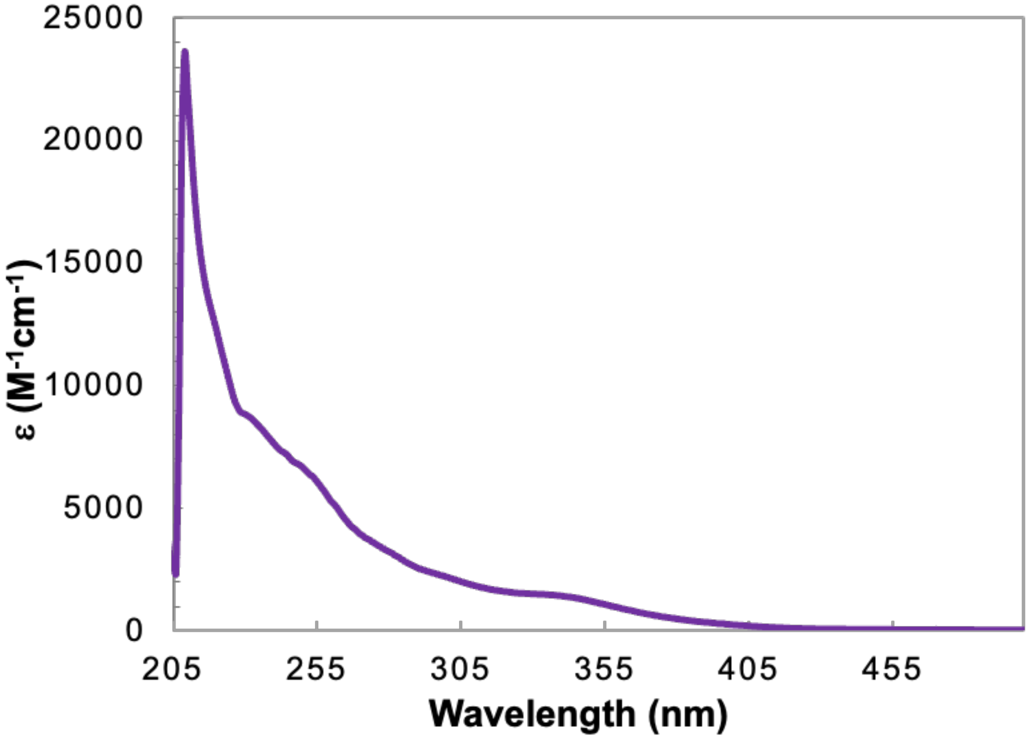

Figure S60. UV-Vis spectrum of (^{Dipp}Im^{tBu}Ox)NiBr$_2$, **S4** (THF, 23 °C, 108 μM).

12. Cyclic Voltammetry Data

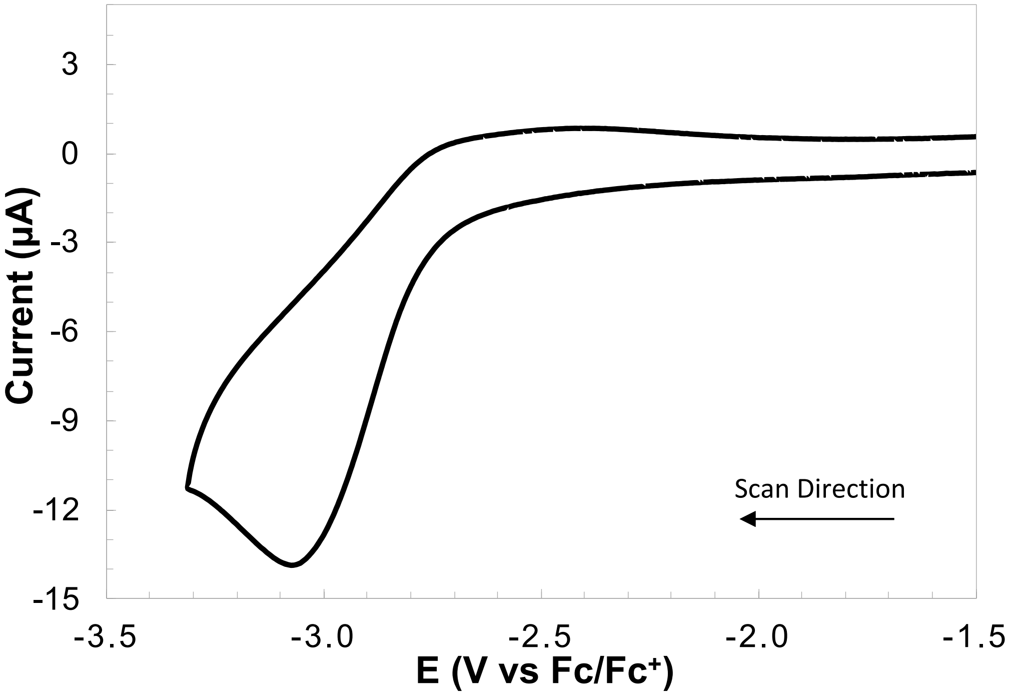

Figure S61. CV of $^{Mes}Im^{tBu}Ox$, **8**. $E_{p,\ red}$ = -3.07 V (vs Fc/Fc$^+$). Solvent = THF, temperature = 298 K, scan rate = 100 mV/s, [$^{Mes}Im^{tBu}Ox$] = 1.0 mM, [$TBAPF_6$] = 100 mM.

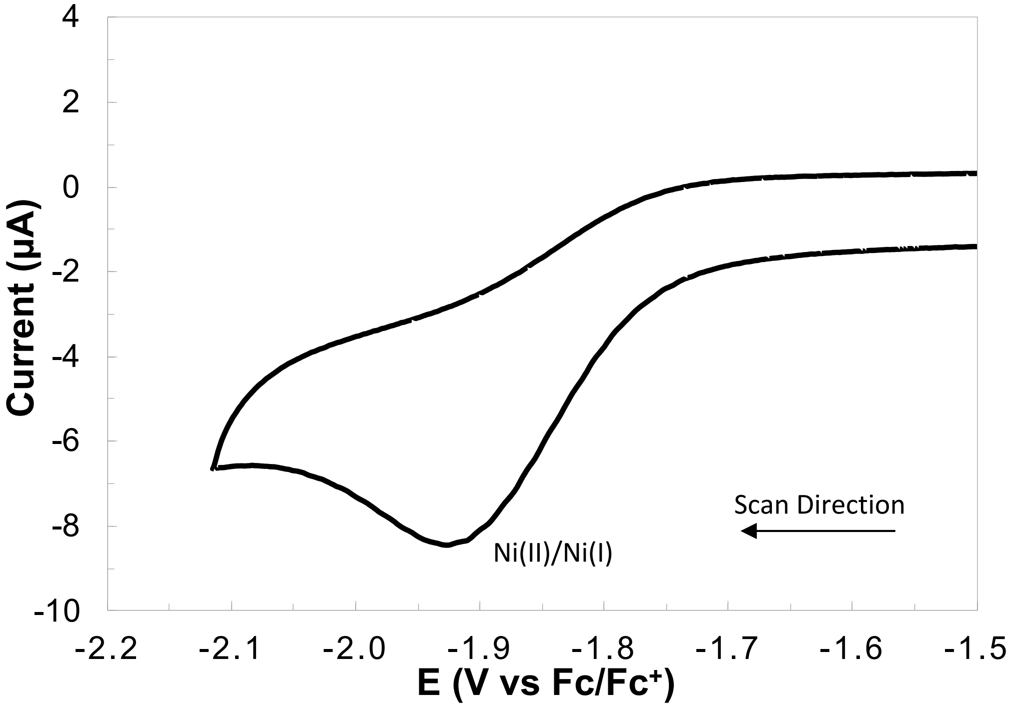

Figure S62. CV of ($^{Dipp}Im^{tBu}Ox$)Ni(Mes)Br, **22**. $E_{p,\ red}$ = -1.93 V (vs Fc/Fc$^+$). Solvent = THF, temperature = 298 K, scan rate = 100 mV/s, [($^{Dipp}Im^{tBu}Ox$)Ni(mes)Br] = 1.0 mM, [$TBAPF_6$] = 100 mM.

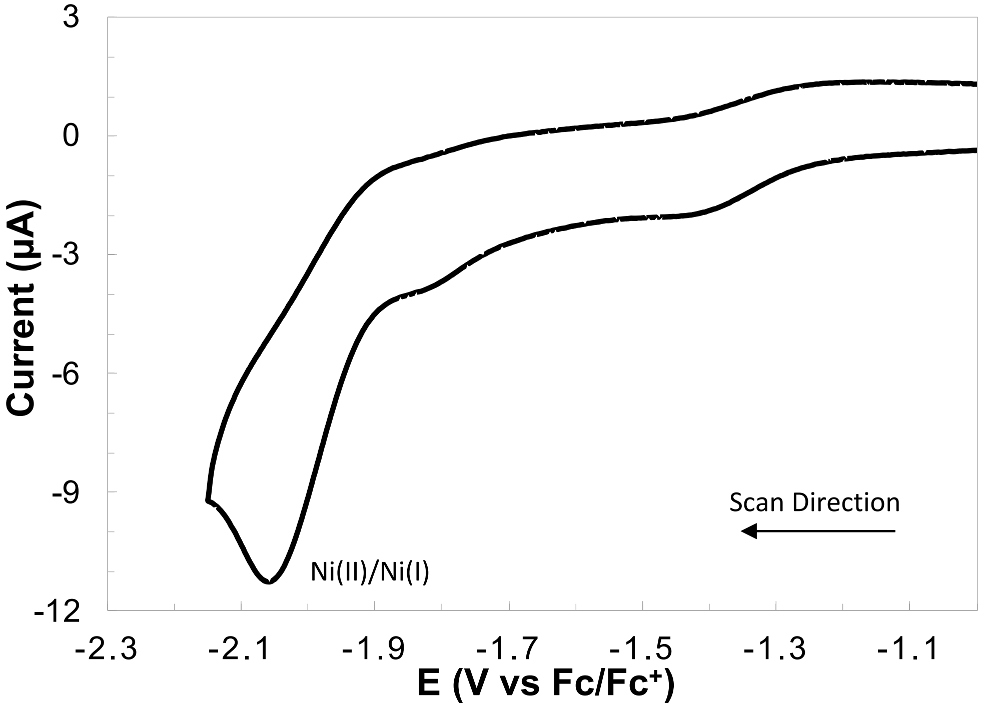

Figure S63. CV of (^{iPr}BiOx)Ni(Mes)Br. $E_{p,\ red}$ = -2.06 V (vs Fc/Fc^{+}). Solvent = THF, temperature = 298 K, scan rate = 100 mV/s, [(^{Dipp}Im^{tBu}Ox)Ni(mes)Br] = 1.0 mM, [TBAPF$_6$] = 100 mM.

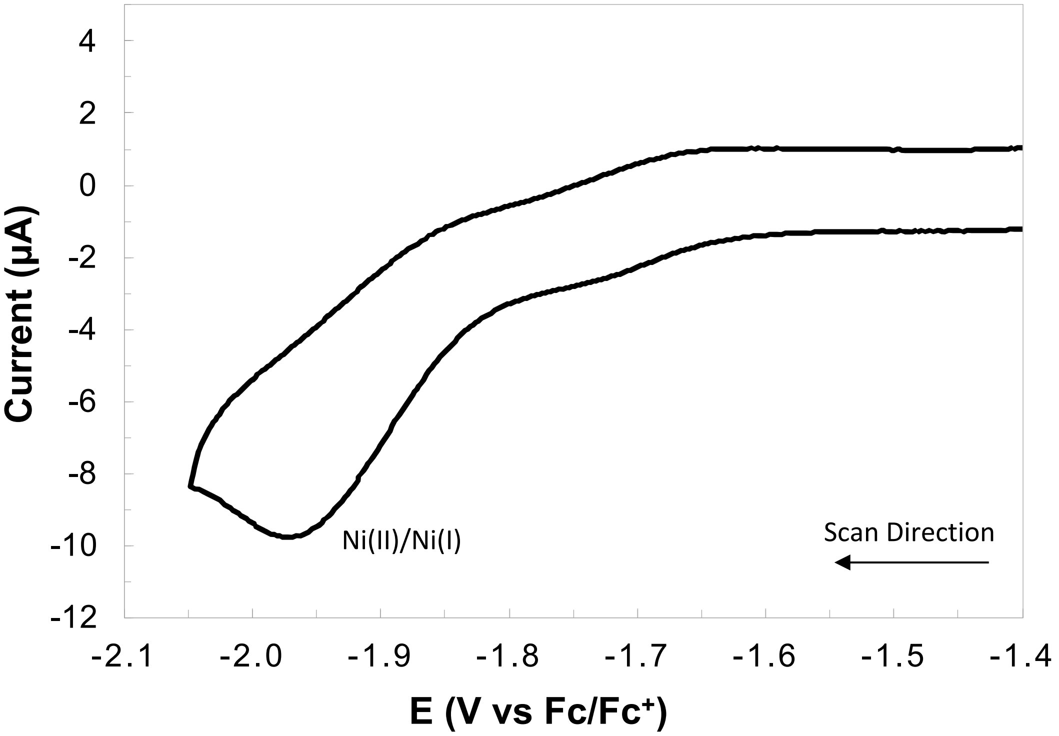

Figure S64. CV of (^{tBu}PyOx)Ni(Mes)Br. $E_{p,\ red}$ = -1.97 V (vs Fc/Fc^{+}). Solvent = THF, temperature = 298 K, scan rate = 100 mV/s, [(^{tBu}PyOx)Ni(mes)Br] = 1.0 mM, [TBAPF$_6$] = 100 mM.

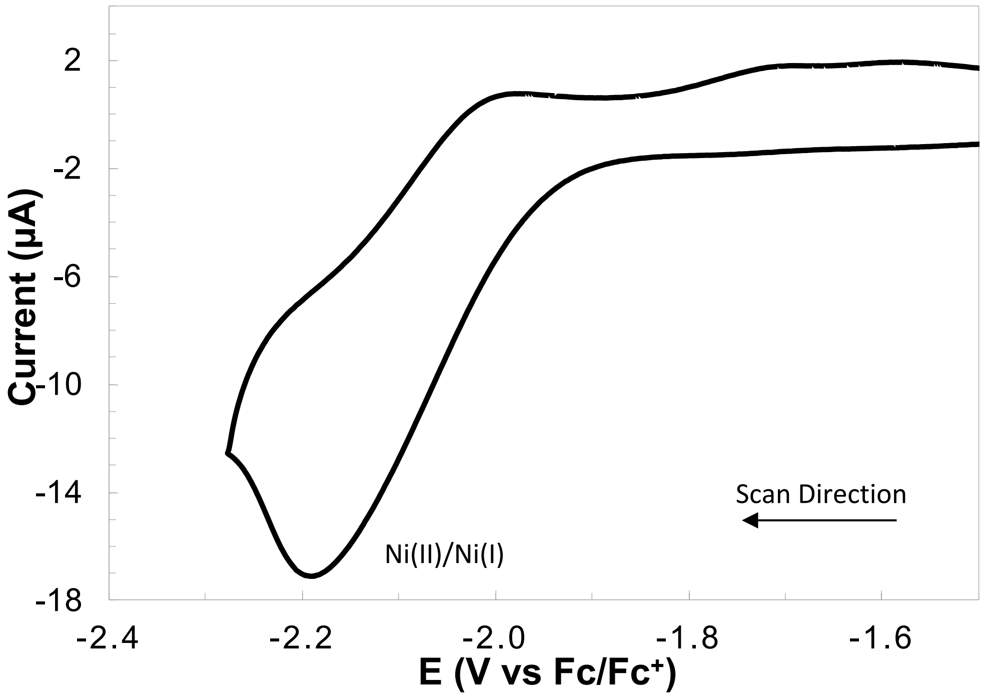

Figure S65. CV of (bpy)Ni(Mes)Br. $E_{p,\ red}$ = -2.19 V (vs Fc/Fc^{+}). Solvent = THF, temperature = 298 K, scan rate = 100 mV/s, [(^{tBu}PyOx)Ni(mes)Br] = 1.0 mM, [$TBAPF_6$] = 100 mM.

13. EPR Data

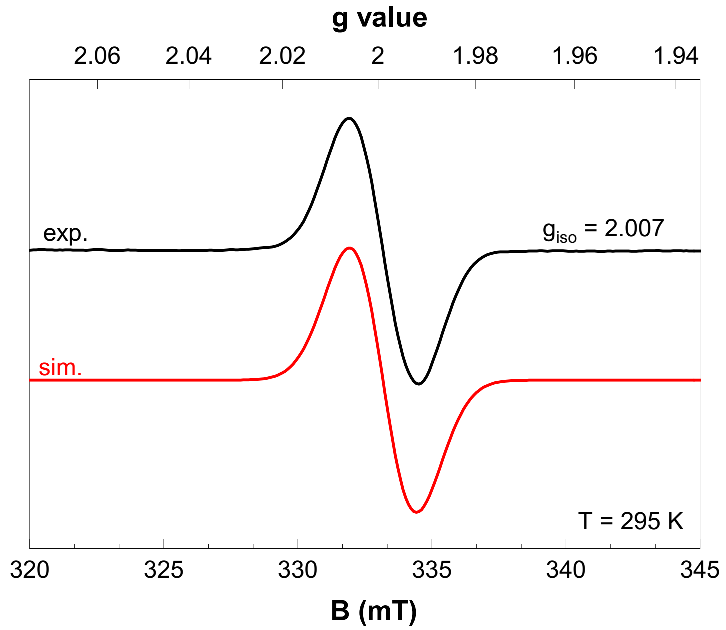

Figure S66. X-band EPR spectrum of KC_8 reduction of **22** (assigned **23**). Temperature = 295 K, solvent = THF, microwave frequency = 9.363381 GHz, power = .4743 mW, modulation amplitude = 10.0 G, modulation frequency = 100 kHz. The simulation was performed using Xepr software. The simulation spectrum (red) uses the following parameters: g_{iso} = 2.007.

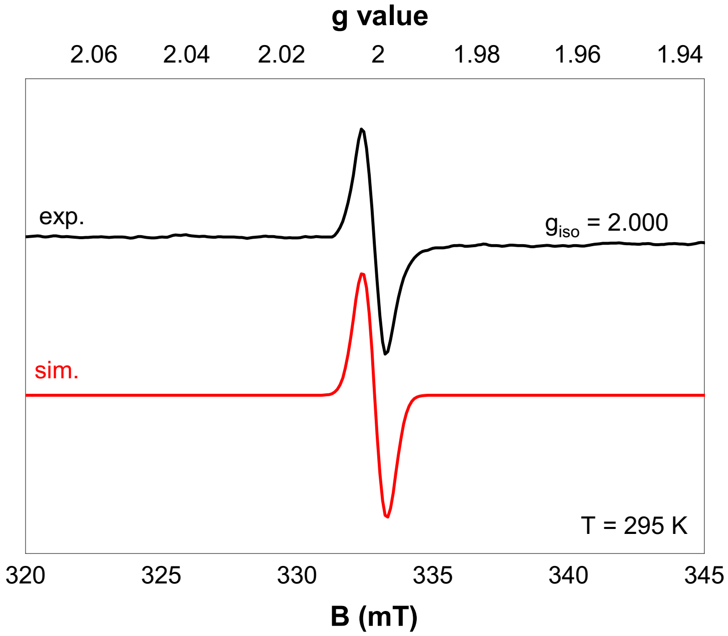

Figure S67. X-band EPR spectrum of KC_8 reduction of **9** (assigned **S3**). Temperature = 295 K, solvent = THF, microwave frequency = 9.374713 GHz, power = .4743 mW, modulation amplitude = 10.0 G, modulation frequency = 100 kHz. The simulation was performed using Xepr software. The simulation spectrum (red) uses the following parameters: g_{iso} = 2.000.

14. FT-IR Spectra

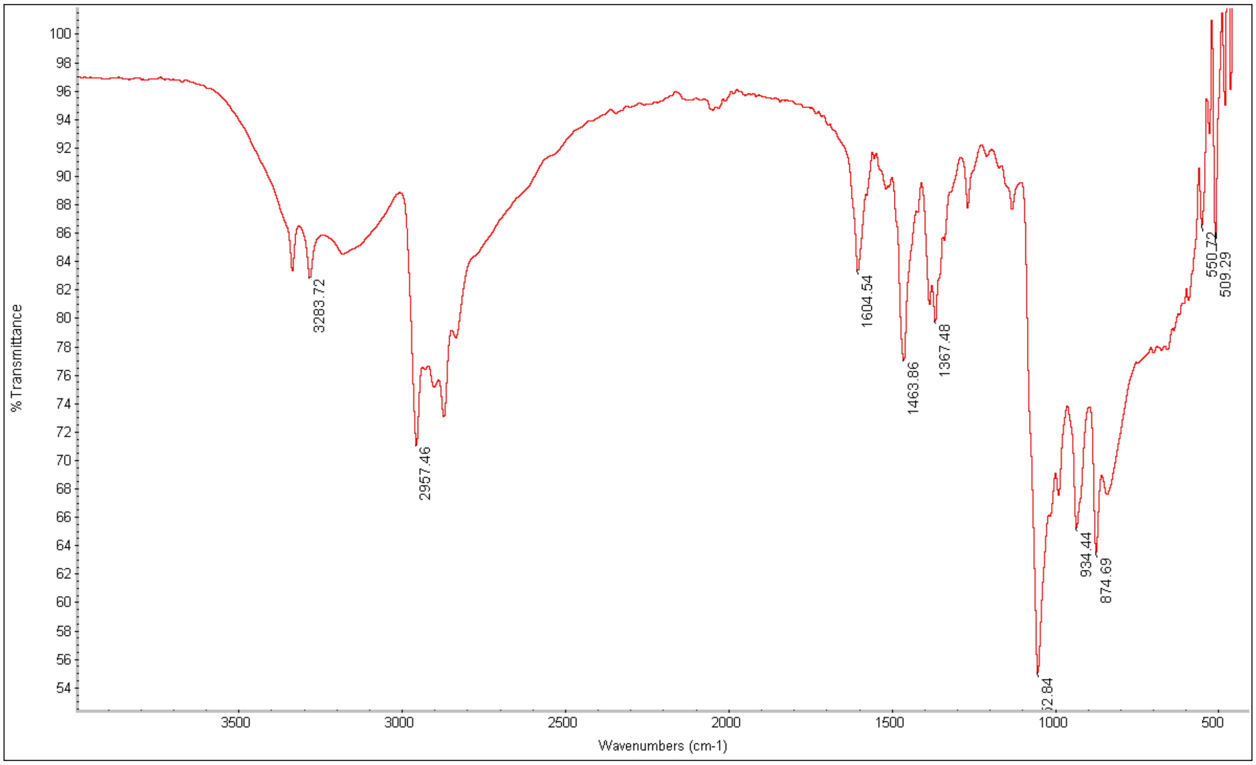

Figure S68. FT-IR spectrum of 1-((*S*)-4-(*tert*-butyl)-4,5-dihydrooxazol-2-yl)ethan-1-ol, **1**.

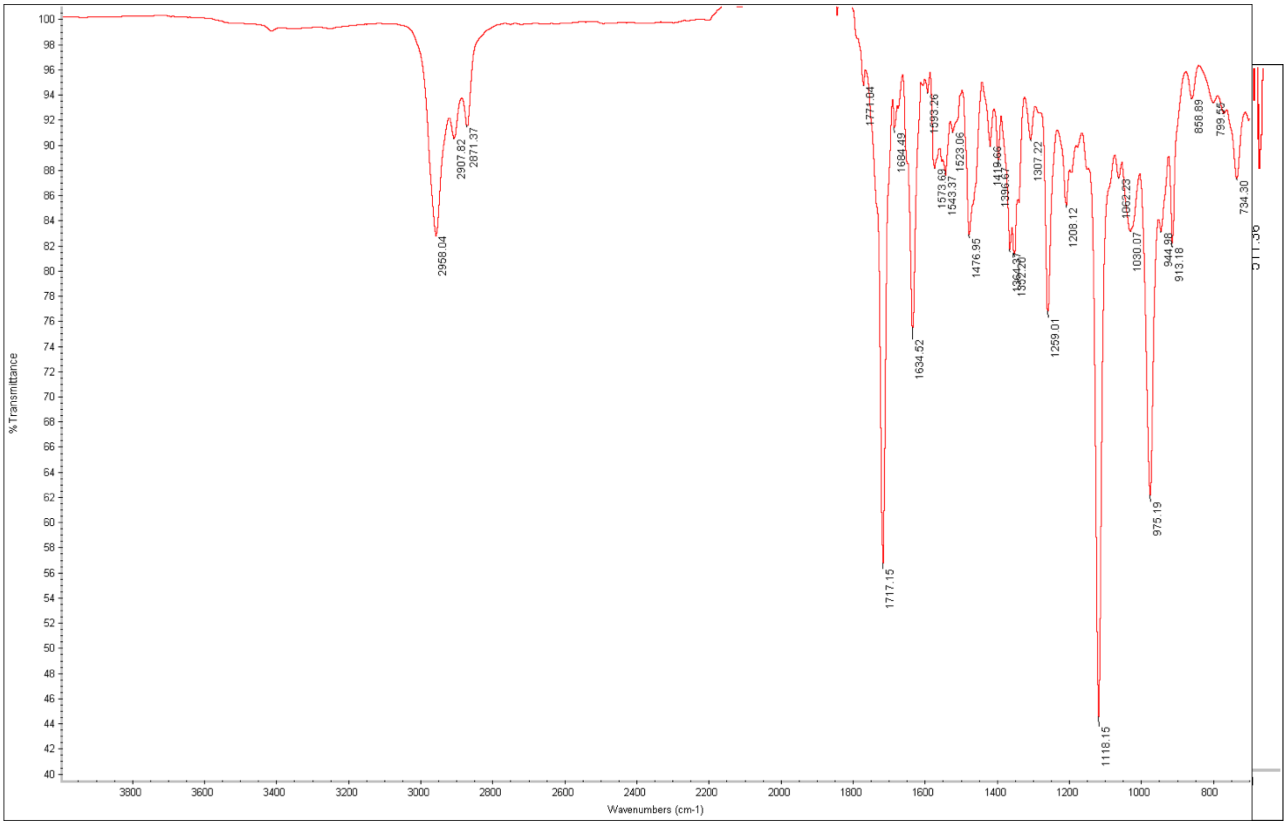

Figure S69. FT-IR spectrum of 1-((*S*)-4-isopropyl-4,5-dihydrooxazol-2-yl)ethan-1-ol, **2**.

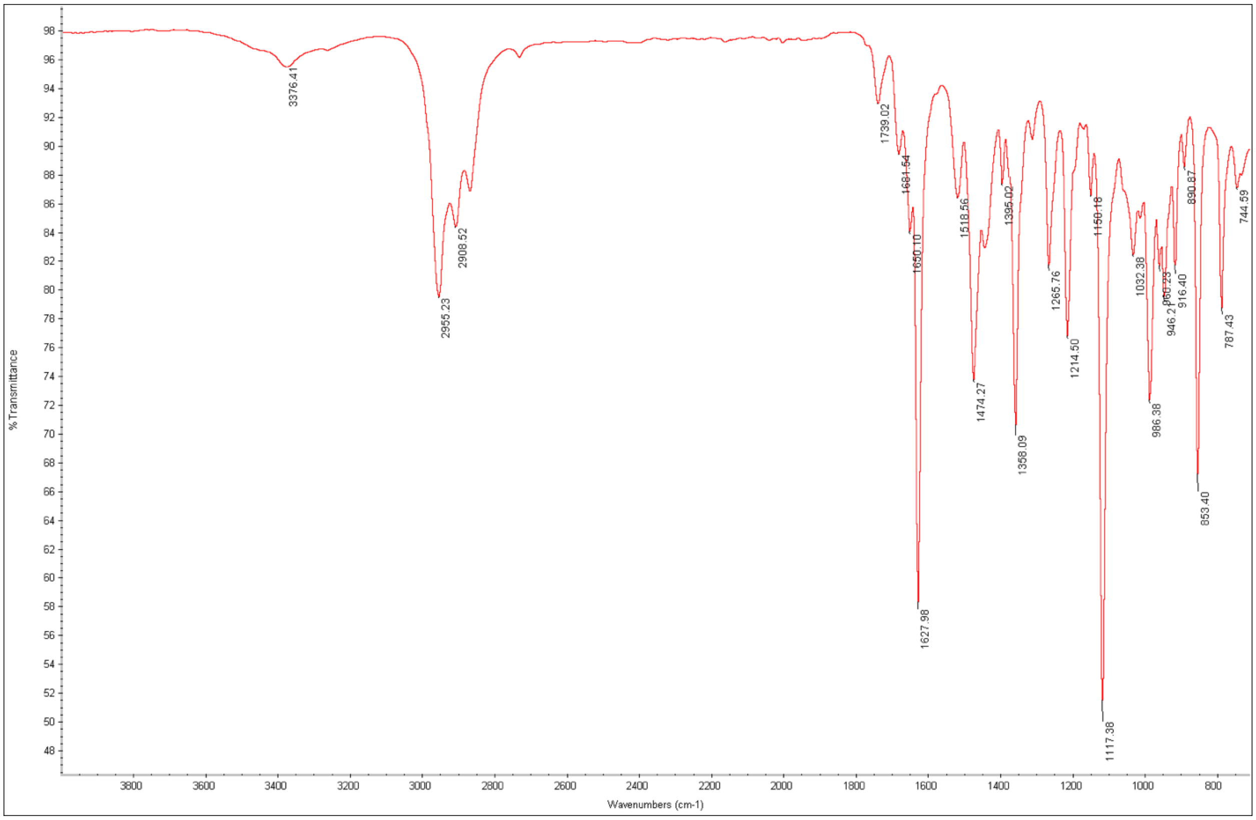

Figure S70. FT-IR spectrum of (*S*)-1-(4-(*tert*-butyl)-4,5-dihydrooxazol-2-yl)ethan-1-one, **3**.

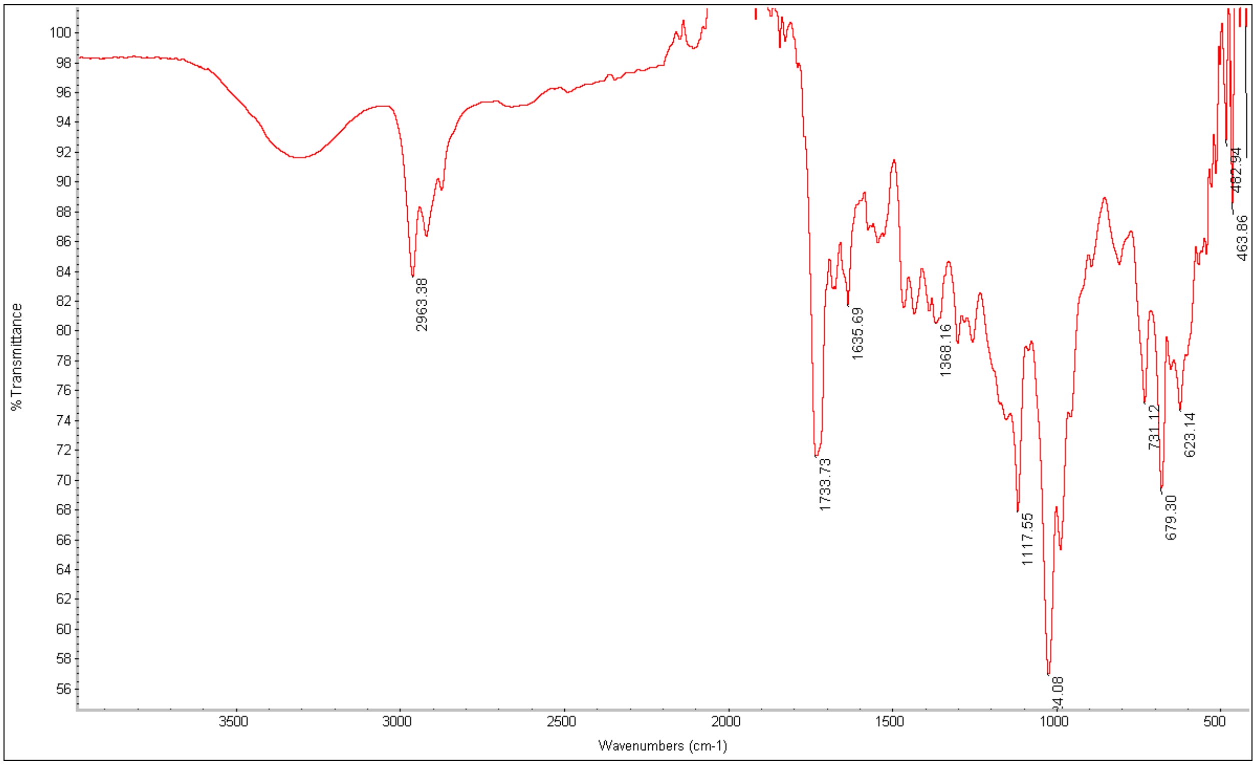

Figure S71. FT-IR spectrum of (*S*)-1-(4-isopropyl-4,5-dihydrooxazol-2-yl)ethan-1-one, **4**.

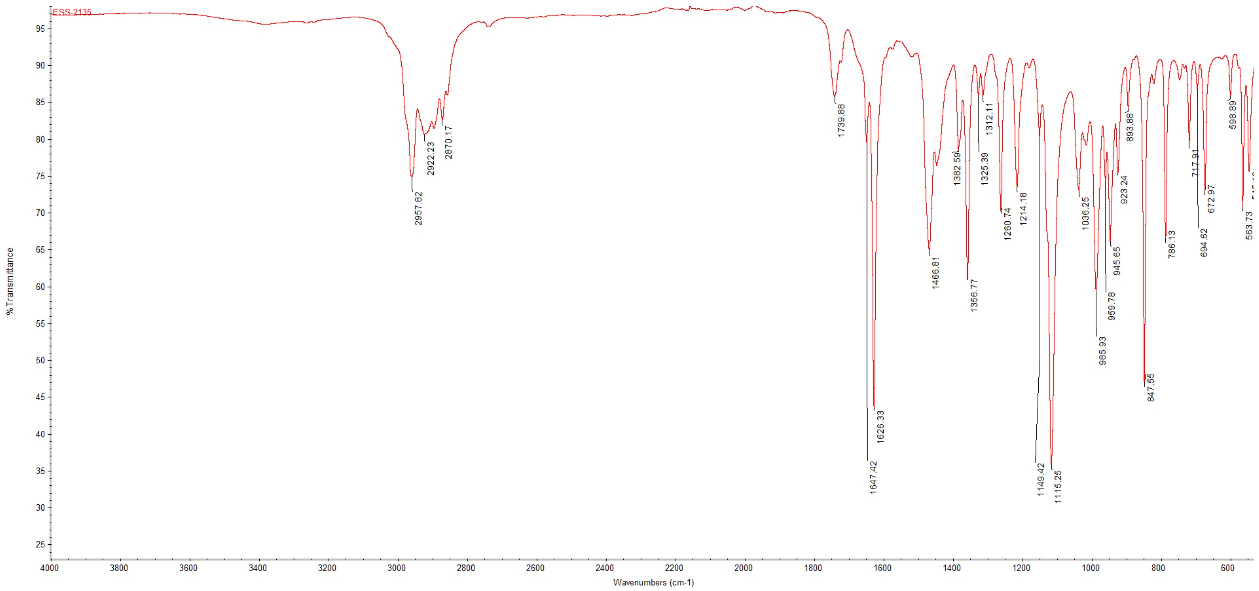

Figure S72. FT-IR spectrum of $^{Mes}Im^{iPr}Ox$, **6**.

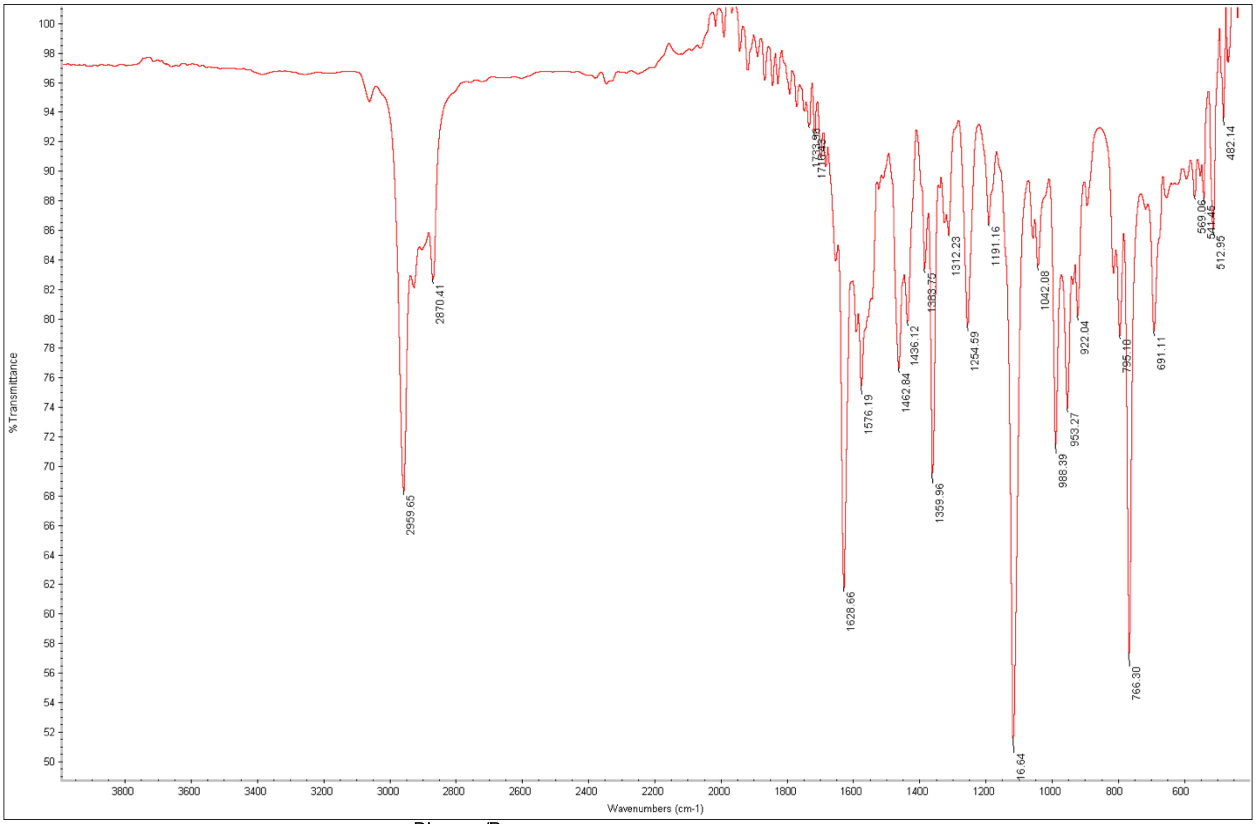

Figure S73. FT-IR spectrum of $^{Dipp}Im^{iPr}Ox$, **7**.

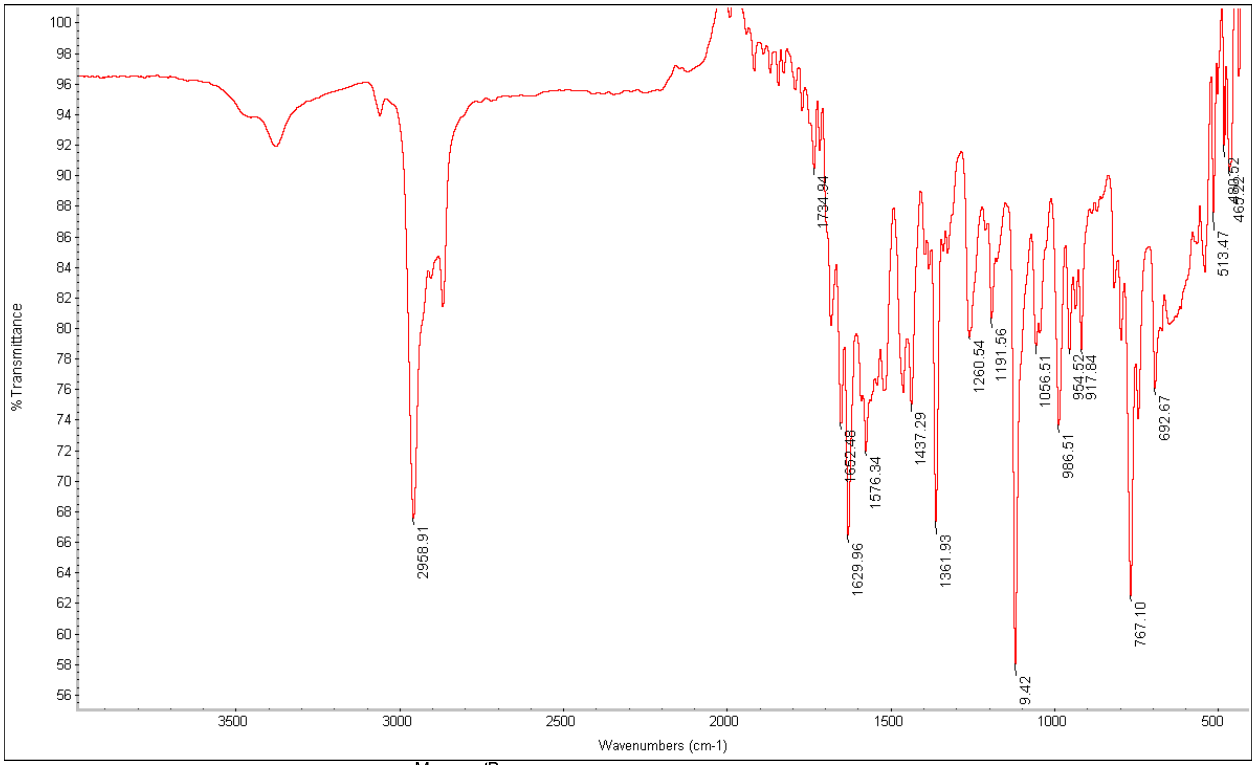

Figure S74. FT-IR spectrum of $^{Mes}Im^{tBu}Ox$, **8**.

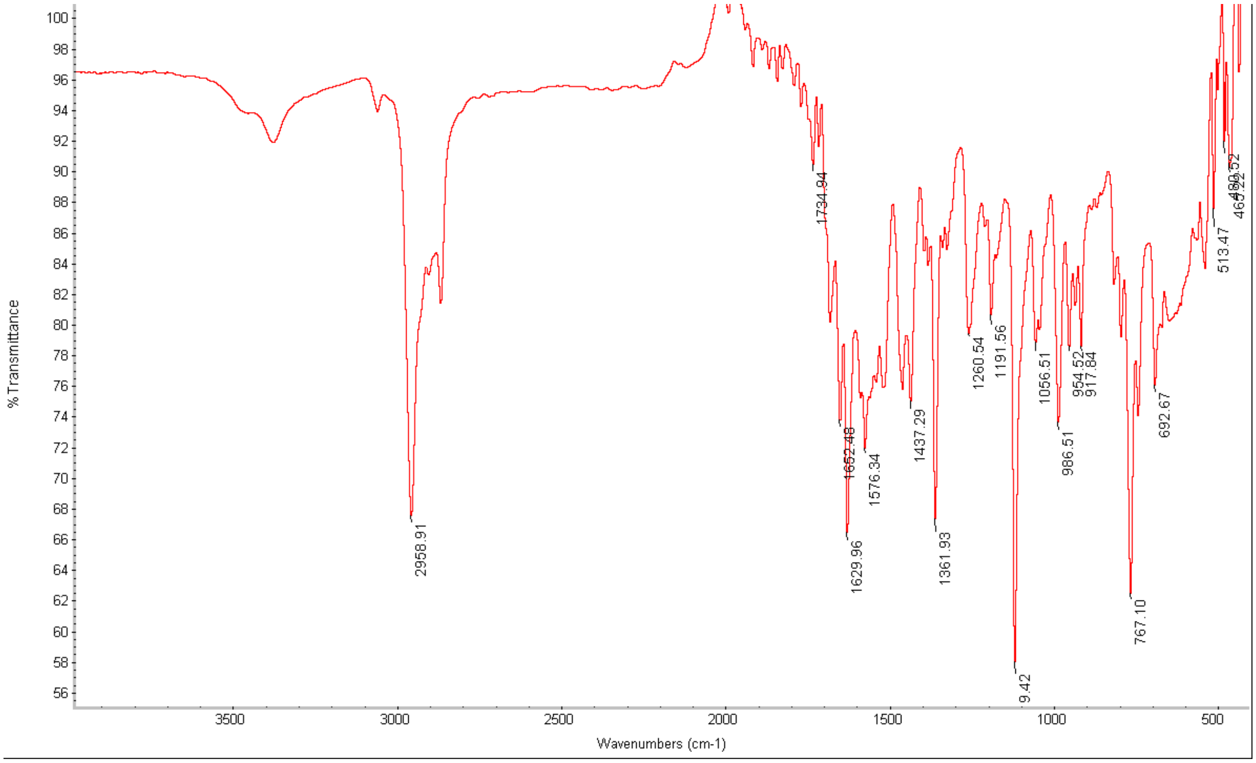

Figure S75. FT-IR spectrum of $^{Dipp}Im^{tBu}Ox$, **9**.

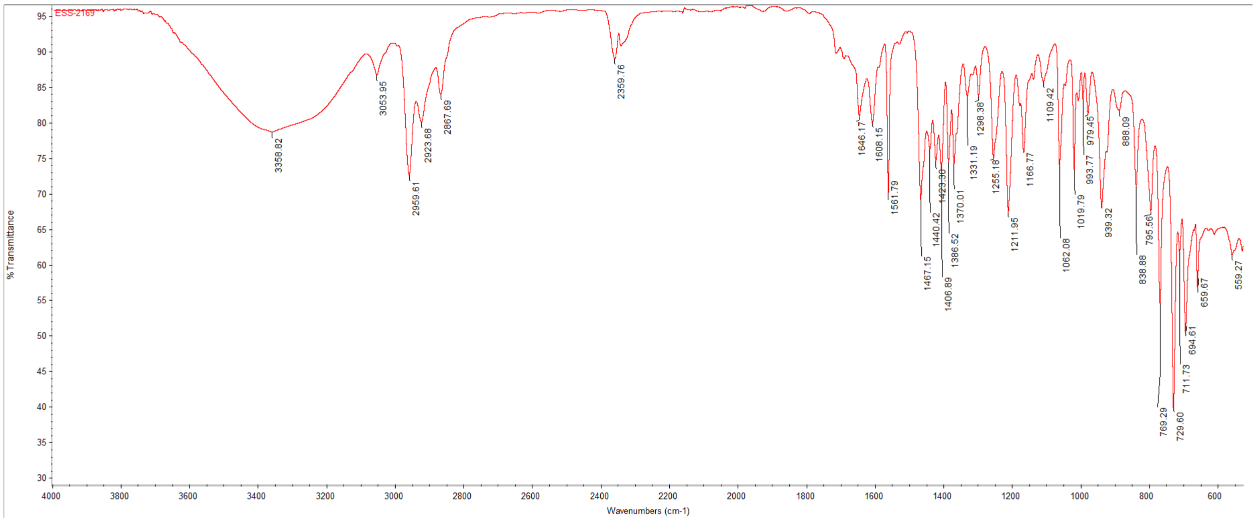

Figure S76. FT-IR spectrum of (^{Dipp}Im$^{i\text{Pr}}$Ox)Pd(Ph)I, **15**.

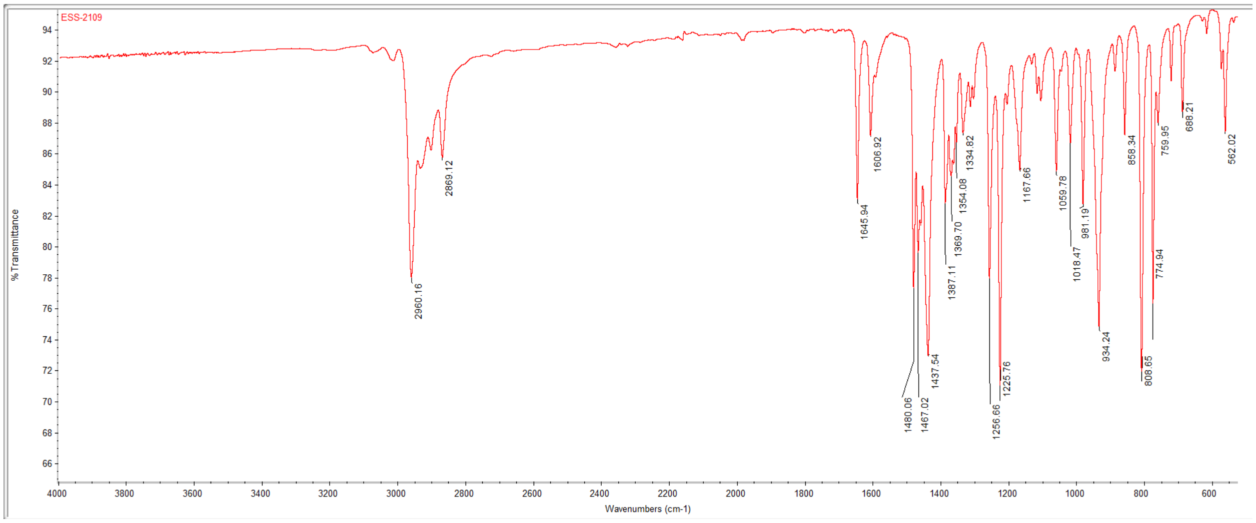

Figure S77. FT-IR spectrum of (^{Dipp}Im$^{i\text{Pr}}$Ox)$PdCl_2$ **19**.

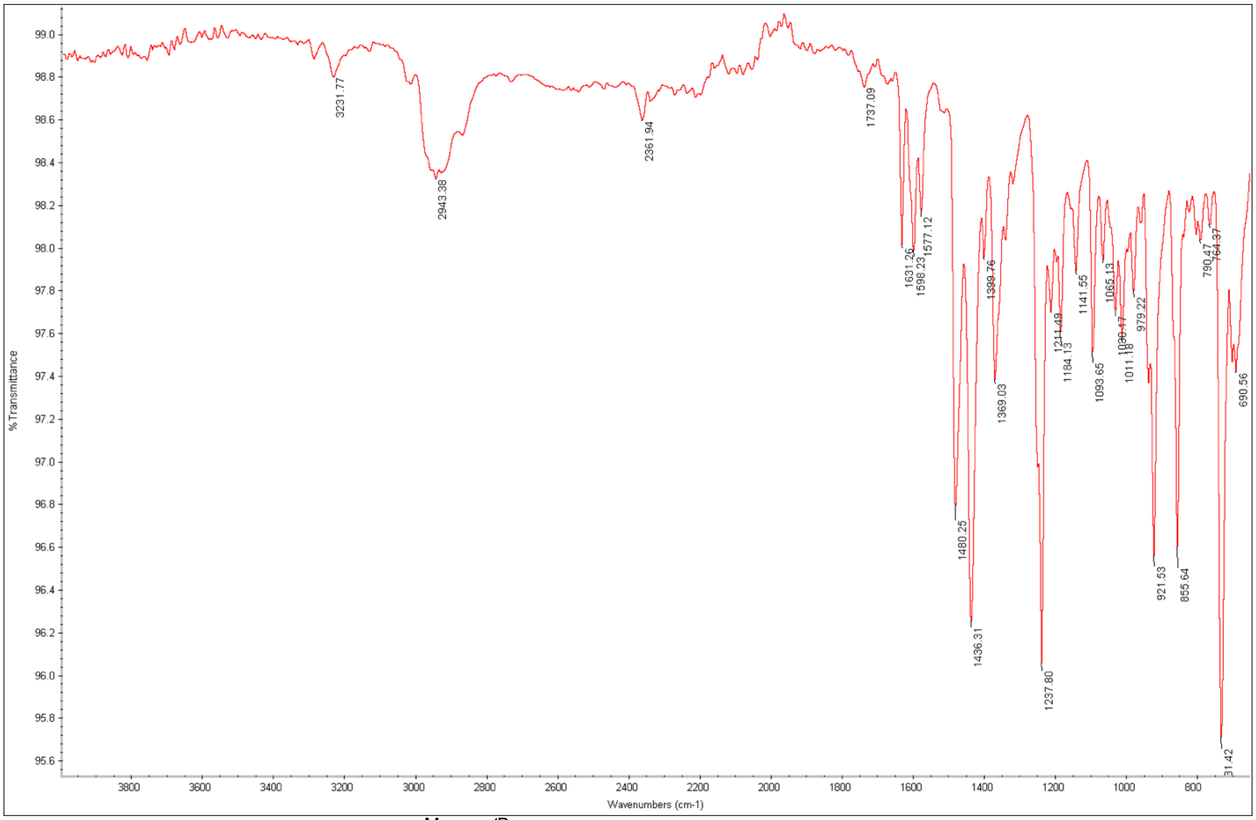

Figure S78. FT-IR spectrum of (^{Mes}Im^{tBu}Ox)PdCl$_2$, **20**.

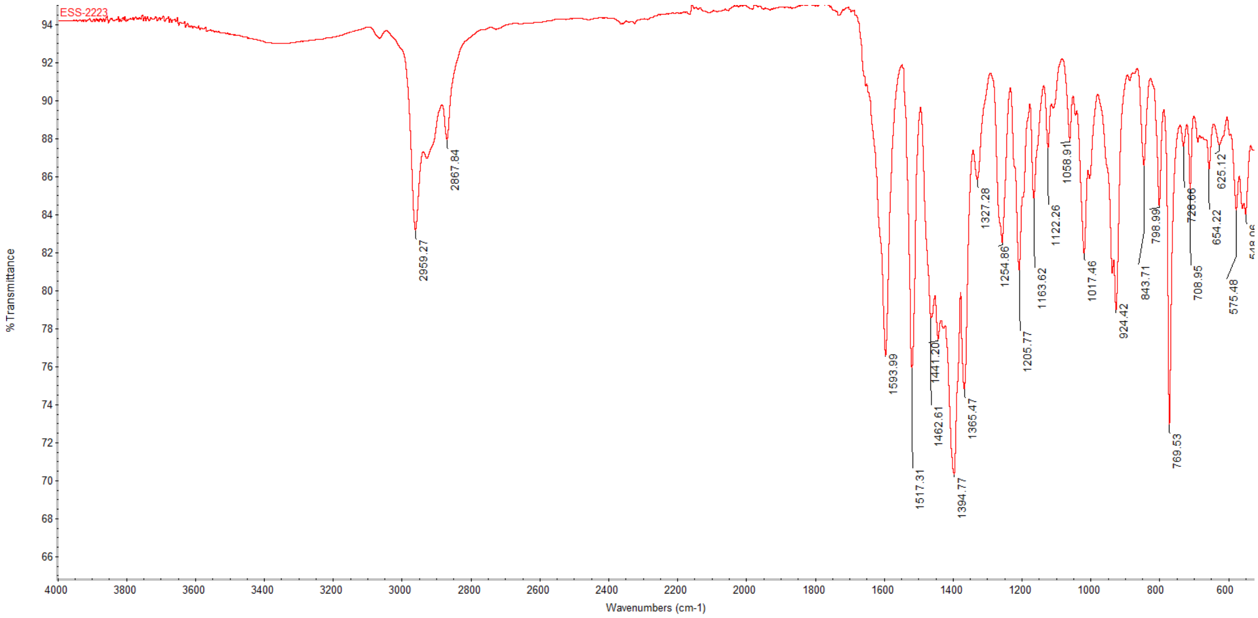

Figure S79. FT-IR spectrum of (^{Dipp}Im^{tBu}Ox)Ni(Mes)Br, **22**.

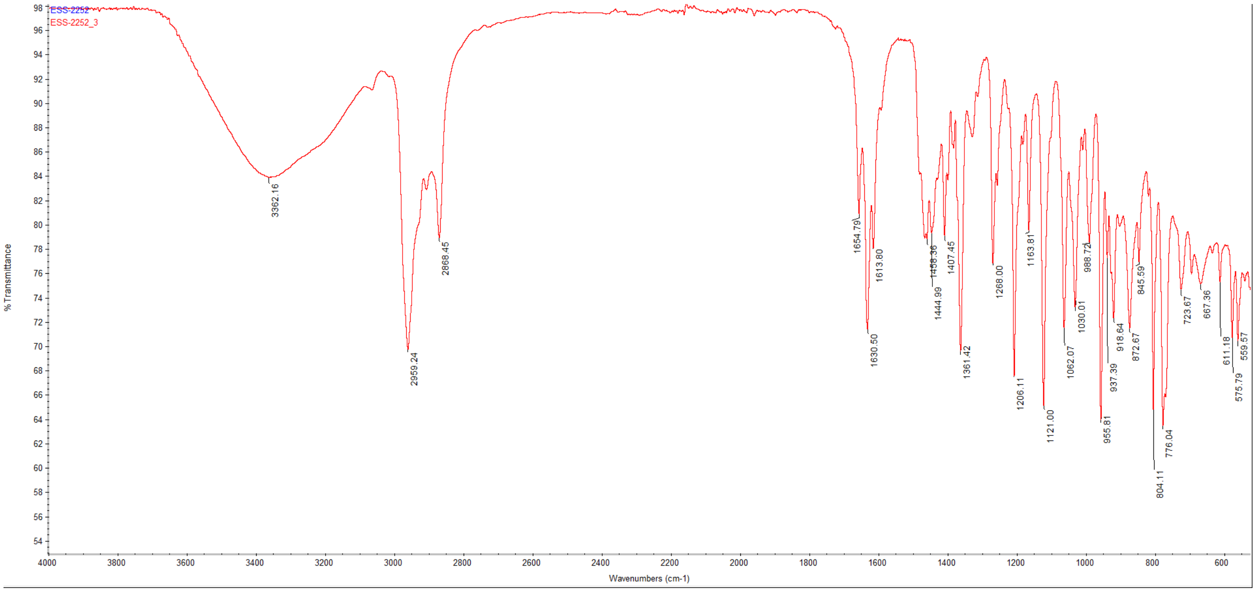

Figure 80. FT-IR spectrum of (^{Dipp}Im^{tBu}Ox)NiBr$_2$, **S4**.

15. Single-Crystal Structure Determination

Crystals were mounted on a MiTeGen MicroMount with Type B immersion oil (Cargille Labs). Single crystal X-ray diffraction data for **15**, **19**, **20** and **S4** were collected at 100 K on a Bruker SMART APEXII diffractometer equipped with a PHOTON-II-C14 detector. The X-ray beam generated from an INCOATEC micro-focused source was monochromated and collimated by a Montel multilayer optics (Mo $K\alpha$ radiation, λ = 0.71073 Å). X-ray diffraction data for **21**, **22** and **S1** were collected at 130 K on a Bruker D8 VENTURE diffractometer using Cu $K\alpha$ radiation, λ = 1.5406 Å (compounds **21** and **22**) or Mo $K\alpha$ radiation, λ = 0.71073 Å (compound **S1**). For both instruments, the data sets were processed with the INTEGRATE program of the APEX4 software for reduction and cell refinement.[29] Multi-scan absorption corrections were applied by the SCALE program for the area detector. The structures were solved by intrinsic phasing methods (SHELXT) and the structure models were completed and refined using the full-matrix least-square methods on F^2 (SHELXL). [30] All non-hydrogen atoms were refined with anisotropic displacement parameters, and hydrogen atoms on carbons were placed in idealized positions (C-H = 0.95-1.00 Å) and included as riding with U_{iso}(H) = 1.2 or 1.5 U_{eq}(non-H).

Selected crystallographic parameters are listed in Tables S7-S9. The molecular graphics (Figure S77-S83) in ORTEP style were drawn using Mercury (version 2023.3.1).[31] Crystallographic data of these structures, including cif, res, fcf, and hkl files, have been deposited with the Cambridge Crystallographic Data Centre with Numbers 2379525-2379527 and 2379601-2379604. Copies of these data can be requested, free of charge, from the CCDC website at https://www.ccdc.cam.ac.uk/structures/.

Table S7. Detailed crystallographic data.

**Compound name**	**($^{Dipp}Im^{iPr}Ox$)Pd(Ph)I**	**($^{Dipp}Im^{iPr}Ox$)PdCl$_2$**	**($^{Mes}Im^{tBu}Ox$)PdCl$_2$**
**Compound number**	**15**	**19**	**20**
**X-ray lab code**	24tnd10s	23tnd7s	23tnd10s
**CCDC deposition no.**	2379601	2379602	2379603
**Formula by X-ray**	$C_{30}H_{45}IN_2O_2Pd$	$C_{20}H_{30}Cl_2N_2OPd$	$C_{19}H_{28}Cl_4N_2OPd$
**Formula weight**	698.98	491.76	548.63
**Crystal habit**	Yellow plate	Orange needle	Orange needle
**Crystal size (mm^3)**	0.02 x 0.26 x 0.60	0.06 x 0.10 x 0.21	0.07 x 0.08 x 0.32
**Crystal system**	Monoclinic	Orthorhombic	Orthorhombic
**Space group**	$P2_1$	$P2_12_12_1$	$P2_12_12_1$
a **(Å)**	8.0979(6)	8.1986(4)	8.9867(3)
b **(Å)**	12.0067(8)	15.8752(8)	15.1263(5)
c **(Å)**	15.8790(11)	17.2380(9)	17.2304(5)
α **(°)**	90	90	90
β **(°)**	100.080(4)	90	90
γ **(°)**	90	90	90
V **(Å^3)**	1520.07(19)	2243.6(2)	2342.22(13)
Z, Z'	2, 1	4, 1	4, 1
D_c **(g cm^{-3})**	1.527	1.456	1.556
F**(000)**	1416	1008	1112
μ **(mm^{-1})**	1.654	1.076	1.260
**Total reflections**	24045	36987	29258
**Unique reflections**	7717	5708	5950
R_{int}	0.0507	0.0608	0.0781
$R_1{}^a$ **[$I > 2\sigma(I)$]**	0.0330	0.0221	0.0294
$wR_2{}^{b,c}$ **(all data)**	0.0687	0.0518	0.0679
$k_1, k_2{}^c$	0.0186, 0.6476	0.0286, 0.7941	0.0172, 0
**GOF (all data)**	1.064	1.063	1.031
**Flack parameter**	0.038(13)	0.027(13)	0.036(17)
**Restraints**	1	54	7

$^aR_1 = \Sigma||F_o|-|F_c||/\Sigma|F_o|$; $^bwR_2=\{\Sigma[w(F_o^2-F_c^2)^2]/\Sigma[w(F_o^2)^2]\}^{1/2}$; $^cw = 1/[(\sigma(F_o^2))^2+(k_1P)^2]+ k_2P]$, where $P = (F_o^2+2F_c^2)/3$.

Table S8. Detailed crystallographic data, continued.

**Compound name**	**(^{Mes}Im^{tBu}Ox)Ni(cod)**	**(^{Dipp}Im^{tBu}Ox)Ni(Mes)Br**	**(^{Mes}Im^{tBu}Ox)PdI$_2$**
**Compound number**	**21**	**22**	**S1**
**X-ray lab code**	ess1120ta4	ess2223	ess1173
**CCDC deposition no.**	2379525	2379527	2379526
**Formula by X-ray**	$C_{26}H_{38}N_2NiO$	$C_{30}H_{43}BrN_2NiO{\cdot}0.5(C_4H_{10}O)$	$C_{18}H_{26}I_2N_2OPd$
**Formula weight**	453.29	623.34	646.61
**Crystal habit**	Purple block	Orange needle	Red block
**Crystal size (mm^3)**	0.27 x 0.12 x 0.05	0.21 x 0.02 x 0.01	0.41 x 0.15 x 0.15
**Crystal system**	Monoclinic	Orthorhombic	Orthorhombic
**Space group**	$P2_1$	$P2_12_12_1$	$P2_12_12_1$
a **(Å)**	9.9627(4)	15.6080(5)	10.0504(8)
b **(Å)**	9.6608(4)	28.2109(10)	12.4967(10)
c **(Å)**	12.9145(6)	29.9287(10)	16.4837(12)
**α (°)**	90	90	90
**β (°)**	109.163(2)	90	90
**γ (°)**	90	90	90
V **(Å^3)**	1174.11(9)	13178.1(8)	2070.3(3)
Z, Z'	2, 1	16, 4	4
**D_c (g cm^{-3})**	1.282	1.257	2.075
F**(000)**	488	5264	1232
**μ (mm^{-1})**	1.324	2.461	3.886
**Total reflections**	27041	365522	138865
**Unique reflections**	4725	26946	13030
**R_{int}**	0.0547	0.1680	0.0433
**R_1^a [$I > 2\sigma(I)$]**	0.0438	0.0569	0.0132
**$wR_2^{b,c}$ (all data)**	0.1154	0.1344	0.0324
**k_1, k_2^c**	0.0774, 0.3294	0.0407, 33.3522	0.0134, 0.3089
**GOF (all data)**	1.133	1.047	1.088
**Flack parameter**	0.157(8)	-0.014(11)	-0.021(3)
**Restraints**	1	0	0

$^aR_1 = \Sigma||F_o|-|F_c||/\Sigma|F_o|$; $^bwR_2=\{\Sigma[w(F_o^2-F_c^2)^2]/\Sigma[w(F_o^2)^2]\}^{1/2}$; $^cw = 1/[(\sigma(F_o^2))^2+(k_1P)^2]+ k_2P]$, where $P = (F_o^2+2F_c^2)/3$.

Table S9. Detailed crystallographic data, continued

**Compound name**	**($^{Dipp}Im^{tBu}Ox$)$NiBr_2$**
**Compound number**	**S4**
**X-ray lab code**	ess2252
**CCDC deposition no.**	2379604
**Formula by X-ray**	$C_{29}H_{48}Br_2N_2O_3Ni$
**Formula weight**	691.22
**Crystal habit**	Orange block
**Crystal size (mm^3)**	0.46 x 0.11 x 0.09
**Crystal system**	Orthorhombic
**Space group**	$P2_12_12_1$
a **(Å)**	10.8161(4)
b **(Å)**	15.1190(5)
c **(Å)**	19.0767(7)
α **(°)**	90
β **(°)**	90
γ **(°)**	90
V **(Å^3)**	3119.59(19)
Z, Z'	4, 1
D_c **(g cm^{-3})**	1.472
***F*(000)**	1432
μ **(mm^{-1})**	3.215
**Total reflections**	40254
**Unique reflections**	7790
R_{int}	0.0560
$R_1{}^a$ **[$I > 2\sigma(I)$]**	0.0285
$wR_2{}^{b,c}$ **(all data)**	0.0645
k_1, $k_2{}^c$	0.0289, 0
**GOF (all data)**	1.008
**Flack parameter**	0.066(5)
**Restraints**	0

$^aR_1 = \Sigma||F_o|-|F_c||/\Sigma|F_o|$; $^bwR_2=\{\Sigma[w(F_o{}^2-F_c{}^2)^2]/\Sigma[w(F_o{}^2)^2]\}^{1/2}$; $^cw = 1/[(\sigma(F_o{}^2))^2+(k_1P)^2]+ k_2P]$, where $P = (F_o{}^2+2F_c{}^2)/3$.

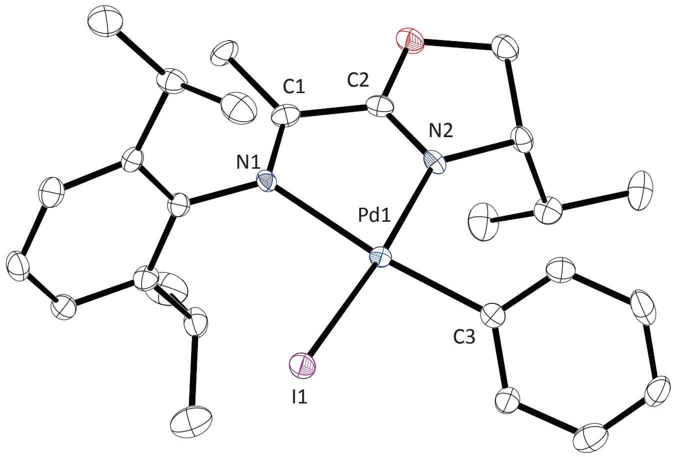

Figure S81. Molecular structure of ($^{Dipp}Im^{iPr}Ox$)Pd(Ph)I, **15**. The thermal ellipsoids are shown at 50% probability. Diethyl ether moiety as well as hydrogen atoms have been omitted for clarity. Selected bond angles (°) and distances (Å): Pd1–N1 2.202(4), Pd1–N2 2.050(4), N1–C1 1.280 (7), N2–C2 1.288(7), C1–C2 1.474(7), Pd1–I1 2.5664(5), Pd1–C3 1.979(5), C2–C1–N1 113.3(5), C1–C2–N2 121.1(5).

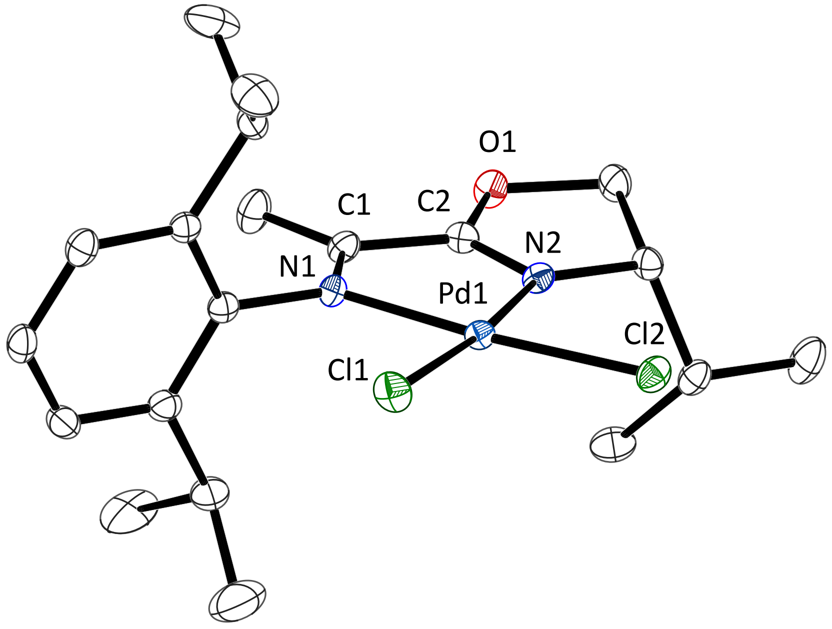

Figure S82. Molecular structure of ($^{Dipp}Im^{iPr}Ox$)$PdCl_2$, **19**. The thermal ellipsoids are shown at 50% probability. Hydrogen atoms have been omitted for clarity. Selected bond angles (°) and distances (Å): Pd1–N1 2.054(2), Pd1–N2 2.001(2), N1–C1 1.281(4), N2–C2 1.274(4), C1–C2 1.470(4), Pd1–Cl1 2.2818(7), Pd1–Cl2 2.2776(7), C2–C1–N1 112.3(2), C1–C2–N2 119.5(2).

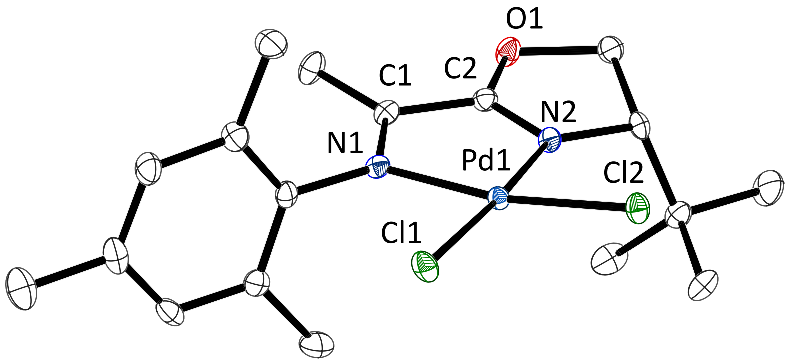

Figure S83. Molecular structure of (^{Mes}Im^{tBu}Ox)$PdCl_2$, **20**. The thermal ellipsoids are shown at 50% probability. Dichloromethane moiety as well as hydrogen atoms have been omitted for clarity. Selected bond angles (°) and distances (Å): Pd1–N1 2.047(3), Pd1–N2 2.023(3), N1–C1 1.285(5), N2–C2 1.282(5), C1–C2 1.479(5), Pd1–Cl1 2.2810(9), Pd1–Cl2 2.2809(10), C2–C1–N1 111.6(3), C1–C2–N2 120.3(3).

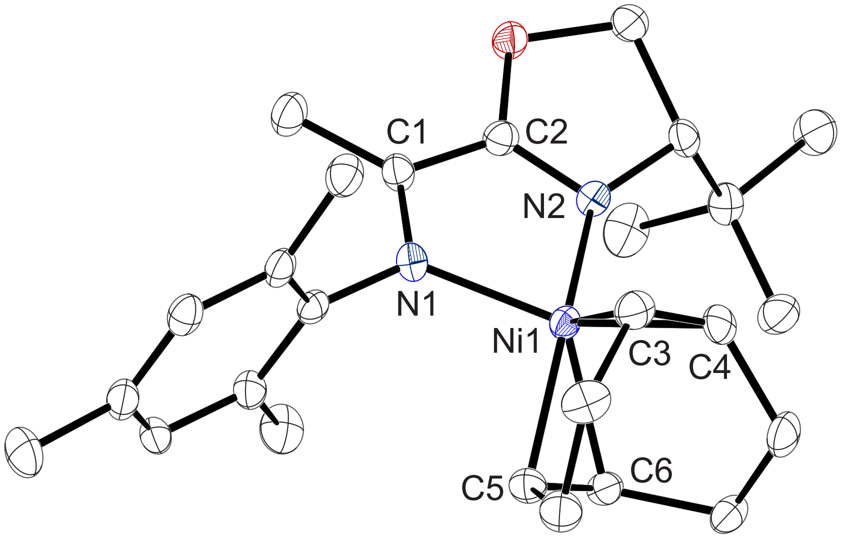

Figure S84. Molecular structure of (^{Mes}Im^{tBu}Ox)Ni(cod), **21**. The thermal ellipsoids are shown at 50% probability. Hydrogen atoms have been omitted for clarity. The crystal was treated as a monohedral twin, resulting in low precision for bond lenghts and angles. Selected bond angles (°) and distances (Å): Ni1–N1 1.950(2), Ni1–N2 1.990(3), N1–C1 1.338(4), N2–C2 1.312(4), C1–C2 1.410(4), Ni1–C3 2.069(3), Ni1–C4 2.069(3), Ni1–C5 2.054(3), Ni1–C6 2.087(3), C2–C1–N1 111.5(3), C1–C2–N2 120.6(3).

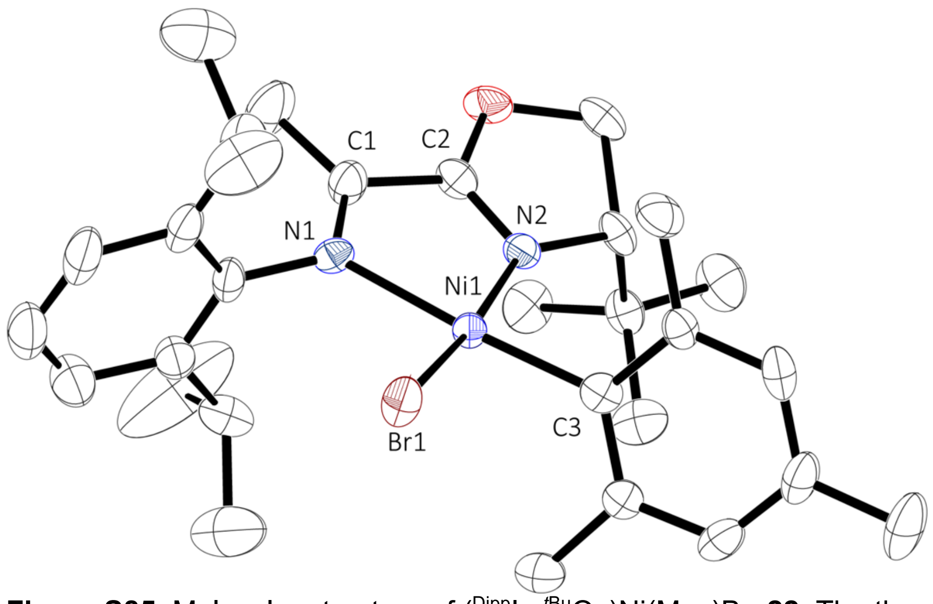

Figure S85. Molecular structure of (^{Dipp}Im^{tBu}Ox)Ni(Mes)Br, **22**. The thermal ellipsoids are shown at 50% probability. Hydrogen atoms and Et_2O have been omitted for clarity. Selected bond angles (°) and distances (Å): Ni1–N1 2.041(6), Ni1–N2 1.926(6), N1–N2 1.275(9), N2–C2 1.282(9), C1–C2 1.467(10), Ni1–Br1 2.2978(12), Ni1–C3 1.903(7), C2–C1–N1 111.4(6) C1–C2–N2 120.2(6).

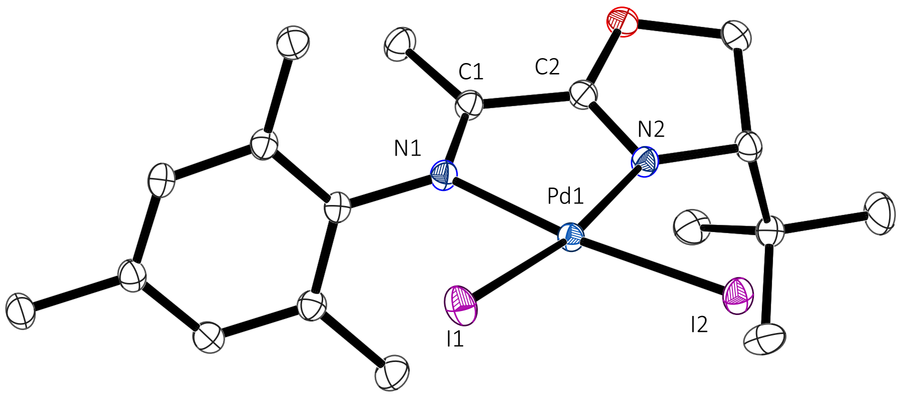

Figure S86. Molecular structure of (^{Mes}Im^{tBu}Ox)PdI_2, **S1**. The thermal ellipsoids are shown at 50% probability. Hydrogen atoms have been omitted for clarity. Selected bond angles (°) and distances (Å): Pd1–N1 2.1022(10), Pd1–N2: 2.0642(10), N1–C1 1.2900(16), N2–C2 1.2856(16), C1–C2 1.4779(17), Pd1–I1 2.5604(2), Pd1–I2 2.57134(18) C2–C1–N1 112.79(10) C1–C2–N2 120.53(11).

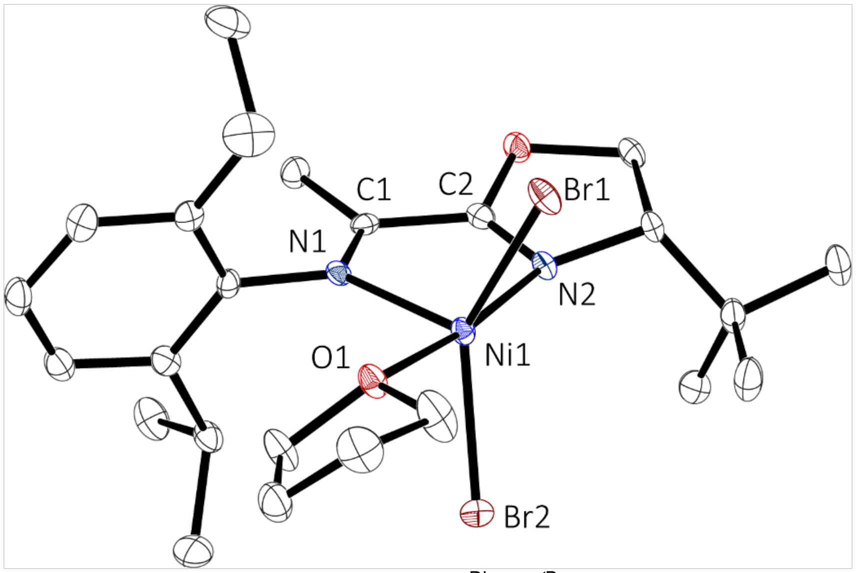

Figure S87. Molecular structure of (^{Dipp}Im^{tBu}Ox)NiBr$_2$•THF, **S4**. The thermal ellipsoids are shown at 50% probability. Hydrogen atoms have been omitted for clarity. Selected bond angles (°) and distances (Å): Ni1–N1 2.061(3), Ni1–N2 2.080(3), N1–C1 1.278(4), N2–C2 1.268(4), C1–C2 1.484(5), Ni1–Br1 2.4432(5), Ni1–Br2 2.4243(6), C2–C1–N1 113.2(3), C1–C2–N2 121.7(3).

16. DFT Calculations

The electronic structures of complexes (^{Mes}Im^{tBu}Ox)Ni(cod) **21** and [(^{Dipp}Im^{tBu}Ox)Ni(Mes)Br]$^{\bullet-}$ **23** were calculated using density functional theory (DFT) using the ORCA package. Geometry optimization of **21** using the B3LYP-D3[32]/m6-31g level of theory led to a structure inconsistent with the crystal data. In contrast, the B3LYP-D3/def2-TZVPP[33] level of theory gave results in much better agreement with the experimentally X-ray structures. In particular, both spin-unrestricted and broken-symmetry BS(1,1) calculations converged to the same geometry, with bond lengths closely matching those observed in crystal structure. A restricted singlet ground-state calculation gave a higher-energy solution with poorer agreement. Similarly, geometry optimizations of **23**, using the X-ray structure of **22** as the starting point and B3LYP-D3/def2-TVZPP, produced a structure with bond lengths more consistent with expected values.

Table S10. Comparison of calculated geometry and X-ray crystal structure of (^{Mes}Im^{tBu}Ox)Ni(cod), **21**

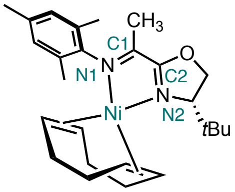

Method	N(1)–C(1) (Å)	N(2)–C(2) (Å)	C(1)–C(2) (Å)	Ni(1)–N(1) (Å)	Ni(1)–N(2) (Å)	Relative SPE (kcal/mol)
Crystal Structure	1.338(4)	1.312(4)	1.410(4)	1.950(2)	1.990(3)	-
**B3LYP-D3/def2-TZVPP (Unrestricted)**	**1.338**	**1.316**	**1.407**	**2.012**	**2.012**	**0.007**
B3LYP-D3/def2-TZVPP (Unrestricted BS(1,1) *S* = 0)	1.338	1.316	1.407	2.012	2.102	0
B3LYP-D3/def2-TZVPP (Restricted, *S* = 0)	1.320	1.304	1.419	1.970	2.001	+ 4.767
B3LYP/m6-31g* (Unrestricted)	1.389	1.367	1.463	2.025	2.190	-
B3LYP/ m6-31g* (Unrestricted BS(1,1) *S* = 0)	1.389	1.367	1.463	2.029	2.185	-

Table S11. Comparison of DFT computed geometry of [(^{Dipp}Im^{tBu}Ox)Ni(Mes)Br]$^{\bullet-}$, **23**

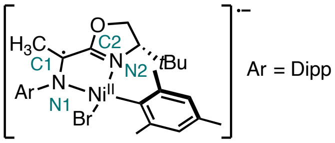

Method	N(1)–C(1) (Å)	N(2)–C(2) (Å)	C(1)–C(2) (Å)	Ni(1)–N(1) (Å)	Ni(1)–N(2) (Å)
Crystal Structure, **22**	1.275(9)	1.282(9)	1.467(10)	2.041(6)	1.926(6)
**B3LYP-D3/def2-TZVPP (**Unrestricted**)**	**1.346**	**1.325**	**1.395**	**2.018**	**1.928**
B3LYP/m6-31g* (Unrestricted)	1.389	1.367	1.463	2.025	2.190

Input File for Calculation of (^{Mes}Im^{tBu}Ox)Ni(cod), 21

```
#ImOxNiCod_Brokensym-run2-B3LYP-D3_TZVPP
%maxcore 3200
%PAL NPROCS 28 END
! UKS B3LYP D3 RIJCOSX SlowConv TightSCF def2-SVP def2/j Normalprint UCO OPT FREQ

%basis NewGTO 28 "def2-TZVPP" end
NewGTO 8 "def2-TZVPP" end
NewGTO 7 "def2-TZVPP" end
NewGTO 6 "def2-TZVPP" end
NewAuxGTO 28 "def2/j" end
NewAuxGTO 8 "def2/j" end
NewAuxGTO 7 "def2/j" end
NewAuxGTO 6 "def2/j" end
end

%scf brokensym 1,1
MaxIter 1500
TolE 1E-7
TolErr 1E-6
end

* xyz 0 1
Ni   3.45441   4.69210   8.63294
O    1.39686   1.39884   9.75752
N    3.11507   2.83825   9.26836
N    1.53805   4.86020   8.98414
C    3.74498   1.50028   9.41962
H    4.28428   1.30010   8.60103
C    2.52860   0.55355   9.46231
H    2.40821   0.09878   8.59146
H    2.64497  -0.13284  10.16685
C    1.82730   2.66533   9.49647
C    0.93285   3.75407   9.41840
C   -0.50427   3.65167   9.79533
H   -1.06380   3.82175   9.00665
H   -0.71161   4.31249  10.48862
H   -0.69228   2.75141  10.13688
C    4.66834   1.41719  10.66752
C    5.21532  -0.01932  10.78218
H    5.97967  -0.02811  11.39569
H    5.50310  -0.32940   9.89797
H    4.51348  -0.60913  11.12412
C    3.89151   1.79685  11.93981
H    4.49358   1.74933  12.71372
H    3.14767   1.17451  12.06627
H    3.54713   2.70919  11.85117
C    5.82672   2.38035  10.49064
H    6.45775   2.27321  11.23248
H    5.48842   3.30017  10.47960
H    6.28339   2.19088   9.64380
```

```
C     0.74425    6.04071    8.91461
C     0.06471    6.34212    7.72770
C    -0.67430    7.51587    7.65817
H    -1.13958    7.71942    6.85480
C    -0.75425    8.40077    8.72553
C    -0.09279    8.06941    9.91000
H    -0.14987    8.65550   10.65515
C     0.65215    6.89277   10.01978
C     0.11463    5.40215    6.55177
H    -0.25123    5.84943    5.76057
H    -0.41525    4.60227    6.74989
H     1.04460    5.14390    6.38034
C    -1.50950    9.69914    8.59379
H    -2.35941    9.53952    8.13335
H    -0.97402   10.33866    8.07727
H    -1.68507   10.06774    9.48513
C     1.32757    6.54982   11.31891
H     1.33200    7.33090   11.90618
H     2.25403    6.26959   11.14161
H     0.84803    5.81305   11.75463
C     4.95949    4.30665    7.26904
H     5.03595    3.34376    7.01036
C     3.92671    4.97226    6.64570
H     3.39693    4.40782    6.01236
C     3.90114    6.45321    6.33220
H     2.98079    6.71621    6.07992
H     4.49175    6.63233    5.55783
C     4.36468    7.31300    7.53862
H     5.34768    7.43462    7.49636
H     3.94457    8.20698    7.48654
C     3.99816    6.66091    8.85727
H     3.37458    7.19962    9.42344
C     4.89440    5.87165    9.57820
H     4.80921    5.93155   10.57254
C     6.29204    5.57411    9.06831
H     6.73745    4.94742    9.69406
H     6.81703    6.41094    9.05206
C     6.28740    4.94618    7.65085
H     6.50072    5.65723    6.99159
H     7.00024    4.26583    7.59999
*

%plots format cube
dim1 100 dim2 100 dim3 100
SpinDens("ImOxNiCod.cube");
End
```

Input File for Calculation of [(^{Dipp}Im^{tBu}Ox)Ni(Mes)Br]$^{\bullet-}$, 23

```
#ImOxNiMesBr
%maxcore 3200
%PAL NPROCS 28 END
```

```
! UKS B3LYP D3 RIJCOSX SlowConv TightSCF def2-SVP def2/j Normalprint UCO OPT FREQ

%basis NewGTO 28 "def2-TZVPP" end
NewGTO 35 "def2-TZVPP" end
NewGTO 8 "def2-TZVPP" end
NewGTO 7 "def2-TZVPP" end
NewGTO 6 "def2-TZVPP" end
NewAuxGTO 28 "def2/j" end
NewAuxGTO 35 "def2/j" end
NewAuxGTO 8 "def2/j" end
NewAuxGTO 7 "def2/j" end
NewAuxGTO 6 "def2/j" end
end

%scf
MaxIter 1500
TolE 1E-7
TolErr 1E-6
end

* xyz 1 2
Ni    13.957061   11.882579   24.548512
  Br    12.163668   11.499512   23.040330
  O     16.146294   11.116372   27.825186
  N     15.402419   12.002878   25.867598
  N     12.976799   11.111717   26.183391
  C     13.388702   10.340059   28.560847
  H     12.305858   10.224813   28.609325
  H     13.709603   10.987036   29.381972
  H     13.839298    9.356909   28.733961
  C     13.791924   10.916211   27.236902
  C     15.091602   11.352056   26.982387
  C     17.313418   11.412976   27.024572
  H     17.654057   10.485838   26.550467
  H     18.086729   11.788711   27.691549
  C     16.804471   12.412859   25.979627
  H     17.305589   12.278122   25.018143
  C     17.012155   13.894302   26.423954
  C     16.337678   14.823821   25.418636
  H     16.527547   15.868705   25.685040
  H     15.262549   14.657114   25.412117
  H     16.698892   14.649751   24.403576
  C     18.517716   14.188446   26.457151
  H     18.692366   15.239820   26.705214
  H     18.979118   13.991465   25.484216
  H     19.040263   13.590413   27.206445
  C     16.422345   14.155203   27.815353
  H     16.576750   15.203865   28.087490
  H     16.888800   13.540014   28.586203
  H     15.348941   13.953018   27.824394
  C     14.999754   12.566312   23.097047
```

C	14.811551	13.867669	22.608358
C	15.616532	14.363148	21.583271
H	15.446582	15.375711	21.222942
C	16.627704	13.599577	21.011734
C	16.793990	12.298539	21.476175
H	17.557686	11.669713	21.024332
C	15.993165	11.776036	22.495275
C	13.711883	14.751088	23.137885
H	14.001293	15.806095	23.096138
H	13.450784	14.490305	24.163752
H	12.801083	14.615411	22.547319
C	17.518355	14.170985	19.942021
H	16.945012	14.763390	19.221601
H	18.039934	13.382250	19.393180
H	18.281508	14.831974	20.371206
C	16.175600	10.334988	22.899597
H	16.982924	9.862228	22.334518
H	15.253449	9.773801	22.721502
H	16.395124	10.239394	23.968200
C	11.630772	10.714326	26.216315
C	10.633475	11.701408	26.302288
C	9.297252	11.317775	26.261097
H	8.522576	12.074630	26.315422
C	8.940383	9.982158	26.137444
H	7.894973	9.695473	26.100920
C	9.930709	9.015398	26.043485
H	9.647995	7.975321	25.920038
C	11.279976	9.360721	26.068618
C	11.027014	13.155055	26.458515
H	12.100210	13.206775	26.270606
C	10.790529	13.620089	27.900407
H	11.163323	14.639217	28.047452
H	11.312794	12.965855	28.602398
H	9.725523	13.603585	28.152290
C	10.353547	14.066563	25.434805
H	10.740506	15.086314	25.527667
H	9.268399	14.106004	25.573986
H	10.565184	13.704588	24.426868
C	12.343826	8.301696	25.845599
H	13.310716	8.790737	25.954196
C	12.277611	7.174929	26.879685
H	12.371710	7.572920	27.892825
H	13.086291	6.455486	26.718764
H	11.331260	6.629050	26.818761
C	12.270429	7.766430	24.411014
H	12.349270	8.590095	23.698877
H	11.323502	7.247868	24.231254
H	13.081711	7.055648	24.222745

*

%plots format cube

```
dim1 100 dim2 100 dim3 100
SpinDens("ImOxNiMesBr.cube");
End
```

17. References

[1] Hossain, A. M. S.; Balbín, A.; Erami, R. S.; Prashar, S.; Fajardo, M.; Gómez-Ruiz, S. Synthesis and study of the catalytic applications in C–C coupling reactions of hybrid nanosystems based on alumina and palladium nanoparticles. *Inorg. Chim. Acta* **2017**, *455*, 645-652.

[2] Martin, N. J. A.; List, B. Highly Enantioselective Transfer Hydrogenation of α,β-Unsaturated Ketones. *J. Am. Chem. Soc.* **2006**, *128* (41), 13368-13369.

[3] Ward, L. G. L.; Pipal, J. R. Anhydrous Nickel(II) Halides and their Tetrakis(ethanol) and 1,2-Dimethoxyethane Complexes. In *Inorg. Synth.* **1972**, *8,* 154-164.

[4] (a) Neese, F.; Wennmohs, F.; Becker, U.; Riplinger, C. The ORCA quantum chemistry program package. *The J. Chem. Phys.* **2020**, *152* (22). (b) Neese, F. Software update: The ORCA program system—Version 5.0. *Wiley Interdiscip. Rev.: Comput. Mol. Sci.* **2022**, *12* (5), e1606

[5] De Mello, M. B. M.; Clososki, G. C.; Piovan, L.; De Oliveira, A. R. M. An alternative approach to differentially substituted 2-oxazoline chalcogen derivatives. *J. Organomet. Chem.* **2015**, *794*, 11-16.

[6] Seayad, J.; Patra, P. K.; Zhang, Y.; Ying, J. Y. Organocatalytic Synthesis of N-Phenylisoxazolidin-5-ones and a One-Pot Synthesis of β-Amino Acid Esters. *Org. Lett.* **2008**, *10* (5), 953-956.

[7] Kikushima, K.; Holder, J. C.; Gatti, M.; Stoltz, B. M. Palladium-Catalyzed Asymmetric Conjugate Addition of Arylboronic Acids to Five-, Six-, and Seven-Membered β-Substituted Cyclic Enones: Enantioselective Construction of All-Carbon Quaternary Stereocenters. *J. Am. Chem. Soc.* **2011**, *133* (18), 6902-6905.

[8] Shockley, S. E.; Holder, J. C.; Stoltz, B. M. Palladium-Catalyzed Asymmetric Conjugate Addition of Arylboronic Acids to α,β-Unsaturated Cyclic Electrophiles. *OPR&D.* **2015**, *19* (8), 974-981.

[9] Van Zeeland, R.; Stanley, L. M. Palladium-Catalyzed Conjugate Addition of Arylboronic Acids to β,β-Disubstituted Enones in Aqueous Media: Formation of Bis-benzylic and *ortho*-Substituted Benzylic Quaternary Centers. *ACS Catal.* **2015**, *5* (9), 5203-5206.

[10] Kadam, A. A.; Ellern, A.; Stanley, L. M. Enantioselective, Palladium-Catalyzed Conjugate Additions of Arylboronic Acids to Form Bis-benzylic Quaternary Stereocenters. *Org. Lett*. **2017**, *19* (15), 4062-4065

[11] Shintani, R.; Tsutsumi, Y.; Nagaosa, M.; Nishimura, T.; Hayashi, T. Sodium Tetraarylborates as Effective Nucleophiles in Rhodium/Diene-Catalyzed 1,4-Addition to β,β-Disubstituted α,β-Unsaturated Ketones: Catalytic Asymmetric Construction of Quaternary Carbon Stereocenters. *J. Am. Chem. Soc.* **2009**, *131* (38), 13588-13589.

[12] Shintani, R.; Takeda, M.; Nishimura, T.; Hayashi, T. Chiral Tetrafluorobenzobarrelenes as Effective Ligands for Rhodium-Catalyzed Asymmetric 1,4-Addition of Arylboroxines to β,β-Disubstituted α,β-Unsaturated Ketones. *Angew. Chem. Int. Ed.* **2010**, *49* (23), 3969-3971.

[13] Holder, J. C.; Zou, L.; Marziale, A. N.; Liu, P.; Lan, Y.; Gatti, M.; Kikushima, K.; Houk, K. N.; Stoltz, B. M. Mechanism and Enantioselectivity in Palladium-Catalyzed Conjugate Addition of Arylboronic Acids to β-Substituted Cyclic Enones: Insights from Computation and Experiment. *J. Am. Chem. Soc.* **2013**, *135* (40), 14996-15007.

[14] Pichon-Barré, D.; Zhang, Z.; Cador, A.; Vives, T.; Roisnel, T.; Baslé, O.; Jarrige, L.; Cavallo, L.; Falivene, L.; Mauduit, M. Chiral oxazolidines acting as transient hydroxyalkyl-

functionalized N-heterocyclic carbenes: an efficient route to air stable copper and gold complexes for asymmetric catalysis. *Chem. Sci.* **2022**, *13* (30), 8773-8780.
15 Behenna, D. C.; Liu, Y.; Yurino, T.; Kim, J.; White, D. E.; Virgil, S. C.; Stoltz, B. M. Enantioselective construction of quaternary N-heterocycles by palladium-catalysed decarboxylative allylic alkylation of lactams. *Nat. Chem.* **2012**, *4* (2), 130-133.
16 Gottumukkala, A. L.; Matcha, K.; Lutz, M.; De Vries, J. G.; Minnaard, A. J. Palladium-Catalyzed Asymmetric Quaternary Stereocenter Formation. *Chem. Eur. J.* **2012**, *18* (22), 6907-6914.
17 Carrow, B. P.; Hartwig, J. F. Distinguishing Between Pathways for Transmetalation in Suzuki−Miyaura Reactions. *J. Am. Chem. Soc.* **2011**, *133* (7), 2116-2119.
18 Hayes, H. L. D.; Wei, R.; Assante, M.; Geogheghan, K. J.; Jin, N.; Tomasi, S.; Noonan, G.; Leach, A. G.; Lloyd-Jones, G. C. Protodeboronation of (Hetero)Arylboronic Esters: Direct versus Prehydrolytic Pathways and Self-/Auto-Catalysis. *J. Am. Chem. Soc.* **2021**, *143* (36), 14814-14826.
19 Yang, J.; Neary, M. C.; Diao, T. ProPhos: A Ligand for Promoting Nickel-Catalyzed Suzuki-Miyaura Coupling Inspired by Mechanistic Insights into Transmetalation. *J. Am. Chem. Soc.* **2024**, *146* (9), 6360-6368.
20 Gottumukkala, A. L.; Suljagic, J.; Matcha, K.; de Vries, J. G.; Minnaard, A. J. Efficient Formation of Benzylic Quaternary Centers via Palladium Catalysis. *ChemSusChem* **2013**, *6* (9), 1636-1639.
21 Pucheault, M.; Darses, S.; Genet, J.-P. Potassium organotrifluoroborates: new partners in catalytic enantioselective conjugate additions to enones. *Tetrahedron Lett.* **2002**, *43* (35), 6155-6157.
22 Kurihara, K.; Sugishita, N.; Oshita, K.; Piao, D.; Yamamoto, Y.; Miyaura, N. Enantioselective 1,4-addition of arylboronic acids to α,β-unsaturated carbonyl compounds catalyzed by rhodium(I)-chiral phosphoramidite complexes. *J. Organomet. Chem.* **2007**, *692* (1-3), 428-435.
23 Shimada, T.; Suda, M.; Nagano, T.; Kakiuchi, K. Facile Preparation of a New BINAP-Based Building Block, 5,5‘-DiiodoBINAP, and Its Synthetic Application. *J. Org. Chem.* **2005**, *70* (24), 10178-10181.
24 Kuuloja, N.; Tois, J.; Franzén, R. Indole-olefin-oxazoline (IndOlefOx)-ligands: synthesis and utilization in asymmetric Rh-catalyzed conjugate addition. *Tetrahedron: Asymmetry* **2011**, *22* (4), 468-475.
25 Martin, N. J. A.; List, B. Highly Enantioselective Transfer Hydrogenation of α,β-Unsaturated Ketones. *J. Am. Chem. Soc.* **2006**, *128* (41), 13368-13369.
26 Verendel, J. J.; Li, J. Q.; Quan, X.; Peters, B.; Zhou, T.; Gautun, O. R.; Govender, T.; Andersson, P. G. Chiral Hetero- and Carbocyclic Compounds from the Asymmetric Hydrogenation of Cyclic Alkenes. *Chem. Eur. J.* **2012**, *18* (21), 6507-6513.
27 Kim, J.; Hong, S. H. Ligand-Promoted Direct C–H Arylation of Simple Arenes: Evidence for a Cooperative Bimetallic Mechanism. *ACS Catal.* **2017**, *7* (5), 3336-3343.
28 Dawson, G. A.; Lin, Q.; Neary, M. C.; Diao, T. Ligand Redox Activity of Organonickel Radical Complexes Governed by the Geometry.*J. Am. Chem. Soc.* **2023**, *145* (37), 20551-20561.
29 APEX 4. Bruker APX, Madison, WI, 2020.
30 Sheldrick, G. M. *SHELXT*– Integrated space-group and crystal-structure determination. *Acta Cryst.* **2015**, *71* (1), 3-8.
31 Macrae, C. F.; Sovago, I.; Cottrell, S. J.; Galek, P. T. A.; McCabe, P.; Pidcock, E.; Platings, M.; Shields, G. P.; Stevens, J. S.; Towler, M.; Wood, P. A. *Mercury 4.0*: from visualization to analysis, design and prediction. *J. App. Crystallogr.* **2020**, *53* (1), 226-235.
32 (a) Becke, A. D. Density-functional thermochemistry. III. The role of exact exchange. *J. Chem. Phys*. **1993**, *98* (7), 5648–5652. (b) Lee, C.; Yang, W.; Parr, R. G. Development of

the Colle-Salvetti correlation-energy formula into a functional of the electron density. *Phys. Rev. B* **1988**, *37* (2), 785-789. (c) Grimme, S. Semiempirical GGA-type density functional constructed with a long-range dispersion correction. *J. Comput. Chem.* **2006**, *27* (15), 1787–1799

33 (a) Grimme, S.; Ehrlich, S.; Goerigk, L. Effect of the damping function in dispersion corrected density functional theory. *J. Comput. Chem.* **2011**, *32* (7), 1456–1465. (b) Grimme, S.; Antony, J.; Ehrlich, S.; Krieg, H. A consistent and accurate *ab initio* parametrization of density functional dispersion correction (DFT-D) for the 94 elements H-Pu.*J. Chem. Phys.* **2010**, *132* (15), 154104. (c) Weigend, F.; Ahlrichs, R. Balanced basis sets of split valence, triple zeta valence and quadruple zeta valence quality for H to Rn: Design and assessment of accuracy. *Phys. Chem. Chem. Phys*. **2005**, *7* (18), 3297–3305. (d) Weigend, F. Accurate Coulomb-fitting basis sets for H to Rn. *Phys. Chem. Chem. Phys.* **2006**, *8* (9), 1057-1065.
